# Prediction of antigen-responding VHH antibodies by tracking the evolution of antibody along the time course of immunization

**DOI:** 10.3389/fimmu.2023.1335462

**Published:** 2024-01-16

**Authors:** Tomonari Matsuda, Yoko Akazawa-Ogawa, Lilian-Kaede Komaba, Norihiko Kiyose, Nobuo Miyazaki, Yusaku Mizuguchi, Tetsuo Fukuta, Yuji Ito, Yoshihisa Hagihara

**Affiliations:** ^1^ Research Center for Environmental Quality Management, Kyoto University, Otsu, Japan; ^2^ Biomedical Research Institute, National Institute of Advanced Industrial Science and Technology (AIST), Ikeda, Japan; ^3^ Division of Antibody Operations, ARK Resource. Co., Ltd., Kumamoto, Japan; ^4^ JSR Corporation, Tsukuba, Japan; ^5^ Graduate School of Science and Engineering, Kagoshima University, Kagoshima, Japan; ^6^ Biomedical Research Institute, National Institute of Advanced Industrial Science and Technology (AIST), Tsukuba, Japan

**Keywords:** antibody maturation, antibody repertoire analysis, single-domain antibody, antibody engineering, prediction of antigen-responding antibody

## Abstract

Antibody maturation is the central function of the adaptive immune response. This process is driven by the repetitive selection of mutations that increase the affinity toward antigens. We hypothesized that a precise observation of this process by high-throughput sequencing along the time course of immunization will enable us to predict the antibodies reacting to the immunized antigen without any additional *in vitro* screening. An alpaca was immunized with IgG fragments using multiple antigen injections, and the antibody repertoire development was traced *via* high-throughput sequencing periodically for months. The sequences were processed into clusters, and the antibodies in the 16 most abundant clusters were generated to determine whether the clusters included antigen-binding antibodies. The sequences of most antigen-responsive clusters resembled those of germline cells in the early stages. These sequences were observed to accumulate significant mutations and also showed a continuous sequence turnover throughout the experimental period. The foregoing characteristics gave us >80% successful prediction of clusters composed of antigen-responding VHHs against IgG fragment. Furthermore, when the prediction method was applied to the data from other alpaca immunized with epidermal growth factor receptor, the success rate exceeded 80% as well, confirming the general applicability of the prediction method. Superior to previous studies, we identified the immune-responsive but very rare clusters or sequences from the immunized alpaca without any empirical screening data.

## Introduction

Antibodies accumulate somatic hypermutations and undergo affinity maturation upon exposure to antigens (1). Immunization exploits this mechanism to produce antibodies against the target antigens. Repetitive antigen injections introduce random mutations and increase the antigen affinity of the antibodies. The history of the mutational changes that occur in antibodies during immunization directly reflects the enhancement of the adaptive humoral immune response. We hypothesized that it will be possible to screen the antibodies reacting to the immunized antigen by tracking the evolution of an antibody along the time course of immunization.

High-throughput next-generation sequencing (NGS) of vast immune repertoires provides useful information for immunological system research and its practical applications (2, 3). Unlike conventional sequencing techniques, NGS enables us to draw a comprehensive picture of immune repertoires that respond to antigens. The process of antibody development by immunization can be precisely examined by high-throughput sequencing of the samples collected during the course of immunization and reveals the time-resolved bird’s eye view of antibody maturation.

Prediction methods for antigen binding antibodies using sequence data from immunized animals have been developed based on the frequency of occurrence of the individual antibody sequences (4–6). The sequences are ranked by the number of sequence reads, and about 10 sequences at the top frequency of occurrence were picked as the candidates for objective antibody. The accuracy rates of this approach are quite high, where at least more than 75% selected candidates interacted with immunized antigen. The propensity-based approach is a simple and powerful way to discover antibodies from immunized animals, but by the very nature of this approach, infrequent antibodies are inherently omitted from the prediction.

It is difficult to link antibody repertoire development with the changes in protein level characteristic of antigen-responding antibodies. Despite the development of various empirical and bioinformatics technologies for nucleotide sequencing (7–9), correct light-chain and heavy-chain matching remains a challenging problem in the biophysical study of antibody obtained by high-throughput sequencing. Furthermore, the preparation of full-length antibody from NGS sequence reads requires time-consuming recombinant strain construction and mammalian cell culture. Small antibody formats such as single-chain F_v_ fragment (scF_v_) and F_ab_ can be produced by bacterial hosts. This approach may result in aggregation, defective folding, and loss of activity. The V_H_ domain of camelid heavy-chain antibody (VHH) binds the antigen in a single-domain format (10, 11) and can usually be produced rapidly, conveniently, and inexpensively in an *Escherichia coli* (*E. coli*) expression system (12). VHH is a suitable antibody format to examine numerous sequences and explore the physical effects of mutational changes induced by affinity maturation.

Here we report the *in silico* prediction method to identify the VHH antibodies reacting to the immunized antigen without any additional *in vitro* screening after immunization. We first carried out a series of experiments using human IgG fragments as antigens. Antibody repertoire development was studied using pools of peripheral lymphocytes collected from immunized alpaca blood periodically for months. The VHH sequences were clustered according to length and similarity and were analyzed for time-dependent mutational changes. The VHHs in the 16 most abundant clusters were produced and examined to determine whether they interacted with the immunized antigen. We then evaluated the evolutionary patterns of these clusters. In addition, to enhance our exploration of clusters comprising clones responsive to antigens, antigen-binding VHHs were identified by phage display from a library constructed from blood collected at 9 weeks post-immunization, and clusters containing such clones were also scrutinized (13). Using the features extracted from the examined clusters, data from alpacas immunized with IgG fragments were used to predict clusters consisting of VHH antibodies reacting to the antigen. To further confirm the effectiveness of the method, the prediction was applied to the other alpacas immunized with epidermal growth factor receptor (EGFR).

## Materials and methods

### Alpaca immunization

**Figure 1:**
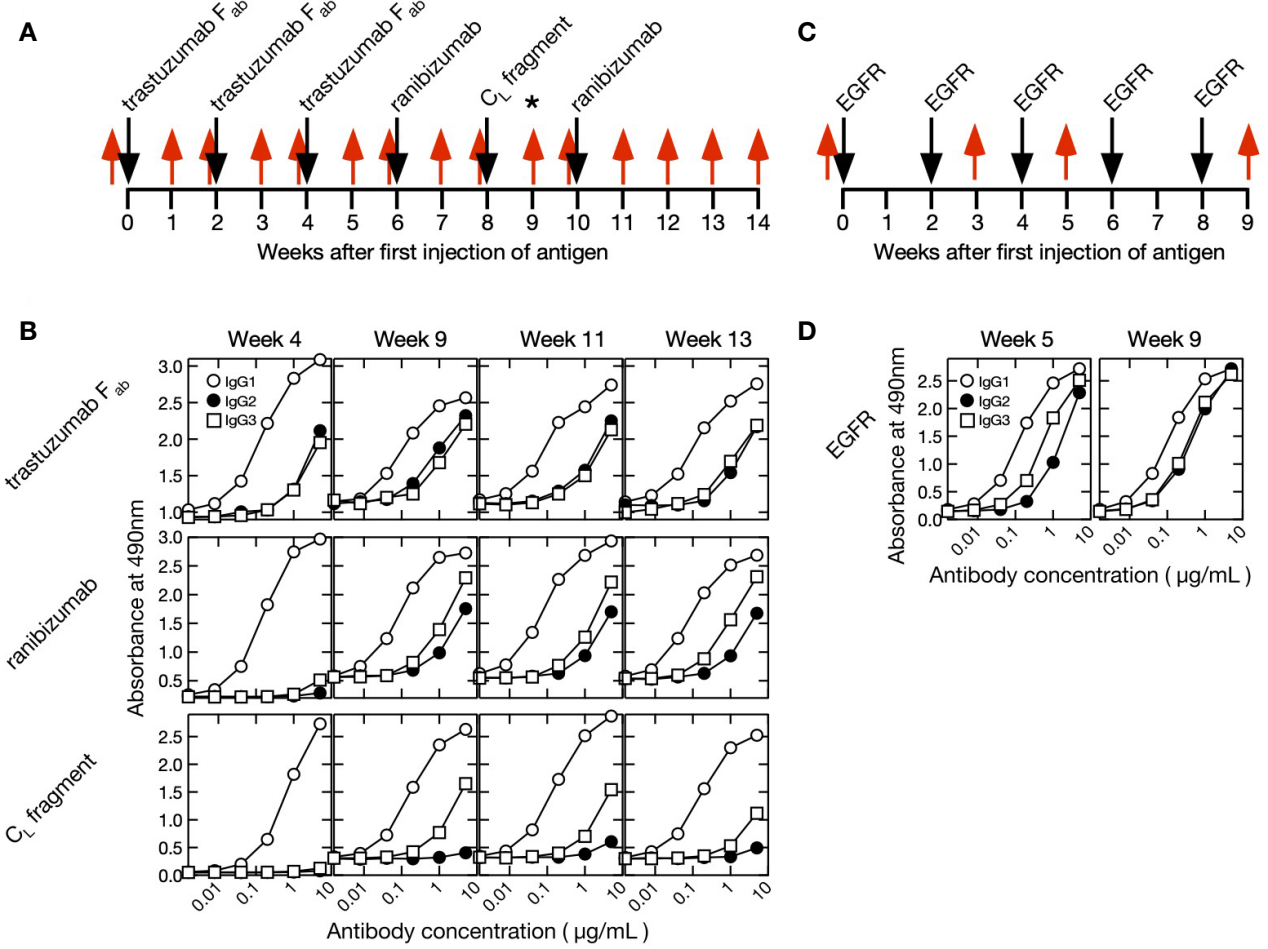
Immunization and blood collection schedules and time course of purified polyclonal alpaca antibody titer. **(A)** F_ab_ of trastuzumab, ranibizumab, and human κ C_L_ antigens were injected into a single alpaca. Blood was collected once before immunization (week 0) and 14× after initiating immunization (weeks 1–14). A phage display screening library was prepared using blood collected at week 9 as indicated by an asterisk (*). The timing of antigen immunization and blood collection are indicated by black and red arrows, respectively. **(B)** Polyclonal IgG1 (conventional antibody), IgG2 (heavy-chain antibody with short hinge), and IgG3 (heavy-chain antibody with long hinge) were purified from blood collected at weeks 4, 9, 11, and 13. Titers were measured against F_ab_ of trastuzumab, ranibizumab, and human κ C_L_ fragment. **(C)** Human epidermal growth factor receptor (EGFR) was injected into a single alpaca different from IgG fragments experiment at weeks 0, 2, 4, 6, and 8 (black arrow). Blood was collected at weeks 0, 3, 5, and 9 (red arrow). **(D)** Polyclonal IgG1, IgG2, and IgG3 were purified from blood collected at weeks 5 and 9. Titers were measured against EGFR.

An alpaca was immunized with 1.0–2.8 mg human IgG fragments every 2 weeks for a total of six treatments, and an alpaca different from the former was immunized with 0.5–2.0 mg human EGFR every 2 weeks for a total of five treatments (**Figure 1**).

The human IgG fragments used to immunize were F_ab_ from trastuzumab (Genentech, San Francisco, CA, USA), ranibizumab (Genentech), and human κ C_L_ (13). To prepare the trastuzumab F_ab_ fragment, trastuzumab (1.75 mg/mL) was treated with 1/10 volume immobilized papain (Thermo Fisher Scientific, Rockford, IL, USA) in Na_3_PO_4_ (20 mM), EDTA (10 mM), and cysteine (20 mM) at 37°C for 17 h. The samples were purified by cation exchange chromatography in a Resource S column (Cytiva, Tokyo, Japan) containing MES buffer (20 mM, pH 6). The samples were subjected to gel permeation chromatography in a HiLoad 26/600 Superdex 75 (Cytiva) in the presence of Na_3_PO_4_ (10 mM) and NaCl (150 mM, pH 7.1). Synthetic human κ C_L_ gene was cloned into pAED4 plasmid (14) which, in turn, was expressed in *E. coli* strain BL21 (DE3) pLysS (Agilent Technologies, Santa Clara, CA, USA). The inclusion body containing the κ C_L_ was dissolved in 6 M guanidine HCl, dialyzed against 1% (v/v) CH_3_COOH, and purified by reversed-phase high-performance liquid chromatography (RP-HPLC) (15).

A synthetic extracellular fragment of EGFR (amino acid 25–618 of mature EGFR) gene was designed using human-optimized codon frequencies. The EGFR gene with kozac and mouse signal peptide (MLDASGCSWAMWTWALLQLLLLVGPGGC) at N-terminus and hexahistidine tag (HisTag) at the C-terminus was cloned into pcDNA3 vector. 293T cells were purchased from ATCC (CRL-11268) and were maintained using DMEM supplemented with 10% fetal bovine serum. Transient transfection was performed with polyethylenimine reagent (PEI MAX, Polysciences, Warrington, PA, USA) according to the manufacturer’s protocol. For protein production, the 283T cells were grown in the DMEM medium containing 0.1% bovine serum albumin (BSA). A total of 40 µg plasmid DNA per 15-cm dish culture was diluted in fresh OPTI-MEM, 120 µg polyethylenimine was added, and the mixture was immediately vortexed and incubated for 20 min at 20°C–30°C prior to its addition to the cells. After 7 days, the medium was collected and dialyzed against Tris-HCl (20 mM, pH 8.0) at 4°C overnight. A His Trap HP column equilibrated with Tris-HCl (20 mM, pH 8.0) and NaCl (0.5 M) was used to purify the crude EGFR protein, and the later was eluted with imidazole. A Superdex 200 (10/300) column (Cytiva) equilibrated with phosphate-buffered saline (PBS) was used to purify the EGFR.

The concentrations of antigen proteins were determined by measuring the absorbance in an Ultraspec 3300 Pro spectrophotometer (Cytiva) at 280 nm (16).

Complete and incomplete Freund adjuvants (Becton, Dickinson and Company, Franklin Lakes, NJ, USA) were used after the first and subsequent immunizations, respectively. Blood collection (30–50 mL) began just before the first antigen injection and was performed weekly for 14 weeks for IgG experiments and at weeks 3, 5, and 9 for EGFR experiment. Lymphocytes were purified from 30–50 mL of blood by the Ficoll–Plaque method using Leucosep tubes (Greiner Bio-One, Frickenhausen, Germany). Purified lymphocytes were homogenized in RNAiso Plus (Takara Bio Inc., Kusatsu, Japan) and stored at -80°C until use. Total RNA was extracted from alpaca lymphocyte homogenate in RNAiso Plus according to the manufacturer’s protocol.

IgG subclasses were obtained by sequential affinity chromatography separation on Protein G and Protein A Sepharose columns (Cytiva) as previously reported (17). Plasma was subjected to 2× serial dilutions with PBS and applied to a Protein G Sepharose column to absorb IgG1 and IgG3. The column was washed with PBS, IgG3 was eluted with 0.58% (v/v) CH_3_COOH (pH 3.5) containing NaCl (0.15 M), and IgG1 was eluted with glycine-HCl (0.1 M, pH 2.7). The fraction excluded from the Protein G column was applied to a Protein A column to absorb IgG2. The column was washed, and the bound IgG2 was eluted with 0.58% (v/v) CH_3_COOH (pH 4.5) containing NaCl (0.15 M). All fractions were neutralized with Tris-HCl (0.1 M, pH 9.0), and their protein concentrations were determined by using bicinchoninic acid (BCA) assay (Thermo Fisher Scientific) according to the manufacturer’s protocol.

Each well of a 96-well plate (Maxisorp Nunc; Thermo Fisher Scientific) was coated with 100 µL of PBS solution containing 5 µg/mL antigen (ranibizumab, trastuzumab, or human κ C_L_ fragment), incubated at 4°C overnight, and blocked with 0.5% (w/v) gelatin in PBS. The plate was washed thrice with PBS containing 0.05% (w/v) Tween-20, and serially diluted alpaca serum or purified alpaca antibody was added. The plates were then incubated at 20°C–30°C for 60 min. To detect bound alpaca IgG, anti-alpaca IgG rabbit polyclonal antibody was added and the plates were incubated at 20°C–30°C for 60 min. Horseradish peroxidase (HRP)-conjugated anti-rabbit IgG goat antibody (Bio-Rad Laboratories, Hercules, CA, USA) was added to detect bound anti-alpaca IgG rabbit antibody. The wells were washed with PBS containing 0.05% (w/v) Tween-20. Bound antibodies were detected with the horseradish peroxidase (HRP) substrate *o*-phenylenediamine (Merck KGaA, Darmstadt, Germany). The reactions were stopped with 1 M H_2_SO_4_ after 20 min, and the absorbance was measured in a microplate reader (Benchmark; Bio-Rad Laboratories) at 490 nm.

### Construction of VHH phage library from alpaca immunized with IgG fragments

The method for constructing the alpaca VHH phage library was the same as described previously (13). Using total RNA from the lymphocyte of alpaca immunized with IgG fragments collected at week 9, cDNA was synthesized by reverse transcriptase with Oligo (dT)20 primer from 5 µg total RNA using the SuperScript III First-Strand Synthesis System for reverse transcription-PCR (Thermo Fisher Scientific). VHH gene was amplified using the common forward VHH-specific primer 5′-AGKTGCAGCTCGTGGAGTCNGGNGG-3′ and the reverse IgG2-specific primer 5′-GGGGTCTTCGCTGTGGTGCG-3′ or IgG3-specific primer 5′-TTGTGGTTTTGGTGTCTTGGG-3′. The initial PCR was executed employing KOD-Plus-Neo DNA polymerase (Toyobo Co., Ltd., Osaka, Japan). The reaction steps included initial denaturation (98°C for 2 min) followed by 22 repetitions of the three-step cycle: denaturation (98°C for 30 s), annealing (58°C for 30 s), and extension (72°C for 1 min). For the second PCR, aimed at incorporating restriction sites at both ends of the gene, the common forward primer containing the *Sfi*I site (5′-TGCTCCTCGCGGCCCAGCCGGCCATGGCTCAGGTGCAGCTCGTGGAGTCTGG-3′) and either the reverse IgG2-specific primer (5′-ATGATGATGTGCACTAGTGGGGTCTTCGCTGTGGTGCG-3′) or the reverse IgG3-specific primer (5′-ATGATGATGTGCACTAGTTTGTGGTTTTGGTGTCTTGGG-3′), both carrying the *Spe*I site, were utilized. The second PCR was performed using Gene Taq DNA polymerase. (Nippon Gene Co., Ltd., Tokyo, Japan). VHH libraries were constructed using the phagemid vector pKSTV-022, which has *Sfi*I and *Spe*I sites to integrate the VHH gene position behind the lac promoter and pelB signal sequence (17).

### Biopanning against IgG fragments

Three methods of antigen capture onto solid phase were used to select the IgG fragment-binding clones. The first and second methods were the same as described previously (13). First, 200 µg of F_ab_ of trastuzumab and ranibizumab was biotinylated using the Lightning-Link Rapid Biotin Conjugation Kit (Abcam Inc., Cambridge, UK). Then, 20 µg of biotinylated F_ab_ fragment of trastuzumab or ranibizumab was incubated with phage libraries [1.0–10^11^ plaque-forming units (pfu)] in PBS containing 0.5% BSA for 2 h. Subsequently, the mixture was added to microtiter plate wells (Nunc Thermo Fisher Scientific) that had been coated with streptavidin (SA) (500 ng in 200 µL PBS). After 2 h of incubation, the wells were washed 10 times with PBS (PBST) containing 0.1% Tween-20. Then, 0.1 M glycine-HCl (pH 2.2) was added to elute antigen-specific phages. After neutralization, the eluted phages were infected with *E. coli* TG-1. For the next biopanning, phages were rescued by infection of M13 KO7 helper phage. In the second capture method, recombinant human ErbB2/Her2-Fc protein (R&D systems, Minneapolis, MN, USA) dissolved in PBS (500 ng/200 µL) was coated onto 96-well microtiter plates (Nunc Thermo Fisher Scientific) wells and blocked with a blocking solution. Phage library solution (1.0–10^11^ pfu) mixed with 200 µL of trastuzumab (500 ng) in blocking solution was added to the wells coated with Her2-Fc to trap trastuzumab complexed with the phage. After 2 h of incubation, the plate was washed 10 times with PBST. The phage were then eluted, neutralized, and infected with *E. coli* TG-1. In the third method, F_ab_ of trastuzumab (500 ng in 200 µL PBS) was added to microtiter plates (Nunc Thermo Fisher Scientific) and allowed to stand for 2 h to directly solidify the antigen, followed by blocking with PBS containing 0.5% BSA. Phage library solution (10^9^ pfu) was added to the plate, and after 1 h of reaction, the plate was washed five times with PBST and 0.1 M glycine-HCl (pH 2.2) was added to elute the phage. The sample was neutralized and infected with *E. coli* TG-1.

### Library preparation and NGS analysis

cDNA was synthesized by reverse transcriptase using oligo(dT)20 primer from 5 µg total RNA by the SuperScript III First-Strand Synthesis System for RT-PCR (Thermo Fisher Scientific). The NGS libraries for MiSeq (Illumina, San Diego, CA, USA) were constructed by three-step PCR amplification. The first PCRs were performed to amplify the IgG2 and IgG3 sequences from the cDNA. The primer sequences used for the first PCR were 5′-CAGGTGCAGCTCGTGGAGTCTGG-3′ (forward primer for both IgG2 and IgG3), 5′-GGGGTCTTCGCTGTGGTGCG-3′ (reverse primer for IgG2), and 5′-TTGTGGTTTTGGTGTCTTGGGTTC-3′ (reverse primer for IgG3). The second PCR was run to add the adaptor sequence. The primer sequences were 5′-GTCTCGTGGGCTCGGAGATGTGTATAAGAGACAGCAGGTGCAGCTCGTGGAGTCTGG-3′ (forward primer for both IgG2 and IgG3), 5′-TCGTCGGCAGCGTCAGATGTGTATAAGAGACAGGGGGTCTTCGCTGTGGTGCG-3′ (reverse primer for IgG2), and 5′-TCGTCGGCAGCGTCAGATGTGTATAAGAGACAGTTGTGGTTTTGGTGTCTTGGGTTC-3′ (reverse primer for IgG3). The third PCR was conducted to add the index and the p5 and p7 sequences required for the NGS reaction. Nextra XT Index Kit v. 2 (Illumina) was the primer source. The PCR were performed with KOD-Plus-Neo DNA polymerase (Toyobo Co., Ltd.). The PCR program was as follows: initial denaturation (98°C for 2 min) followed by several denaturation cycles (98°C for 10 s), annealing (58°C for 30 s), and extension (68°C for 20 s). There were 22 cycles for the first PCR and eight cycles each for the second and third PCRs. The library was sequenced for 600 cycles using the reagents in MiSeq Reagent Kit v. 3 (Illumina).

### Software

Most of the data processing was conducted in R v. 3.4.4 (https://www.r-project.org). The R packages installed for this analysis were “dplyr”, “stringr”, “msa”, “ape”, and “sna”.

### Merged VHH sequence read generation

The NGS data were demultiplexed into 30 (IgG fragments immunization) and eight (EGFR immunization) datasets comprising 15 (IgG fragments immunization) and four (EGFR immunization) time-course data points and two antibody types (IgG2 and IgG3). The sequence reads in each dataset were quality-trimmed (limit = 0.01), and their overlaps were merged (mismatch cost = 2; minimum score = 8; gap cost = 3) with CLC Genomics Workbench v. 7.5.1 (QIAGEN, Venlo, The Netherlands). The 3′ ends of the merged sequences were trimmed (IgG2: 21 bases; IgG3: 24 bases) to remove the sequences in the constant region. The merged VHH sequence reads were summed to generate a data frame consisting of the columns “unique sequence” and “frequency” and named “sequence–frequency table.” Unique sequences containing ambiguous base calls, lacking lengths in multiples of three, or unable to encode VHH peptides were removed from the dataset. Sequence analyses, bit score estimations, and clustering were performed based on DNA rather than amino acid sequences.

### Sequence error cleanup

Random errors occur during library preparation and NGS sequencing. To eliminate them, the “sequence–frequency table” was sorted in descending order of “frequency”, and the most common sequence was selected and defined as a “reference sequence” (RS). The “threshold for error number” (*n*) was then configured (*n* = 3 when the RS frequency was in the range of 2–400, *n* = 4 when the RS frequency was in the range of 401–1,000, and *n* = 5 when the RS frequency was >1,000). This threshold ensured that RS-derived errors were likely to appear based on the Poisson distribution and the RS frequency. Unique sequences having ≤*n* (including 0) base changes compared with the RS were extracted from the “sequence–frequency table” and designated the “dataset for integration” consisting mainly of RS-derived sequences with errors. However, certain independent sequences and their derivatives could also be included in the dataset. Hence, the “threshold for independence” (*r*) was configured to remove them. If the frequency ratio of a particular sequence and RS exceeded the threshold, the sequence was considered independent and was removed from the “dataset for integration.” Derivative sequences were those having the same differential patterns as their corresponding independent sequences and were also removed from the dataset. The threshold *r* value was arbitrarily set and configured according to the number of base changes in the independent sequence (*r* = 8% when there was only one base change, *r* = 3% when there were two base changes, *r* = 1% when there were three base changes, and *r* = 0.2% when there were at least four base changes). To obtain the major independent sequences, *r* was set to a value exceeding the expected error rate. The remaining sequences in the “dataset for integration” were derived from RS, and their frequencies were summed to an integrated RS frequency. The sequence data in the “dataset for integration” were removed from the “sequence–frequency table.” The most common sequence in the updated “sequence–frequency table” was defined as a new RS. The foregoing integration procedures were repeated until the RS frequency = “1”. All RS and their integrated frequency data were combined with the remaining “sequence–frequency table” to generate a clean iteration.

### Chronological data combination and sequence ID generation

The clean “sequence–frequency tables” for IgG2 (short-hinge antibody) at each time point were combined using the “full-join” command in the “dplyr” package of R. In this manner, a data table was created. It consisted of 16 (IgG fragments immunization) and five (EGFR immunization) columns including “unique sequence” and their frequencies at 15 (IgG fragments immunization) and four (EGFR immunization) time points. The column “maximum frequency” representing the maximum frequencies at 15 (IgG fragments immunization) and four (EGFR immunization) time points per sequence was added, and the data table was sorted in descending order of “maximum frequency”. The sequence IDs were configured as “S1” (short-hinge antibody 1), “S2” (short-hinge antibody 2), “S3” (short-hinge antibody 3), and so on. The same methodology was applied to IgG3 (long-hinge antibody), and its sequence IDs were configured as “L1” (long-hinge antibody 1), “L2” (long-hinge antibody 2), “L3” (long-hinge antibody 3), and so on. The data tables for IgG2 and IgG3 were vertically combined and designated the “chronological sequence–frequency table”.

### Original V and J sequence estimation

NCBI BLAST was used to estimate the original *IGHV* and *IGHJ* sequences of the VHH sequences (18) (https://blast.ncbi.nlm.nih.gov/Blast.cgi). References for the alpaca genomic sequences of *IGHV* and *IGHJ* were obtained from the IMGT database (http://www.imgt.org/). BLAST databases for each alpaca *IGHV* and *IGHJ* were constructed with the “makeblastdb” command in BLAST. The sequences in the “chronological sequence–frequency table” were BLAST-searched against the databases. Hit *IGHV* and *IGHJ* showing the smallest e-values were deemed original sequences. Similarities among VHH sequences and their original genomic sequences were described using the “bit score” command in BLAST.

### U40 (under 40) calculation

Here a new parameter “U40” was defined, and it represented the “loneliness” of the sequences in the dataset. U40 was defined as the number of unique sequences differing by fewer than 40 base pairs from the reference sequence. To calculate U40, sequences equal in length to the reference sequence were extracted from the “chronological sequence–frequency table,” the differences between the reference sequence and each of the extracted sequences were calculated, and the sequences differing from the reference by fewer than 40 bp were enumerated.

### Cluster isolation

A molecular phylogenetic tree was constructed to isolate antibody sequence clusters. The R packages “msa”, “ape”, and “sna” were used in this analysis. The sequences in the dataset were grouped according to a combination of sequence lengths and *IGHV* and *IGHJ* types. Sequences 333 bp long and derived from *IGHV3S53* and *IGHJ4* were classified in the “333-IGHV3S53-IGHJ4 group.” Various sequences derived from a single ancestor and those derived by affinity maturation belong to the same group. Hence, molecular phylogenetic analysis was performed on each group. To simplify it, minor sequences with maximum frequency = 1 were excluded from the data for each group. The exceptions were clones identical to those obtained by phage display. These were included in the figures to indicate their position in the phylogenetic tree. We did not focus on the “lonely” sequences. Therefore, those with U40 <10 were also excluded from the analysis. Moreover, groups with fewer than eight unique sequences were removed. Exclusion of noisy sequence data conserves computational resources for molecular phylogenetic analyses and facilitates accurate cluster separation.

To partition the phylogenetic tree into several clusters, a distance threshold value of 0.04 was set, and all distance values surpassing it in the distance matrix were set to zero. Links between clusters were disconnected in the replaced distance matrix. To extract the connected clusters from the replaced distance matrix, the “component.dist” command of the “sna” package was used. Isolated clusters containing more than seven sequences were assigned cluster IDs, and the “cluster_ID” column was added to the “chronological sequence–frequency table.” For the subsequent analysis, clusters were discarded if they included sequences expressed before immunization.

### VHH preparation

A synthetic VHH gene was designed using codon frequencies optimized for *E. coli*. The VHHs were cloned into pAED4. The proteins were expressed in *E. coli* BL21(DE3) pLyS, and they accumulated in inclusion bodies. The latter was dissolved in a mixture of guanidine HCl (4 M), dithiothreitol (DTT; 10 mM), and Tris-HCl (10 mM, pH 8.5). The solution was left to stand at 25°C for >3 h. A HisTrap HP column (Cytiva) equilibrated with urea (6 M), Tris-HCl (20 mM, pH 8.5), and NaCl (0.5 M) was used to purify the crude VHH protein and the latter was eluted with imidazole. The samples were subjected to air oxidation at 4°C overnight and dialyzed against Tris-HCl (10 mM, pH 8.0). Then, 1/10 volume sodium acetate (1 M, pH 4.7) was added to the samples, and the latter were dialyzed against sodium acetate (10 mM, pH 4.7). A Resource S cation-exchange column (Cytiva) equilibrated with sodium acetate (10 mM, pH 4.7) was used to purify the VHHs. The VHH concentration in the stock solution was determined by measuring the absorbance with an Ultrospec 3300 Pro spectrophotometer (Cytiva) at 280 nm (16).

### VHH antigen-binding activity measurement by surface plasmon resonance

For surface plasmon resonance (SPR) analysis, the antigen was immobilized on the sensor chip as a ligand, and the VHH sample was injected as an analyte. According to the manufacturer’s instructions, F_ab_ from trastuzumab, ranibizumab, human κ C_L_, and human EGFR were amine-coupled to a CM5 sensor chip (Cytiva) at 25°C using 10 µg/mL protein in sodium acetate buffer (20 mM, pH 4.7). Antigen proteins in sodium acetate (10 mM, pH 4.7) were immobilized to 1,000 resonance units (RU). The dilution series of the analytes was set to 1/2. The analysis was performed on a Biacore X100 instrument (Cytiva) in HEPES (10 mM, pH 7.4), NaCl (150 mM), EDTA (3 mM), and 0.005% (w/v) P20 surfactant (HBS-EP, Cytiva) at 20°C. The association reaction was monitored by injecting the sample at various concentrations onto the sensor chip. The dissociation reaction was performed by eluting the bound antigen with HBS-EP buffer. All experiments were performed at a flow rate of 10 µL/min, association time of 360 s, and dissociation time of 800 s. The sensor chip was regenerated with glycine-HCl buffer (10 mM, pH 2.0 for IgG fragments or pH 3.0 for EGFR experiments) containing NaCl (0.5 M) and equilibrated with HBS-EP buffer. The reference was the value of a channel with no ligand bound, and buffer (HBS-EP) measurement was performed as a blank before the new analyte measurement. Sensorgrams were subjected to kinetic analysis using BIA evaluation software (Biacore X100 Evaluation Software, Cytiva). The “1:1 binding model” was used for determining dissociation constants (*K*_D_).

### Enzyme-linked immunosorbent assay

Antigen protein (100 µL, 10 µg/mL) in carbonic buffer (50 mM, pH 9.5) was immobilized on Maxisorp 96-well plates (Thermo Fisher Scientific) and incubated at 4°C overnight. At all stages, the wells were washed 4× with PBS (150 µL) containing 0.02% (w/v) Tween 20 (PBS-T). Unreacted sites on the plastic surfaces were blocked with 3% (v/v) bovine serum albumin (BSA)-PBS-T at 20°C–30°C for 1 h. Then, 100 µL VHH sample diluted in PBS-T with 0.1% (v/v) BSA was added to each well. The wells were incubated at 37°C for 2 h, then 100 µL anti-alpaca IgG rabbit antibody in 0.1% (v/v) BSA-PBST was added to each well and incubation continued at 37°C for 1 h. Then, 100 µL of horseradish peroxidase (HRP)-conjugated anti-rabbit IgG goat antibody in PBS-T containing 0.1% (v/v) BSA was added to each well, and incubation was continued at 37°C for 1 h. Then, 100 µL of 3, 3′5,5′-tetramethylbenzidine (TMB) (SeraCare Life Sciences, Milford, MA, USA) was added to each well, and incubation was continued at 20–30°C for 5 min. The reactions were stopped by adding 100 µL H_2_SO_4_ (0.5 M) to each well, and absorbances were measured in a microplate reader (Multiskan FC; Thermo Fisher Scientific) at 450 and 620 nm.

## Results

### Alpaca polyclonal antibody immune responses against injected antigens

In the two series of experiments, we immunized two different alpaca against either IgG fragments (F_ab_ from trastuzumab, ranibizumab, and a human κ C_L_) or human EGFR (**Figure 1**). The former experiment was designed to obtain antibodies against the constant region of human Fab, specifically the C_L_ domain, and thus we immunized the alpaca with three different antigens. The animals had already been immunized with the same adjuvants before this experiment. First blood samples (week 0) were collected immediately before immunization of IgG fragments or EGFR, and their sera were found to show no significant interaction with the immune antigens (**Supplementary Figure S1**). After the initial immunizations, blood samples were collected weekly for 14 weeks in the IgG fragments experiment and three times for 9 weeks in the EGFR immunization experiment. The subclass titers of purified IgG1 (conventional antibody consisting of light and heavy chains), IgG2 (heavy chain antibody with a short hinge between VHH and C_H1_) and IgG3 (heavy chain antibody with a long hinge between VHH and C_H1_) were measured (**Figure 1**). The VHH cDNAs were synthesized by reverse transcription of mRNAs extracted from a lymphocyte pool. Short-hinge and long-hinge VHH-specific primers were used, and the cDNAs were amplified for sequencing.

### Analysis of VHH sequence clusters from an immunized alpaca by IgG fragments

**Figure 2:**
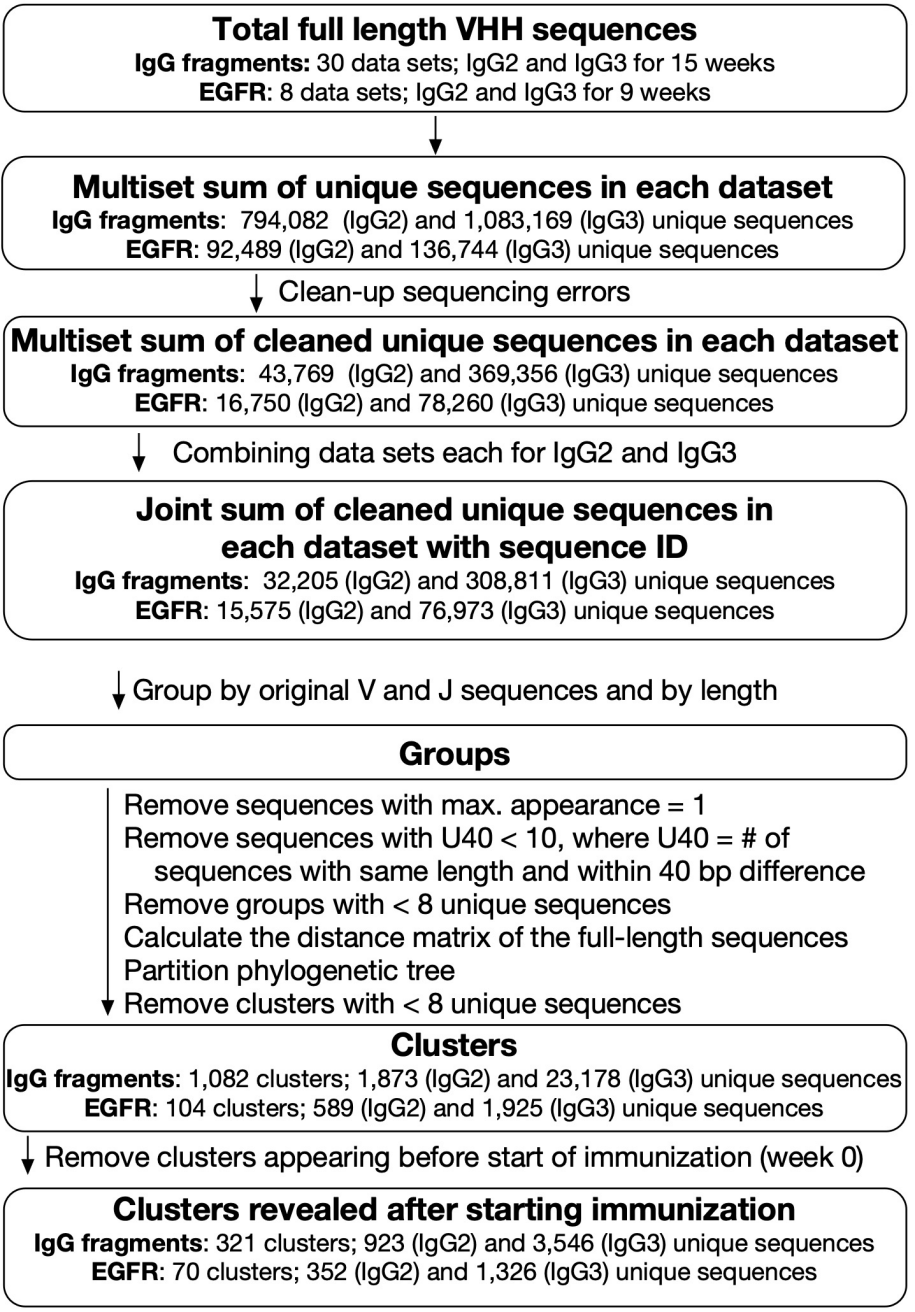
Analysis of next-generation sequencing data for VHH sequences. Blood samples were collected weekly for 15 weeks for IgG fragments experiment and at weeks 0, 3, 5, and 9 for epidermal growth factor receptor (EGFR) experiment. For IgG fragments experiment, 30 datasets for IgG2 and IgG3 sequences were combined and categorized into 321 clusters, including 923 and 3,546 unique sequences derived from IgG2 and IgG3, respectively. For EGFR experiment, eight datasets for IgG2 and IgG3 sequences were combined and categorized into 70 clusters, including 352 (IgG2) and 1,326 (IgG3) unique sequences.

First, we analyzed the sequence from an immunized alpaca by IgG fragments. After merging the overlaps, we obtained an average of 169,000 and 161,000 full-length VHH sequences from IgG2 and IgG3, respectively (**Table 1**) for IgG fragments immunization at each blood collection. The same sequences were gathered into “unique sequences” and cleaned of any sequence errors. At each time point, we obtained about 3,000 (IgG2) and 25,000 (IgG3) unique sequences on average. The sequences were grouped according to their germ-line V and J combinations and their lengths (**Figure 2**). Here we refer to a set of DNA sequences as a “group” potentially consisting of various antibody families. D-region data were not used for grouping as they were too short and introduced ambiguity into the sequence matching. It was assumed that, in most cases, the sequence lengths were the same for all members of each antibody family propagated from a single ancestral sequence to adapt a specific antigen.

**Table 1:** Number of total VHH sequences, unique sequences and cleaned unique sequences after integration of sequencing erros.

Antigen	Antibody type	Weeks	Total full-length VHH sequences	Unique sequences	Unique sequences after clean-up
IgG fragments	IgG2	0	211,351	69,038	2,098
		1	160,981	53,189	2,529
		2	187,660	58,121	3,015
		3	115,057	36,574	3,031
		4	196,922	61,371	2,952
		5	147,078	50,297	4,729
		6	161,048	52,735	3,855
		7	169,328	54,266	3,539
		8	190,859	56,035	3,056
		9	135,610	46,021	4,590
		10	154,433	44,416	2,560
		11	147,250	46,765	1,387
		12	203,289	61,877	2,497
		13	173,186	49,978	1,838
		14	176,914	53,399	2,093
		average	168,731	52,939	2,918
		total	2,530,966	794,082	43,769
	IgG3	0	163,313	80,561	33,198
		1	144,404	72,725	36,710
		2	166,321	80,269	35,991
		3	140,283	66,455	30,494
		4	85,113	41,371	17,529
		5	153,694	64,999	20,541
		6	143,035	64,101	24,147
		7	187,948	75,770	20,924
		8	164,175	73,442	25,736
		9	212,731	89,352	25,301
		10	190,428	86,395	28,576
		11	190,229	75,564	11,359
		12	124,633	56,590	17,174
		13	141,514	64,019	19,516
		14	209,026	91,556	22,160
		average	161,123	72,211	24,624
		total	2,416,847	1,083,169	369,356
EGFR	IgG2	0	116,144	34,801	7,975
		3	75,627	19,508	3,336
		5	80,190	19,800	2,815
		9	68,392	18,380	2,624
		average	85,088	23,122	4,188
		total	340,353	92,489	16,750
	IgG3	0	63,800	38,704	30,582
		3	59,462	29,446	17,214
		5	95,023	39,107	16,589
		9	71,617	29,487	13,875
		average	72,476	34,186	19,565
		total	289,902	136,744	78,260

After excluding lone sequences, the DNA sequences within a group were divided by phylogenetic tree analysis into “clusters”. We hypothesized that the clusters had the properties in which the sequences bound the same antigens and shared the same ancestors. However, it was difficult to conclude that each cluster covers entire sequences that evolved from the initial sequence. Moreover, we could not rule out the possibility that each cluster contained sets of different antibodies recognizing various antigens. Before the experiment, the alpaca used here had already been immunized with the same adjuvants. Therefore, we discarded clusters including sequences expressed before immunization and assumed that any clusters interacting with the adjuvants were removed at this step. We obtained 321 clusters comprising 923 and 3,546 sequences derived from IgG2 and IgG3, respectively. Numerous identical sequences were observed in IgG2 and IgG3. Hence, the total number of unique sequences was, in fact, less than the sum of the unique sequences derived from IgG2 and IgG3. The numbers in cluster ID refer to the descending order of maximum sum of the frequencies of included clones. In terms of V gene and J gene utilization, the prevailing combinations were *IGHV3S53*, *IGHV3S66*, or *IGHV3S61*, coupled with *IGHJ4* or *IGHJ6*, both before and after immunization (**Supplementary Table S1**). These types of usage do not significantly contradict the findings of a recent extensive analysis of the camelid naïve library (19).

### Characteristics of clusters containing VHH sequences that bind to IgG fragments

We attempted to elucidate the characteristics of the 16 predominant clusters showing the highest maximum percentage appearance. The percentage appearance is the sum of the percentage occupancy of the IgG2 and IgG3 sequences in the cluster relative to all IgG2 and IgG3 sequences per week. The maximum percentage appearance is the largest value among the cluster’s percentage appearances for each week, i.e., among 14 values, during the immunization period. The sequences for clusters Ig-7 and 15 were identified by bio-panning the M13 phage library for the cDNA of the VHHs collected at week 9. We also examined clusters Ig-69, Ig-99, and Ig-210, which were identified by bio-panning, and cluster Ig-33, which was identified by comparing the sequence propensities before and after bio-panning (13, 17). We prepared VHH proteins for all 20 clusters (**Supplementary Table S2**) and used enzyme-linked immunosorbent assay (ELISA) and SPR to evaluate their antigen affinities (**Supplementary Figure S2**).

Only the VHH clones in clusters Ig-2, Ig-5, Ig-7, Ig-10, Ig-15, and Ig-16 exhibited antigen binding (“hit-clusters”). No antigen binding was detected for the clones in clusters Ig-1, Ig-3, Ig-6, Ig-8, Ig-9, Ig-11, Ig-12, Ig-13, or Ig-14 (“miss-clusters”). The SPR results revealed that clone Ig-S38 in cluster Ig-4 bound aberrantly to the C_L_ fragment. Thus, cluster Ig-4 could not be designated an antigen-binding clone and was excluded from further analysis. All clones had the same nomenclature as the sequence ID. The initial S and L indicate sequences derived from short-hinge antibody (IgG2) and long-hinge antibody (IgG3), respectively. The numbers following the S and L in the sequence ID refer to the descending order of maximum number of sequence appearance per week.

The ELISA and SPR results showed that clones Ig-S11 (cluster Ig-2), Ig-L926 (cluster Ig-15), Ig-L792 (cluster Ig-16), and Ig-L252126 (cluster Ig-99) exhibited affinity for F_ab_ from trastuzumab and ranibizumab. Thus, the epitopes of these clones constituted the F_v_ and/or C_H1_ domain framework regions. The ELISA and SPR signals of clone Ig-L2477 (cluster Ig-69) were positive for the C_L_ fragment and F_ab_s, suggesting that the epitope of this clone is located on the C_L_ domain. In other clones, a different reactivity was observed in ELISA and SPR. We consider that the main reason for the discrepancy between the ELISA and SPR results is due to the different conformational states of the antigen in the two assays: most adsorbed proteins on the plastic surface are partially or largely denatured (20), while the SPR sensor chip surface induces less conformational change of the protein. Clone Ig-L19 (cluster Ig-10) only produced an ELISA signal against F_ab_s. Its epitope may have been buried inside the antigen protein and then exposed by its interaction with the plastic surface. There were ELISA signals in clones Ig-L38 (cluster Ig-5) and Ig-L15235 (cluster Ig-210) against all antigens. However, the SPR signal was against the C_L_ fragment alone. We hypothesized that these epitopes occurred on the C_L_ side of C_L_ and C_H1_ binding surfaces and were exposed by denaturation induced by the plastic surface. Clones Ig-S1139 (cluster Ig-7) and Ig-L54 (cluster Ig-33) had profiles resembling those of clones above recognizing F_v_ and/or C_H1_ domain framework regions. However, at high VHH concentrations, the ELISA signal against the C_L_ fragment was observed. We conceive that these clones recognize epitope on the surface of the F_v_ and/or C_H1_ domain as well as buried epitope in the native structure of the C_L_ domain. This epitope may only appear when the protein interacts with the plastic surface and undergoes denaturation.

**Figure 3:**
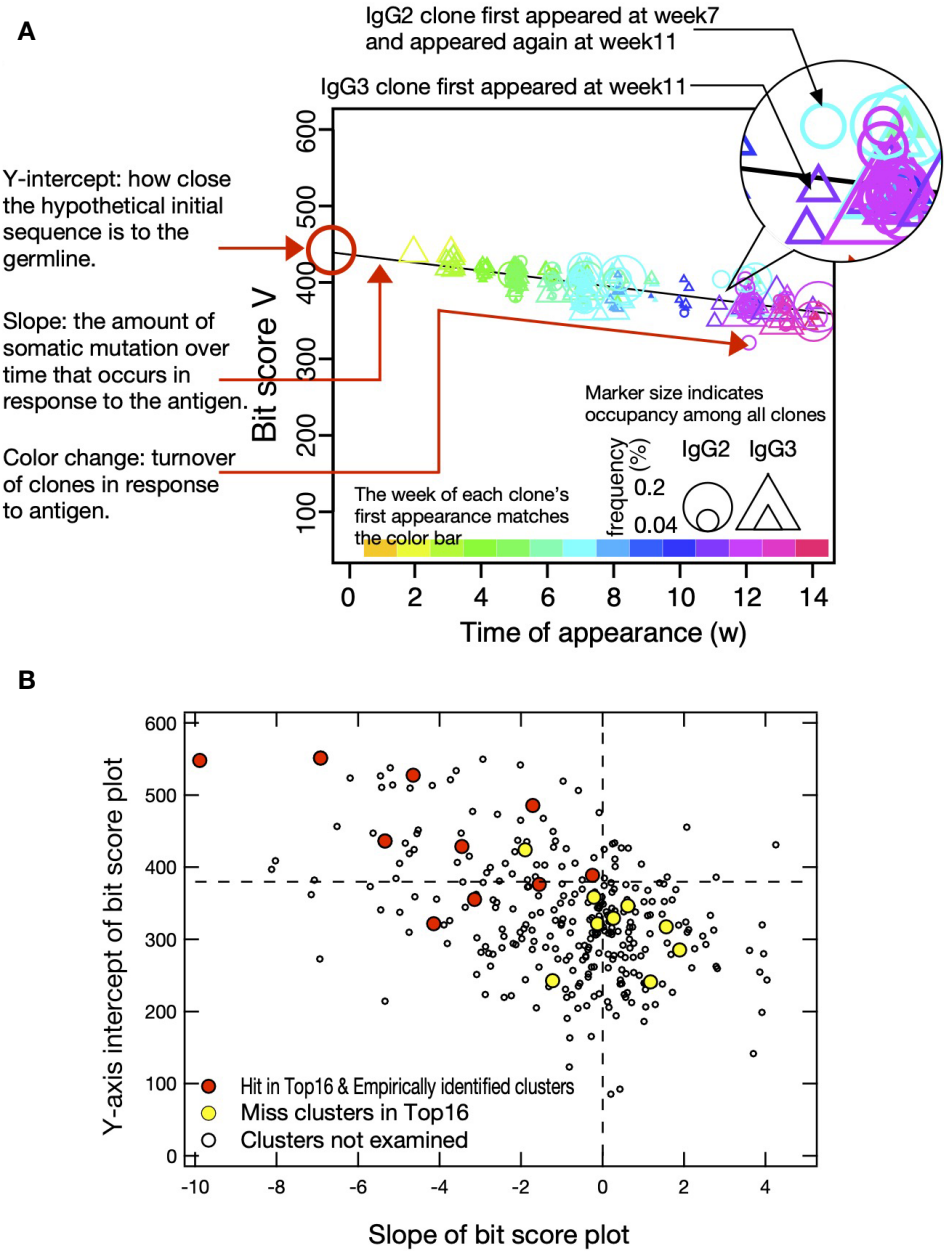
Bit score plot of cluster and relationship between slope of plot and Y-axis intercept. **(A)** An example of a bit score plot of cluster Ig-15 as an example of a typical hit-cluster. The color of the marker indicates the week of first appearance of each clone according to the color tone shown at the top of the x-axis, and the circles and triangles indicate that the clones appeared as IgG2 and IgG3, respectively, in the week indicated by the marker. Marker size indicates occupancy among all clones, and Y-axis intercept shows how close the hypothetical initial sequence is to the germline. Slope reflects the amount of somatic mutation over time that occurs in response to the antigen. Color change indicates turnover of clones in response to the antigen. **(B)** Y-axis intercept of bit score plot and slope of bit score plot for clusters obtained from IgG fragments immunization experiments. Red circles indicate hit-clusters and empirically identified clusters in the top 16, and yellow circles indicate miss-clusters in the top 16. The dotted lines on the X- and Y-axis indicate 0 and 380, respectively, which were used as thresholds for predicting hit-clusters.

**Figure 4:**
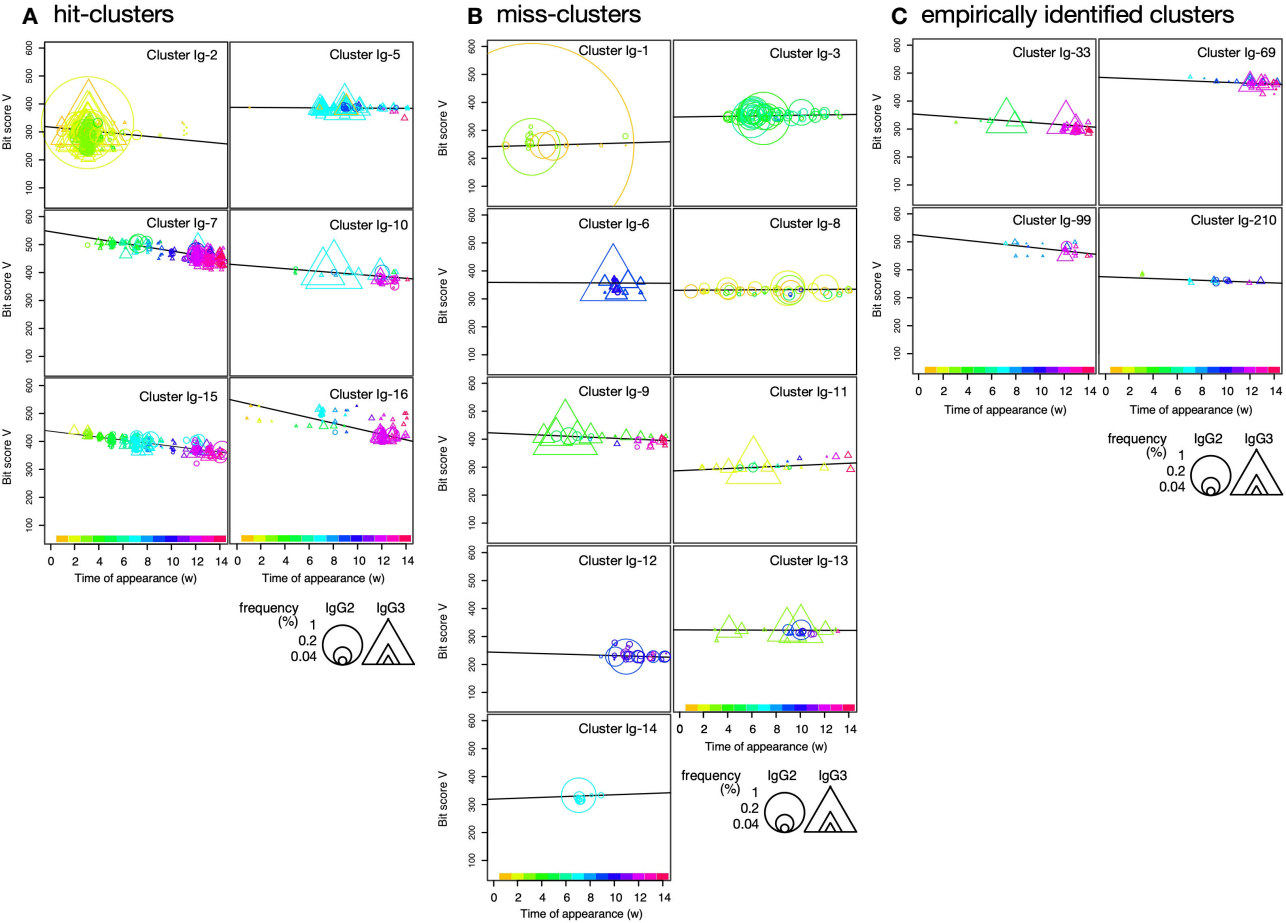
Plot of sequence bit scores vs. time of appearance of sequences in hit-clusters **(A)**, miss-clusters **(B)**, and empirically identified clusters **(C)** in IgG fragments immunization. Symbol colors indicate times of first appearance of each sequence. Colors corresponding to times of first appearance are indicated in the color bar at the panel bottom. Circles and triangles indicate sequences observed in short-hinge (IgG2) and long-hinge (IgG3) antibody, respectively. Symbol size corresponds to weekly clone frequency in IgG2 and IgG3 sequences.

To visualize cluster propagation in each independent sequence, we evaluated sequence appearance/disappearance transition and timing in the hit-clusters and miss-clusters (**Figures 3A**, **4A, B**). The cluster sequence transition was evaluated using the bit score parameter in the Basic Local Alignment Search Tool (BLAST) (18). It plotted the distance between the V gene region of each sequence and the ancestral germinal V gene sequence. The bit score increases with similarity of the query sequences to the reference. The relationship between bit score and the number of amino acid mutations in the V gene region is shown in **Supplementary Figure S3A**. Although there are deviations due to nonsense mutations, insertions, and deletions, it is assumed that there are generally 5, 10, 15, and 20 amino acid mutations at bit scores of 500, 450, 400, and 350, respectively, compared to the predicted amino acid sequence from germline V gene. All hit-clusters had negative slopes for bit score vs. time of sequence appearance. Thus, the sequences in the clusters interacting with the antigen continually changed and became more remote from the ancestral sequence during immunization. By contrast, five of the miss-clusters (Ig-1, Ig-3, Ig-8, Ig-11, and Ig-14) showed positive slopes for bit score vs. time of sequence appearance. Clusters Ig-6, Ig-9, Ig-12, and Ig-13 exhibited slightly negative slopes for bit score vs. time of sequence appearance (**Figure 4B**). Negative bit score slopes indicated sequence evolution and were good indicators of clusters that include antigen-binding clones (**Figure 3B**).

The Y-axis intercept at the onset of the experiment (initial bit score) pertains to the progression of the immune response against a new antigen (**Figure 3A**). For the hit- and miss-clusters, the averaged initial bit scores were 446 ± 90 and 319 ± 58, respectively. The alpaca was not pre-exposed to the antigens used in this experiment. Therefore, the hit-cluster sequences should not have been optimized before immunization and should have resembled the ancestral germinal V gene sequences. By contrast, clusters with low initial bit scores were unresponsive to the immunized antigens as they might have consisted of sequences that had matured before immunization.

Most hit-clusters displayed continuous sequence turnover, which is displayed by the change in symbol color indicating the timing of the first appearance of each sequence (**Figures 3A**, **4A**). This effect was clearly seen in clusters Ig-7, Ig-10, Ig-15, and Ig-16. Cluster Ig-2 disappeared at the late immunization stage even though it predominated at the early immunization stage. In cluster Ig-5, an early sequence occurred at week 1, reappeared at weeks 7 and 9, and disappeared thereafter (yellowish triangles). Sequences that had predominated between weeks 7 and 10 had persisted after 12 weeks. However, new sequences also emerged. Sequence turnover was only evident for miss-clusters Ig-9 and Ig-11. New sequences replaced old ones especially after week 12. The same was true for hit-cluster Ig-5. Distinct sequence turnover may be a hallmark of clusters affected by the immune response. However, it was not clear in clusters Ig-5, Ig-9, or Ig-11.

Four clusters, including the empirically identified clones, were analyzed by the same plot (**Figure 4C**). Negative bit score slopes, sequence turnover, and high initial bit scores were observed in all cases. These findings underscore the relationships among the hit-clusters and the foregoing criteria.

As a reference for tracking the accumulation of mutations involved in affinity maturation, time-dependent logo plots illustrating the typical hit- and empirically identified clusters (Ig-7 and Ig-69), as well as the miss-cluster (Ig-13), are presented in **Supplementary Figures S3B–D**. These figures depict the amino acid sequences of clones in clusters Ig-7 and Ig-63 comprising antigen-responsive antibodies, exhibiting an increased diversity in amino acids, particularly in CDRs, in contrast to Ig-13, which represents a miss-cluster. However, it is difficult to capture the time course of accumulation of mutations in individual sequences and sequence turnover in such a sequence-based representation, and we consider that the bit score plot is superior in that it is more intuitive and comprehensive.

We checked whether VHH clones in the hit-cluster other than the examined clone have binding affinity to the antigen. Using the Ig-7 cluster as a representative example, 14 clones that were included in this cluster and located at various locations in the phylogenetic tree were generated, and their antigen binding ability was evaluated by SPR (**Supplementary Figure S4**, **Supplementary Table S3**). All clones showed binding to the antigen, although the binding affinities were different.

### Prediction of hit-clusters using sequence data from alpacas immunized with IgG fragments and EGFR

**Figure 5:**
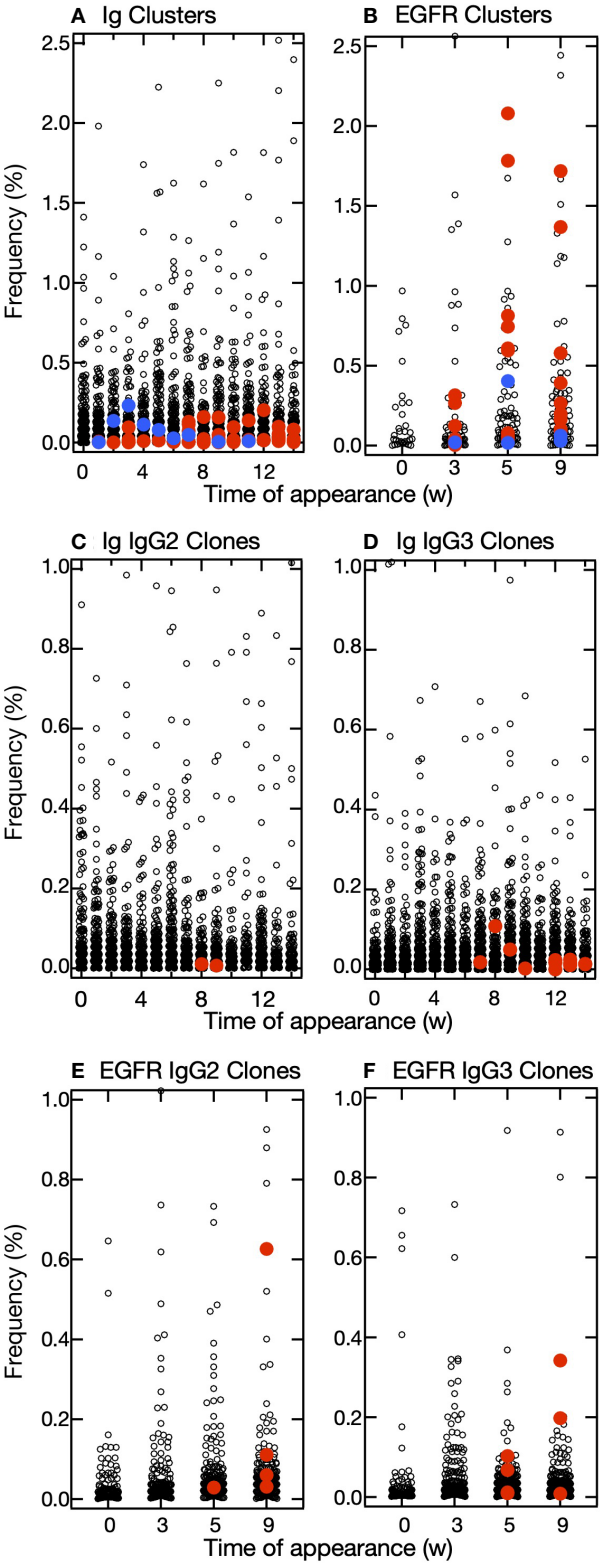
Distribution of frequencies of appearance of clusters and clones. **(A, B)** Plots of the relative frequency of each cluster at the time of appearance and the week in which it appeared in the IgG fragments experiment **(A)** and epidermal growth factor receptor (EGFR) experiment **(B)**. Among the predicted clusters, red circles indicate hit-clusters and blue circles indicate miss-clusters. **(C–F)** Plots of the relative frequency of each clone at the time of appearance and the week in which it appeared in the IgG fragments experiment **(C, D)** and EGFR experiment **(E, F)**. The frequencies of appearance for each clone are drawn separately for IgG2 **(C, E)** and IgG3 **(D, F)**. Red circles indicate clones in the predicted cluster that reacted with the antigens.

**Figure 6:**
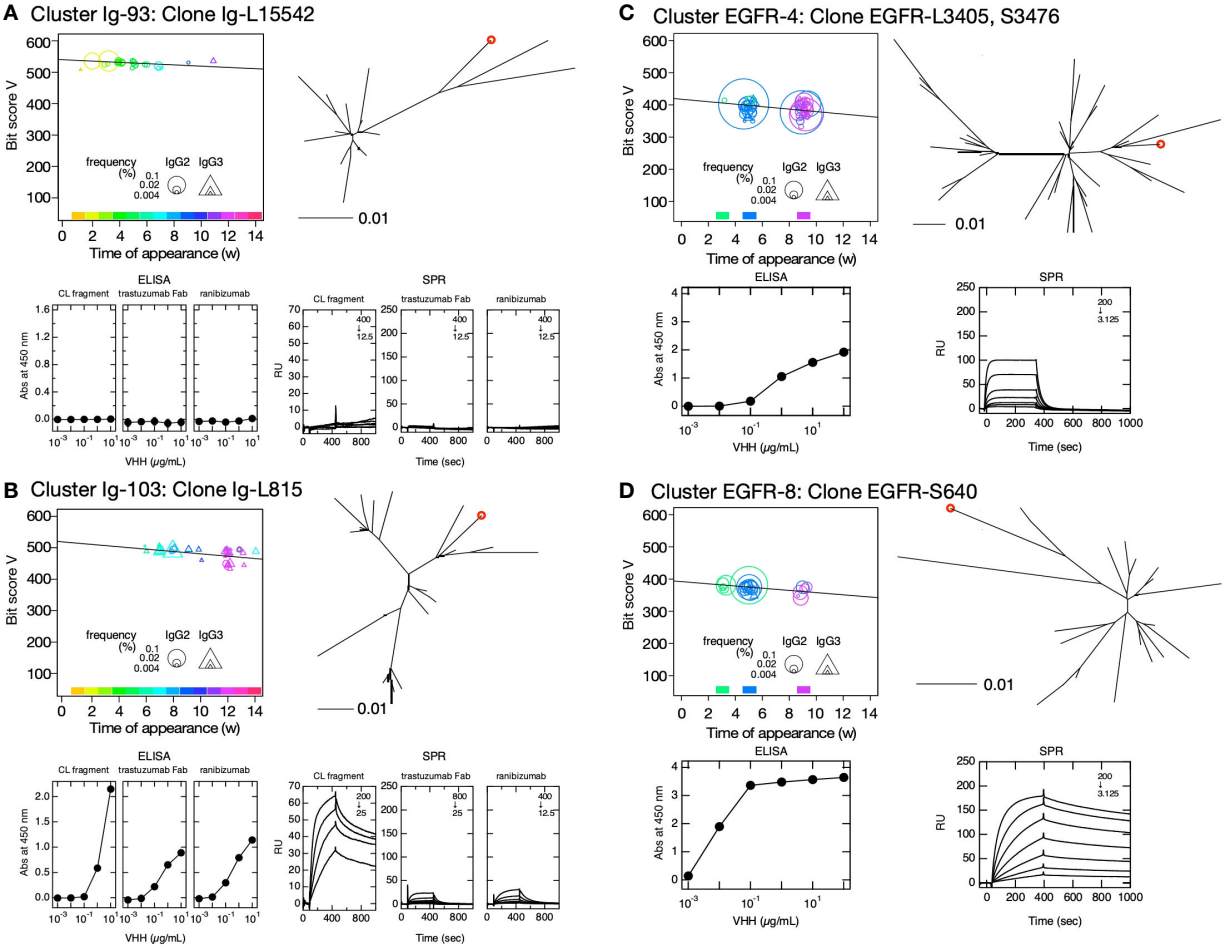
Clusters predicted to contain antigen-bound VHH clones. Clusters Ig-93 **(A)**, Ig-103 **(B)**, EGFR-4 **(C)**, and EGFR-8 **(D)** were selected based on their negative bit score slopes, distinct sequence turnover, and high initial bit scores depicted in the bit score plot (upper left panels). The maximum percentages of appearance of clusters Ig-93, Ig-103, EGFR-4, and EGFR-8 were 0.23, 0.20, 2.7, and 1.8, respectively. The position of the selected VHH clone in the phylogenetic tree is indicated by a red circle (upper right panel). Phylogenetic trees were drawn using DNA sequences including clusters. Bars below the phylogenetic trees indicate distance = 0.01 calculated by JC69 (21) and corresponding to ~1% nucleotide sequence difference. The symbol size in the bit score plot corresponds to weekly clone frequency in IgG2 and IgG3 sequences. Affinities of VHH clone for immobilized human κ C_L_ (left), F_ab_ of trastuzumab (middle) and ranibizumab (right) **(A, B)**, and immobilized human epidermal growth factor receptor are depicted by ELISA (lower left panel) and surface plasmon resonance (SPR) (lower right panel) **(C, D)**. Values inside the SPR panels indicate concentration ranges of VHH clones measured as analytes (in units of nM), and the dilution series of the analytes was 1/2. If there is no scale value on the vertical axis, it is the same as the values on the next panel to the left.

We sought clusters having features of the hit-clusters and selected clusters Ig-93, Ig-103, Ig-126, Ig-139, Ig-143, Ig-175, Ig-245, and Ig-275 with low maximum percentage appearance (0.03%-0.2%) (**Figure 5A**) to match the following criteria (1): the slopes’ bit score plot of candidates should be negative (2), their initial bit scores should be more than >380 (average of the initial bit scores of hit- and miss-clusters), and (3) newly appearing sequences should predominate on a weekly basis, and there should be sequence turnover (**Figures 6A, B**, **Supplementary Figure S5**) (21). The sequences at the tips of the phylogenetic trees constructed for these clusters were prepared as antibody proteins (**Supplementary Table S2**). Antigen binding was tested by using ELISA and SPR. The VHH clones of clusters 103, 126, 139, 143, 175, 245, and 275 bound the antigen. The ELISA and SPR results indicated no interaction between the antigen and the clones from cluster Ig-93. Without experimental screening of antibodies from alpacas immunized with IgG fragments, we were able to identify clusters containing antigen-binding clones, which appeared with low frequencies of occurrence, with a hit rate of >80%.

We then attempted to predict the clusters responding to the antigen from VHH sequences obtained from leukocytes of other alpaca immunized with human EGFR. Sequence clustering and analysis were performed as in the case of immunization with IgG fragments. A total of 70 clusters and 1,678 sequences were identified in this experiment. Among them, we selected clusters EGFR-4, EGFR-8, EGFR-9, EGFR-11, EGFR-14, EGFR-19, EGFR-20, EGFR-23, EGFR-24, EGFR-25, EGFR-34, and EGFR-46 (**Figures 6C, D**, **Supplementary Figure S6**) with a wide range of maximum percentage appearance from 0.3% to 2.7% (**Figure 5B**) using the same criteria as that for cluster predictions against IgG fragments. Of the 12 clusters predicted, 10 clusters contained antigen-binding clones, five of which showed antigen binding by both ELISA and SPR. The numbering of clusters and sequence IDs in the EGFR experiment is the same as in the IgG experiment.

The maximum percentage appearance of VHH clones that are predicted and bind to the antigen was in the range 0.01%–0.11% in IgG fragments and 0.01%–0.63% in EGFR experiments (**Figures 5C–F**). Notably, it was also possible to identify clones with very low frequencies of occurrence, suggesting that our predictive method is useful in detecting clones that appear only at low frequencies of occurrence.

Similar to the analysis of clusters containing the top 16 and experimentally identified clones in the IgG fragment immunization experiments (**Supplementary Figure S2**), some clones in the predicted clusters also showed differences in reactivity with the antigen in ELISA and SPR measurements. These were attributed to variations in antigen structure due to differences in antigen immobilization methods. Clones Ig-L1643 (cluster Ig-126), Ig-L9713 (cluster Ig-139), Ig-L12393 (cluster Ig-175), and Ig-13316 (cluster Ig-245) (**Supplementary Figure S4**) may recognize the C_L_ and C_H1_ interface in the same way as clones Ig-L38 (cluster Ig-5) and Ig-L15235 (cluster Ig-210) (**Supplementary Figure S2**). Clone Ig-L815 (cluster Ig-103) showed reactivity to all three antigens in ELISA and SPR experiments, but the SPR signals for ranibizumab and F_ab_ from trastuzumab were very small (**Figure 6B**). It may possible that the epitope exists on the C_L_ and C_H1_ interface, and the region around the epitope may be fluctuated and partially exposed in the native structure of F_ab_. Clone Ig-6897 (cluster Ig-143) exhibited a signal against ranibizumab only in ELISA experiments, suggesting that the epitope could be buried complementarity-determining regions (CDRs) of light and/or heavy chains. Although a distinct sensorgram of clone EGFR-S36 (cluster EGFR-9) against EGFR in the SPR experiment was observed, the interaction seemed very weak in the ELISA experiment (**Supplementary Figure S6**). Clone EGFR-S36 may recognize multiple amino acids that are far apart in primary sequence but close together in tertiary structure. The antigen binding of clones EGFR-L39 (cluster EGFR-14), EGFR-L194 (cluster EGFR-19), EGFR-L109 (cluster EGFR-23), EGFR-S3849 (cluster EGFR-24), and EGFR-L67 (cluster EGFR-25) was exclusively observed in the ELISA experiments, suggesting that these epitopes may be buried in the native structure of EGFR.

The deviation of SPR data from the 1:1 binding model was especially significant in experiments in which the C_L_ fragment was used as the ligand, such as those observed in clones Ig-L38 (cluster Ig-5), Ig-L15235 (cluster Ig-210), and Ig-L1643 (cluster Ig-126) (**Supplementary Figures S2, S5**). We consider that the cause could be the multiple conformation of antigen around the epitope induced by chemical linkage against sensor-chip, as the C_L_ fragment is smaller than the other antigens and thus could be relatively susceptible to denaturation effect induced by chemical modification for immobilization. Clones that deviated from the fit to the 1:1 binding model were not suitable for quantitative analysis, but the analyte concentration-dependent SPR signal is evidence of antigen binding of these clones. In the ELISA experiments for Ig-L38 (cluster Ig-5), the curve shape was biphasic and deviations from the sigmoid curve shape were observed. Though the exact cause is unclear, it is plausible that the F_ab_ adsorbed onto the plastic surface exhibits structural diversity, leading to multiple conformational states with varying affinities for the antibody. In any case, data from this clone is not suitable for quantitative analysis. However, taking into account the SPR data, we consider that Ig-L38 binds to the antigen.

## Discussion

With certain exceptions, the repertoire development history of the sequences in the immune responsive antibody clusters exhibited a distinct time-dependent pattern in the top 16 abundant cluster at the experiment of IgG fragments immunization. The sequences continuously developed and accumulated diversity throughout immunizations. Furthermore, the sequences showed intensive turnover and the older sequences in the hit-clusters became extinct and were superseded by newly emerged sequences. Using these hit-cluster features, we showed that it is possible to identify VHH clusters containing the antibodies that react to immunized antigens from the sequence information of both IgG fragments and EGFR-immunized alpacas.

The bit score plot is an excellent tool for identifying hit-clusters. It contains the frequency of appearance, the timing of the first appearance, and the bit score of each sequence within the same cluster. Typical patterns were observed for clusters Ig-7, Ig-15, and Ig-16 which had high maximum bit scores. Thus, they started from the sequence nearest that of the germ line. Over time, the sequence generation alternated and the bit score decreased. Hence, affinity maturation progressed. We selected eight and 12 clusters based on the bit score slopes, sequence turnover, and initial bit scores from sequence data of alpacas immunized with IgG fragments and EGFR, respectively. Seven (IgG fragments) and 10 (EGFR) predicted clusters included VHH clones that bound the immunized antigen and were designated as hit-clusters. The overall ratio of hit-clusters to all clusters is unknown. In the analysis of the data of IgG fragments experiment, the ratio for the top 16 clusters except cluster Ig-4 was 6:15. The immune response increased the antigen-responding antibody expression. Consequently, the hit-cluster ratio may increase with cluster appearance frequency. For these reasons, >80% of successful prediction of hit-clusters with relatively low frequencies of occurrence were significant in hit-cluster prediction.

Compared to existing *in silico* approach for selecting antigen-responding antibodies based on their frequency of appearance (4–6), our prediction method is superior in that it allows us to list even infrequent antibodies as candidates. There are attempts to identify the immune-responsive antibody and/or clusters from the sequence data from vaccinated or infected humans. Although these works gave us important information about the development of humoral immunity, a low hit rate of less than 25% (22, 23) compared to our method (>80%) will require a further tune-up of the criteria for the selection of immune-responsive antibody and/or clusters. A combination of bio-panning and NGS analysis successfully identified binding VHH clones not detected by conventional bio-panning alone (13, 17, 24, 25). Unlike these studies, the prediction method proposed here requires no bio-panning. In addition, conventional methods of acquiring antibodies using immunized animals, such as hybridoma technology or screening using a phase display or cell sorter, require antigen–antibody reaction information for the identification of antigen-responding antibodies. On the other hand, certain *in silico* methods, including our approach, necessitate initial antigen immunization in animals; however, they distinguish themselves by not requiring antigens for subsequent antibody identification.

We examined a total of 39 independent VHH clusters, where cluster Ig-4 was excluded due to abnormal SPR data (**Supplementary Table S2**). The number of clusters including the antigen-responding antibody and miss-clusters was 27 and 12, respectively. In hit- and empirically identified clusters, usage of *IGHV3S53* and *IGHJ4* was the highest, and these frequencies were 59% and 63%, respectively. Similarly in miss-cluster, *IGHV3S53* and *IGHJ4* had the highest usage at 42% and 33%, respectively. For the frequently used IGHV3S53 and IGHJ4, there was a difference in usage between hit- and miss-clusters; however, we do not consider that the difference is large enough to be used for prediction. The V genes present only in miss-cluster were *IGHV3-1*, *IGHV3S1*, and *IGHV3S31*, which appeared at once. *IGHV3S65* was observed three times in hit-clusters in the EGFR experiment. Although the sample size is small in this paper only, with the accumulation of future data, information on these relatively low-usage V and J genes may be useful for predicting hit-cluster. The mean values of the lengths of CDR-H3 of hit- and miss-clusters were 14.5 ± 5.8 and 15.5 ± 2.8, respectively, showing no significant differences. For somatic hypermutation levels, bit score is the relevant parameter. For all clusters examined, the range of bit score over the entire immunization period was 440 ± 50-370 ± 56 for the hit-cluster and 371 ± 67–330 ± 75 for the miss cluster. For clusters Ig-1 to Ig-16 (excluding Ig-4), selected without using the prediction criteria, the range of bit score over the entire immunization period was 439 ± 58–336 ± 64 for the hit-cluster and 338 ± 37–294 ± 37 for the miss-cluster. In both cases, more mutations were observed in the miss clusters. A rough estimation of the number of mutations in the V gene region based on the difference in bit scores shows that the miss-cluster has about 5–10 more mutations on average. This suggests that some of the miss-clusters were already sensitized by other antigens prior to immunization and were a collection of antibodies that had already underwent affinity maturation. On the other hand, looking only at the bit score at the end of the experiment (week 14), some of the hit- and miss-clusters exhibited bit scores of around 400. Therefore, predicting hit-clusters at a specific time point after a titer increase against the antigen, using bit scores or the degree of mutation as an indicator, could be challenging.

Based on the foregoing results, we demonstrated the feasibility of the method to predict immune-responsive VHH clusters and sequences including those with low frequencies of appearance based on their bit score plots. It remains to be determined whether the discoveries herein are applicable to conventional antibody and immune systems in other animals. Therefore, future research should use other animal species to validate our prediction method proposed in this manuscript.

## Data availability statement

The datasets presented in this study can be found in online repositories. The name of the repository and accession number can be found below: https://www.ddbj.nig.ac.jp/, PRJDB11899.

## Ethics statement

The animal study was approved by the Committee for the Experiments involving Animals of the National Institute of Advanced Industrial Science and Technology (permit number: 2013-149) and Animal Care and Use Committee of Ark Resource Co., Ltd. (permit number: AW-130012). The study was conducted in accordance with the local legislation and institutional requirements.

## Author contributions

TM: Conceptualization, Formal analysis, Investigation, Methodology, Project administration, Visualization, Writing – original draft, Writing – review & editing. YA-O: Conceptualization, Funding acquisition, Investigation, Methodology, Project administration, Visualization, Writing – original draft, Writing – review & editing. L-KK: Investigation, Writing – review & editing. NK: Investigation, Visualization, Writing – original draft, Writing – review & editing. NM: Conceptualization, Funding acquisition, Investigation, Visualization, Writing – original draft, Writing – review & editing. YM: Conceptualization, Investigation, Writing – review & editing. TF: Conceptualization, Investigation, Writing – review & editing. YI: Funding acquisition, Investigation, Writing – review & editing. YH: Conceptualization, Funding acquisition, Investigation, Methodology, Project administration, Visualization, Writing – original draft, Writing – review & editing.
