# Supplementary material for: Prediction of antigen-responding VHH antibodies by tracking the evolution of antibody along the time course of immunization
Source: Front Immunol. 2024 Jan 16;14:1335462. doi: 10.3389/fimmu.2023.1335462 (PMC10825579; doi:10.3389/fimmu.2023.1335462)
Supplement: Supplementary file 1 [file DataSheet_1.pdf]

Supplementary Material

Prediction of antigen-responding VHH antibodies by tracking evolution of antibody along time course of immunization

Tomonari Matsuda, Yoko Akazawa-Ogawa, Lilian-Kaede Komaba, Norihiko Kiyose, Nobuo Miyazaki, Yusaku Mizuguchi, Tetsuo Fukuta, Yuji Ito, & Yoshihisa Hagihara
Yoshihisa Hagihara

Email: hagihara-kappael@aist.go.jp

This PDF file includes:
Supplementary Figures S1 to S6
Supplementary Tables S1 to S3

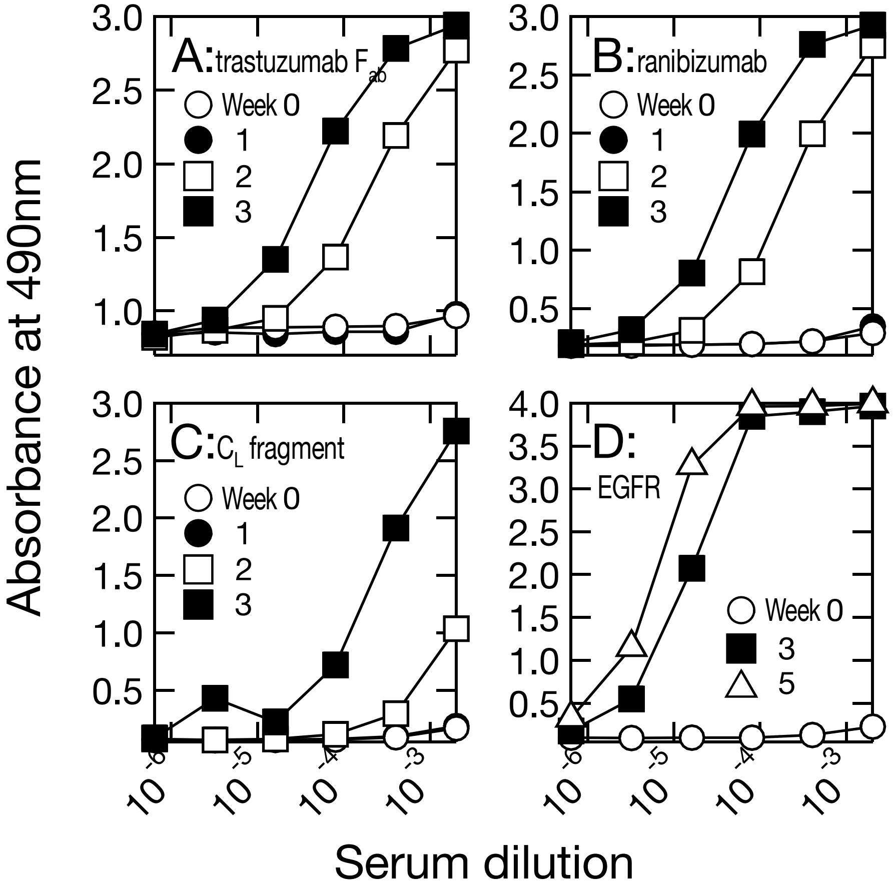

SUPPLEMENTARY FIGURE S1 | Serum titer of immunized alpacas. **(A, B & C)** Serum Titer of alpaca immunized by IgG fragments at weeks 0, 1, 2 and 3 were measured against F_{ab} of trastuzumab, ranibizumab and human κ C_L fragment. **(D)** Serum Titer of alpaca immunized by human EGFR at weeks 0, 3 and 5 were measured against human EGFR. In all experiments, the reactions between antibody and injected antigens were not observed at week 0, indicating alpaca bloods used in the experiments were not reactive to the used antigen prior to inoculation.

Suppl. Figure S1

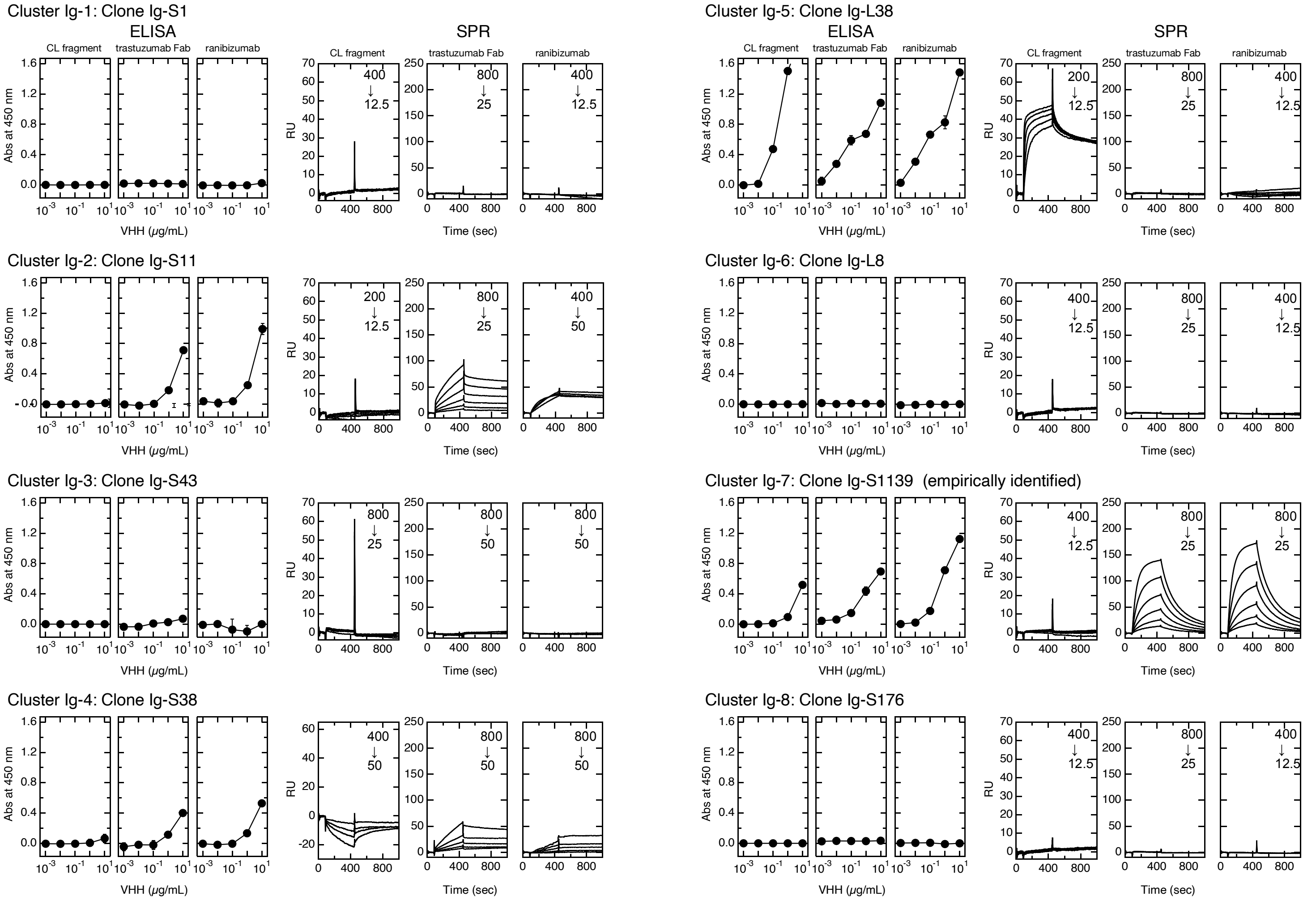

Suppl. Figure S2

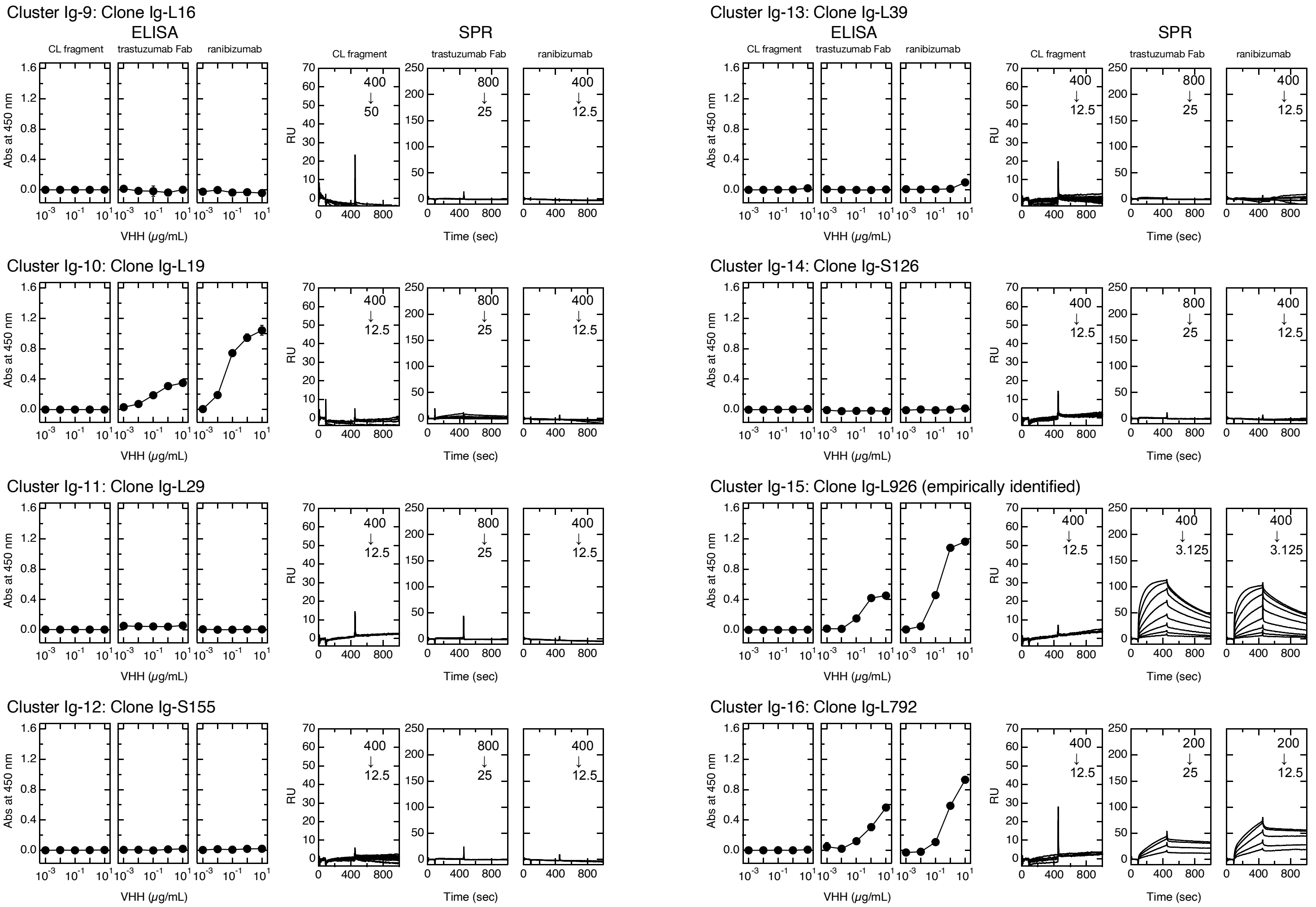

Suppl. Figure S2

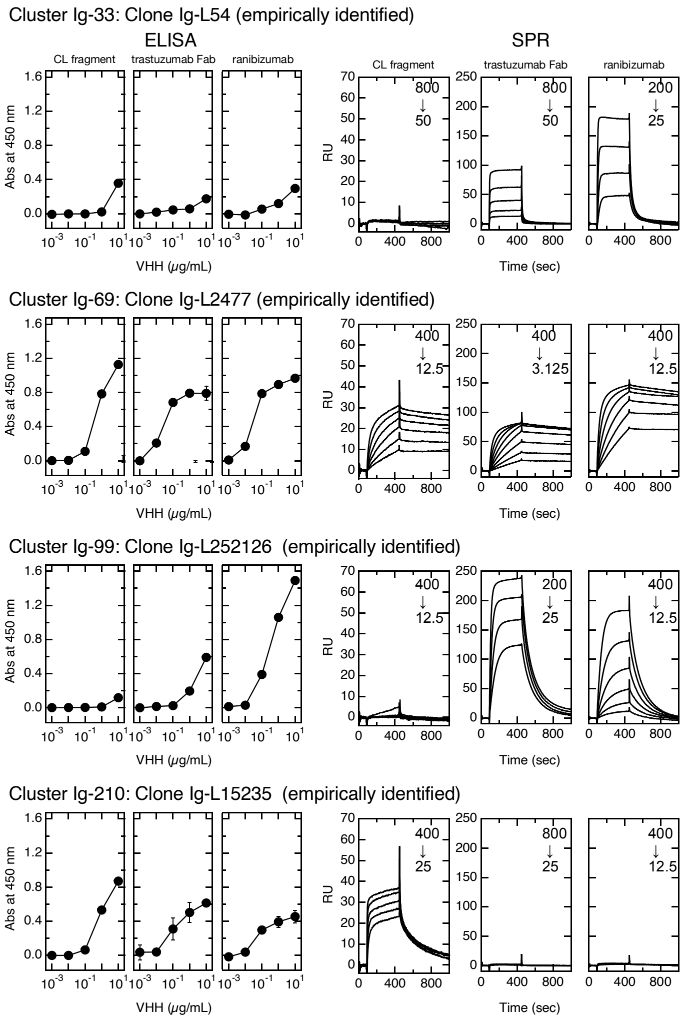

SUPPLEMENTARY FIGURE S2 | Antigen-binding affinities of empirically identified clones and those in top 16 clusters from alpaca immunized by IgG fragments. Cluster number indicates order of maximum percentage appearance. The percentage of appearance is the sum of the percentage occupancy of IgG2 and IgG3 sequences in each cluster relative to all IgG2 and IgG3 sequences in each week. Maximum percentage of appearance is the highest percentage of appearance of a cluster during immunization. Clusters Ig-7, 15, 33, 69, 99 and 210 included empirically identified antigen-binding sequences. Antigen-binding affinities of clones were evaluated by ELISA (left three panels) and SPR (right three panels) vs. immobilized human κ CL (left), Fab of trastuzumab (middle) and ranibizumab (right). Values inside SPR panels indicate concentration ranges of VHH clones measured as analytes in units of nM and the dilution series of the analytes was 1/2. If there is no scale value on the vertical axis, it is the same as the values on the next left panel.

Suppl. Figure S2

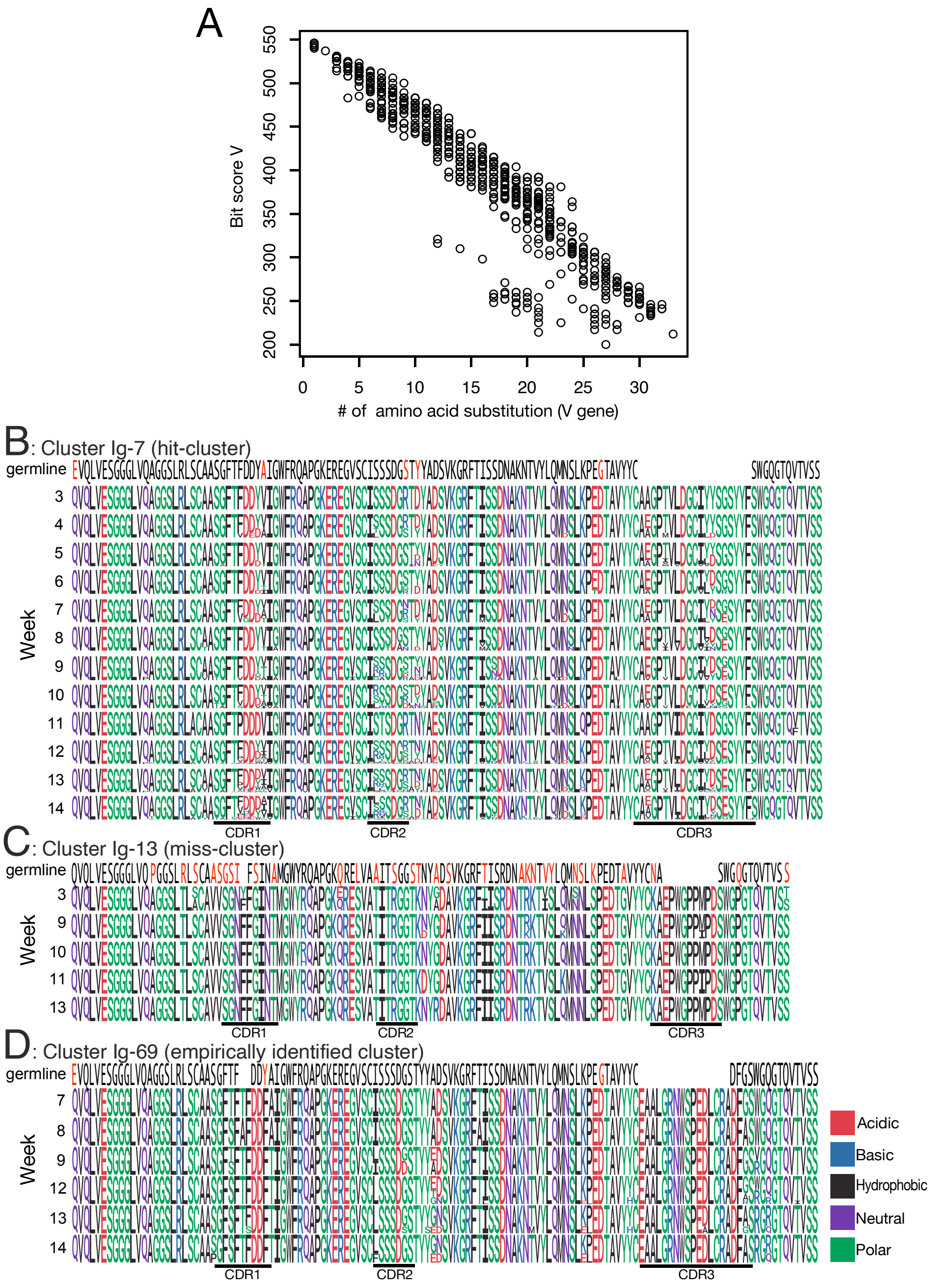

SUPPLEMENTARY FIGURE S3 | (A) Relationship between the difference from the germline V gene reference and the bit score used for the bit score plot in IgG fragments immunization experiments. **(B-D)** Sequence logo plots of amino acid sequences clones in the clusters Ig-7 **(hit-cluster: B)**, Ig-13 **(miss-cluster: C)** and Ig-69 **(empirically identified cluster: D)**. The plots were done by the timing of the first appearance of the clones. For example, in the "week 3," the clone of the sequence first appeared at week 4. The red letters in the germline series are sequences that differ from the sequence of the youngest week. The regions of CDRs defined by IMGT numbering scheme were underlined.

Suppl. Figure S3

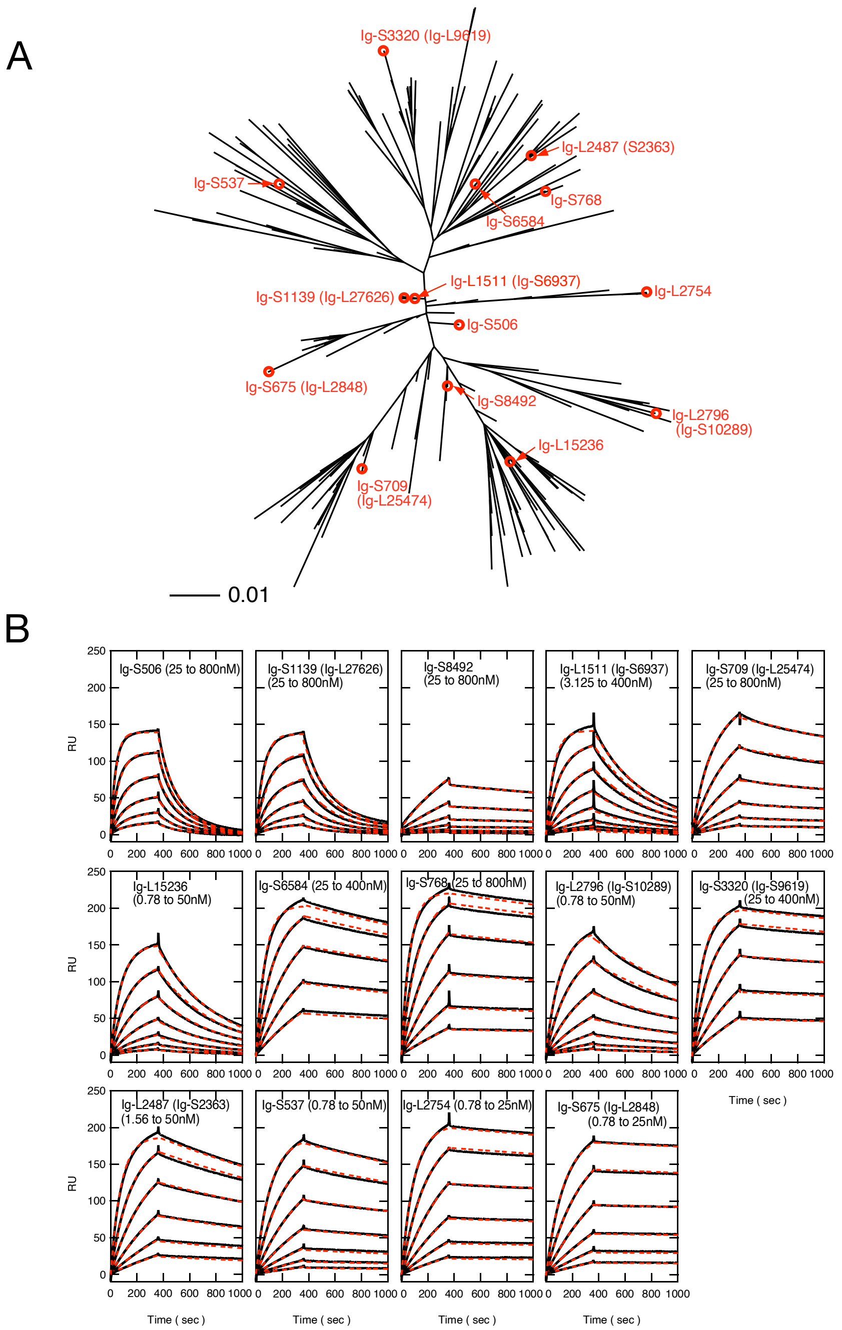

SUPPLEMENTARY FIGURE S4 | Antigen binding activity of multiple clones in the same cluster. The Ig-7 cluster was chosen as a typical cluster, and VHH clones were generated as indicated in red in the phylogenetic tree. The affinity of each clone was measured by SPR against immobilized F_{ab} of trastuzumab. Values inside SPR panels indicate concentration ranges of VHH clones measured as analytes in units of nM and the dilution series of the analytes was 1/2. The "1:1 binding model" was used for determining K_D. All the clones bound to trastuzumab F_{ab}, however the affinities differed significantly.

Suppl. Figure S4

Cluster Ig-126: Clone Ig-L1643

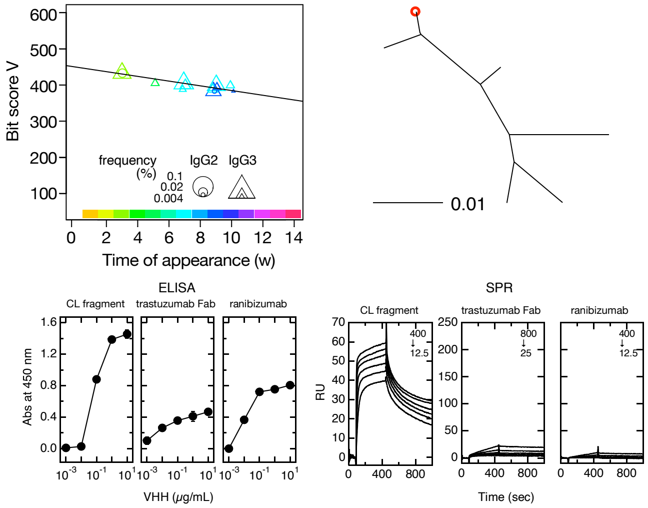

Cluster Ig-139: Clone Ig-L9713

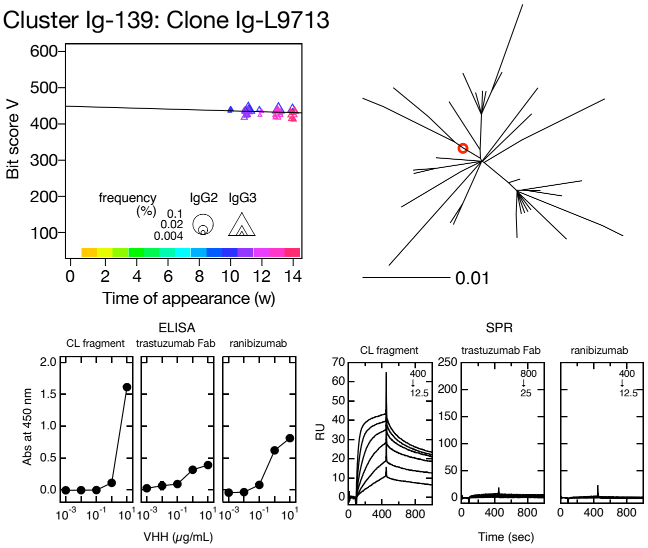

Suppl. Figure S5

Cluster Ig-143: Clone Ig-L6897

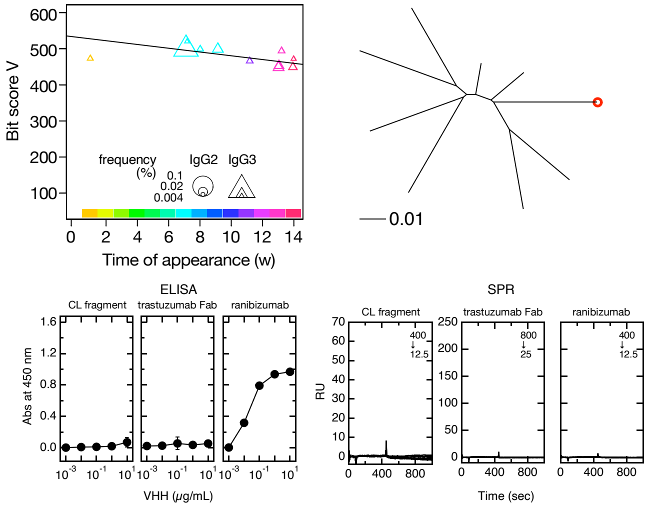

Cluster Ig-175: Clone Ig-L12393

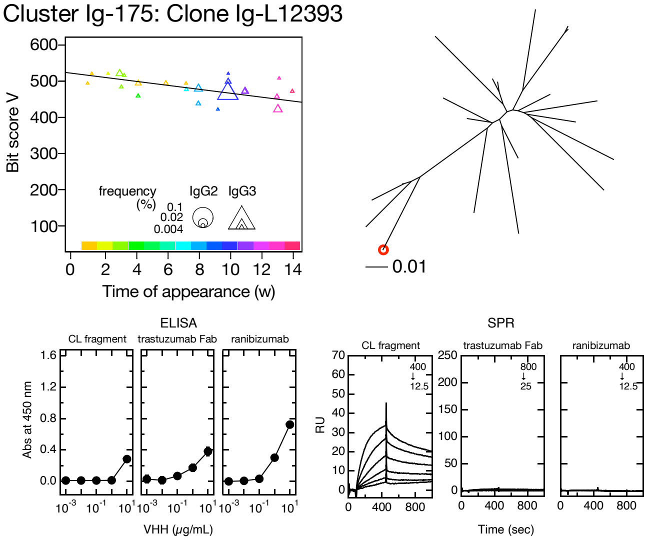

Suppl. Figure S5

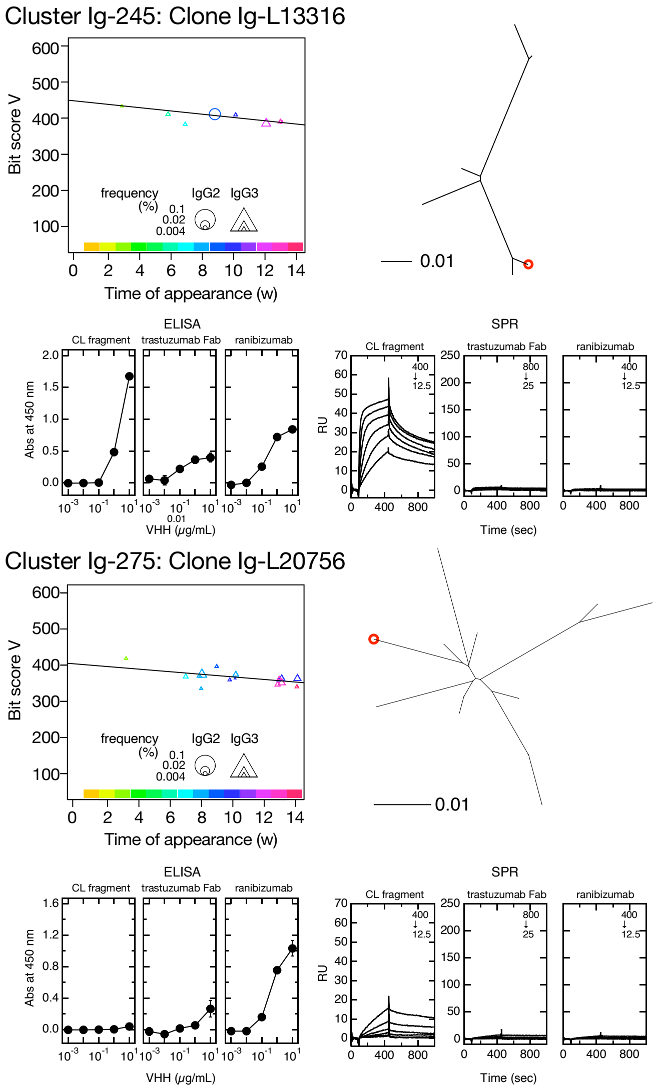

SUPPLEMENTARY FIGURE S5 | Clusters predicted to contain IgG fragment-bound VHH clones. Clusters were selected based on negative bit score slope, distinct sequence turnover, and high initial bit score as depicted by bit score plot (upper left panels). Maximum percentage appearance of clusters were 0.16 (cluster Ig-126), 0.14 (cluster Ig-139), 0.13 (cluster Ig-143), 0.10 (cluster Ig-175), 0.05 (cluster Ig-245) and 0.03 (cluster Ig-275). Position of selected VHH clone in phylogenetic tree is indicated by red circle (upper right panel). Symbol size in bit score plot indicates weekly clone frequency in IgG2 and IgG3 sequences. Antigen-binding affinity of VHH clone vs. immobilized human κ C_L (left), F_{ab} of trastuzumab (middle) and ranibizumab (right) are shown by ELISA (lower left three panels) and SPR (lower right three panels). Values inside SPR panels indicate concentration ranges of VHH clones measured as analytes in units of nM and the dilution series of the analytes was 1/2. If there is no scale value on the vertical axis, it is the same as the values on the next left panel.

Suppl. Figure S5

Cluster EGFR-9: Clone EGFR-S36

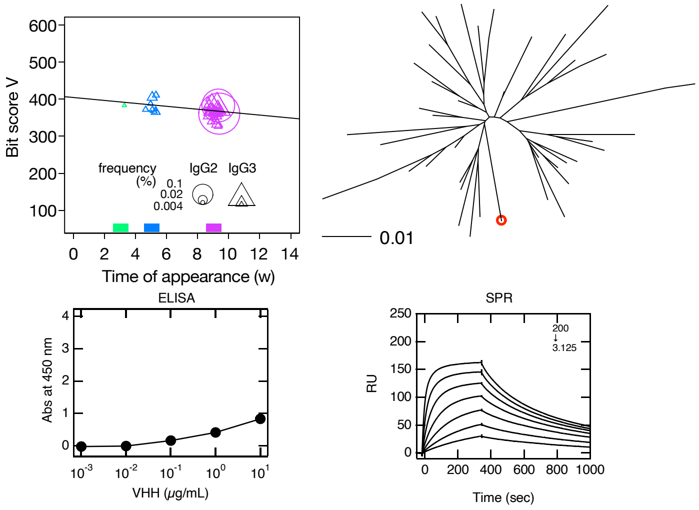

Cluster EGFR-11: Clone EGFR-L7, S1361

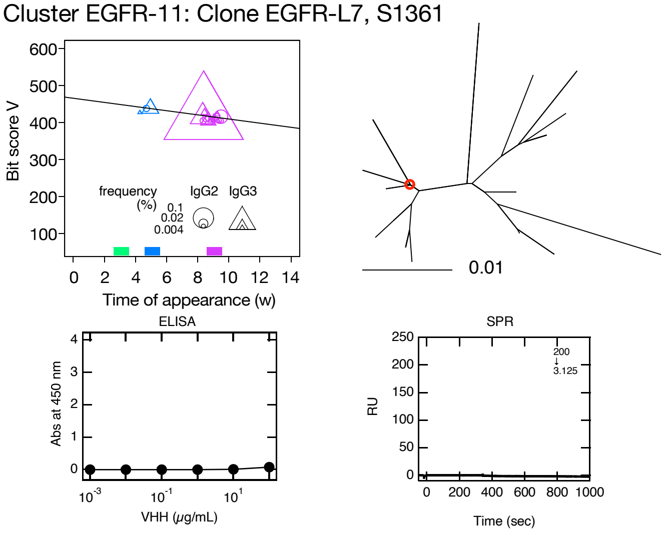

Suppl. Figure S6

Cluster EGFR-14: Clone EGFR-L39

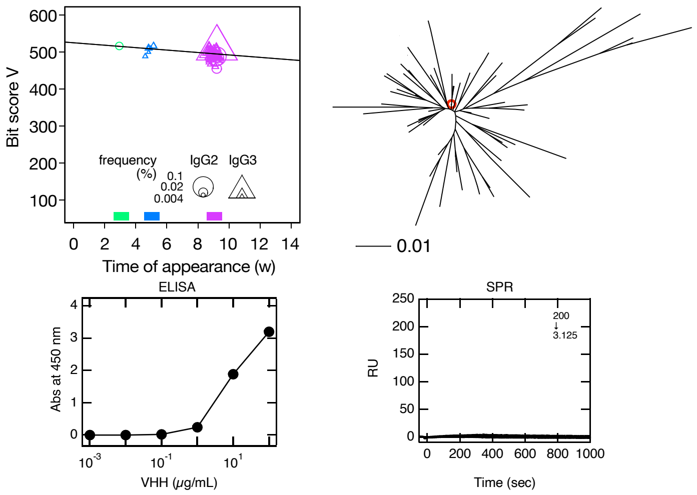

Cluster EGFR-19: Clone EGFR-L194

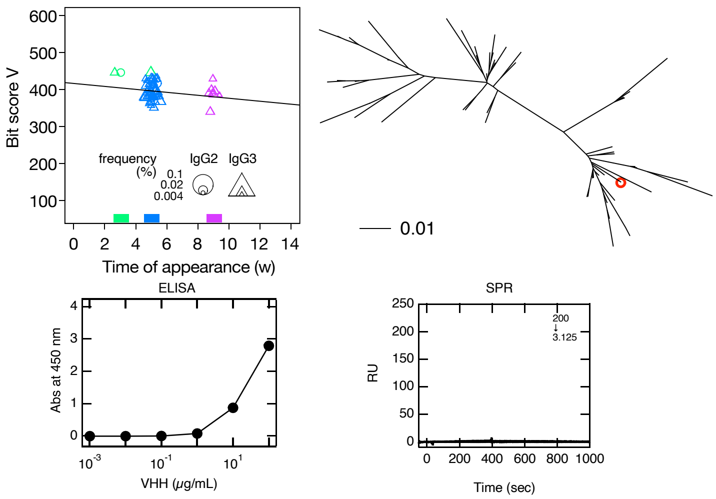

Suppl. Figure S6

Cluster EGFR-20: Clone EGFR-L4879

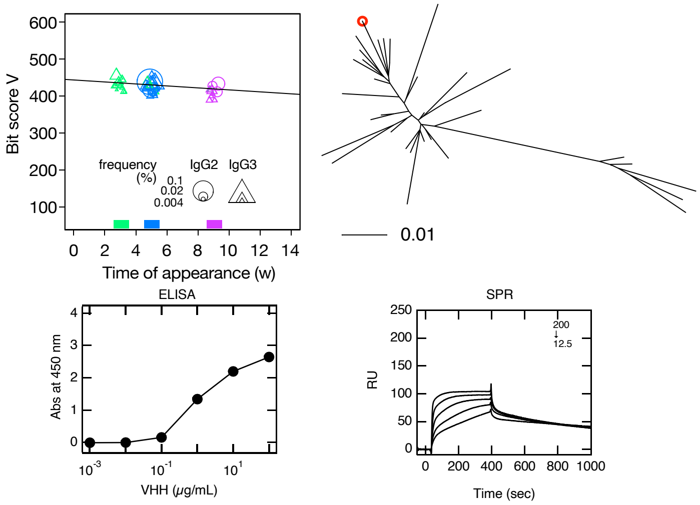

Cluster EGFR-23: Clone EGFR-L109

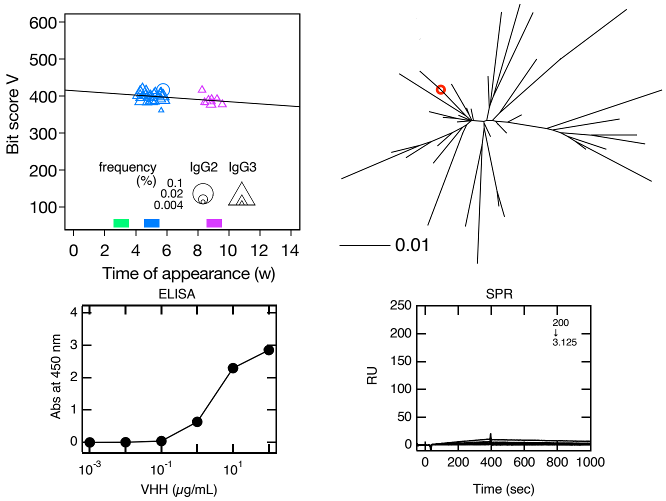

Suppl. Figure S6

Cluster EGFR-24: Clone EGFR-S3849

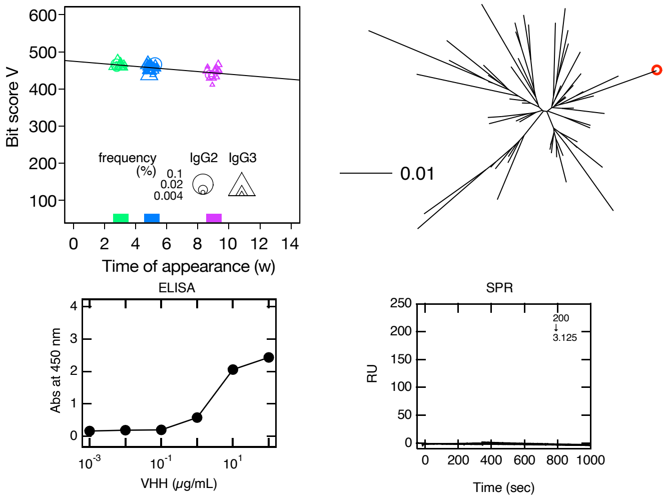

Cluster EGFR-25: Clone EGFR-L67

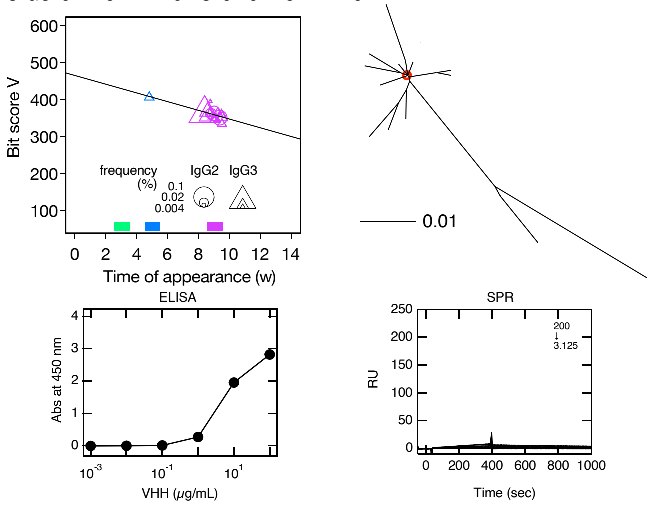

Suppl. Figure S6

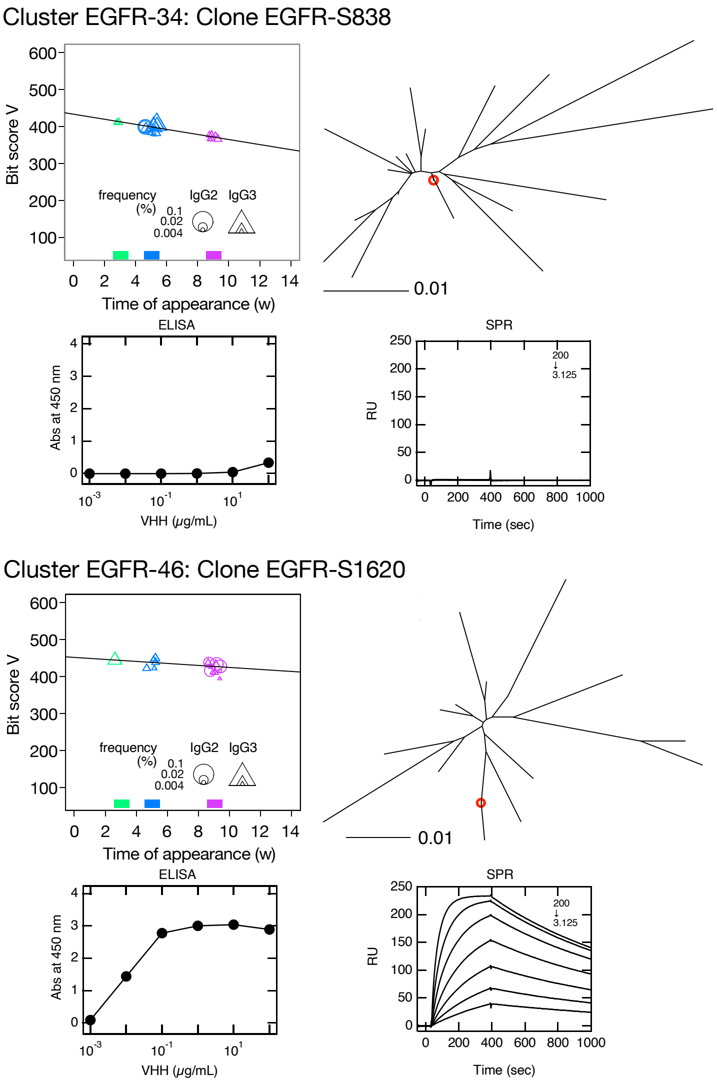

SUPPLEMENTARY FIGURE S6 | Clusters predicted to contain EGFR-bound VHH clones. Clusters were selected using the same criteria as for IgG fragments experiment. Maximum percentage appearance of clusters were 1.7 (cluster EGFR-9), 1.7 (cluster EGFR-11), 1.4 (cluster EGFR-14), 0.81 (cluster EGFR-19), 0.74 (cluster EGFR-20), 0.61 (cluster EGFR-23) , 0.60 (cluster EGFR-24) , 0.58 (cluster EGFR-25) , 0.39 (cluster EGFR-34) and 0.27 (cluster EGFR-46). Position of selected VHH clone in phylogenetic tree is indicated by red circle (upper right panel). Symbol size in bit score plot indicates weekly clone frequency in IgG2 and IgG3 sequences. Antigen-binding affinity of VHH clone vs. immobilized human EGFR are shown by ELISA (lower left panels) and SPR (lower right panels). Values inside SPR panels indicate concentration ranges of VHH clones measured as analytes in units of nM and the dilution series of the analytes was 1/2.

Suppl. Figure S6

SUPPLEMENTARY TABLE S1 | V and J gene usage before and after immunization

V Gene	J Gene	week 0 (%)	week 1 (%)	week 2 (%)	week 3 (%)	week 4 (%)	week 5 (%)	week 6 (%)	week 7 (%)	week 8 (%)	week 9 (%)	week 10 (%	week 11 (%	week 12 (%	week 13 (%	week 14 (%)
IGHV3S53	IGHJ4	15.3	19.3	21.3	13.6	19.2	17.8	22.1	18.6	20.5	17.7	19.7	23.7	21.8	21.2	21.3
IGHV3S66	IGHJ4	14.8	15.8	14.7	21.9	15.4	11.8	11.9	13.3	14	12.6	11.5	14.8	13.2	14.6	13.9
IGHV3S61	IGHJ4	13.2	10.3	9.3	6.4	10.3	12.8	10	10.1	9.9	9.5	12.8	8.7	12.8	12.9	10.3
IGHV3S66	IGHJ6	6.1	4.9	5.1	3	6.7	4.7	7.6	7.3	5.2	5.2	6.9	6.3	7.6	6.8	5.2
IGHV3S53	IGHJ6	6.1	5.2	5.6	6.4	5.7	7	6.6	5.4	4.9	7.9	5.5	4.7	4.2	4.1	4.4
IGHV3S61	IGHJ6	5.8	7.1	6.6	6.9	7	10.3	5.9	8.7	7.7	7.5	5.8	5.8	7.4	4.7	6.2
IGHV3S66	IGHJ7	5.4	5	5.4	3.6	5.4	4.4	4.6	3.9	7.8	4.5	4.7	5.1	3.7	4.8	4.5
IGHV3S61	IGHJ7	4	5.2	4.2	5.1	4.8	2.9	3.2	3.5	4.2	4.8	4.5	5.2	4.1	3.9	4.6
IGHV3S62	IGHJ4	2.8	2.1	2.1	1.6	1.5	1.5	1.5	1.1	1.3	0.9	3.3	1.2	1.1	0.9	1.5
IGHV3S65	IGHJ4	2.8	1.9	1.7	1.6	1.5	3.2	2.9	2.4	1.9	2.5	2	1.3	1.7	2	1.2
IGHV3S63	IGHJ4	2.8	3	2.5	2.1	2.4	2	1.8	2.7	3.6	2	2.2	2.1	2.3	3.1	2
IGHV3S41	IGHJ4	2.3	1.9	4.2	1	1.6	1.7	2.6	5.2	2.7	4.4	4.9	3.7	2.3	3.6	3.2
IGHV3S1	IGHJ6	1.8	2	1.7	0.9	0.8	1	1.2	1	0.7	1	0.5	0.8	0.8	0.9	0.7
IGHV3S1	IGHJ4	1.3	1.3	1.4	1.1	1.2	1.1	2.2	1.2	1	1	1	2.8	2	1.5	1.4
IGHV3S53	IGHJ7	1.2	0.9	1	15.7	0.7	1.1	1.2	0.9	0.8	0.6	1	1.2	1.1	1.7	1.3
IGHV3S62	IGHJ6	1.1	1.1	0.9	0.7	1.1	0.9	1.2	0.4	0.8	0.5	0.6	0.8	0.6	0.6	0.9
IGHV3S41	IGHJ6	1.1	1.5	1.1	0.7	4	4.1	2.7	3.5	1.5	6.1	4.9	3.8	3.1	3.1	3.9
IGHV3S65	IGHJ6	1	0.6	0.5	0.4	0.4	0.3	0.5	0.2	0.3	1	0.6	0.3	0.4	0.4	0.7
IGHV3S32	IGHJ4	1	0.3	0.4	0.4	0.2	0.3	0.5	0.2	0.4	0.3	0.6	0.6	0.3	0.2	0.8
IGHV3S63	IGHJ6	0.9	1.5	0.9	0.8	0.9	1.8	1.1	1.4	1.4	1	0.6	0.5	1.8	0.6	0.5
IGHV3S62	IGHJ2	0.9	0.5	0.2	0.2	1.2	2.1	0.2	0.7	0.1	0.8	0	0	0.2	0	0.1
IGHV3S66	IGHJ2	0.6	0.2	0.5	0.3	0.6	0.4	1.3	0.5	0.5	0.3	0.4	0.4	0.5	0.3	2.3
IGHV3S62	IGHJ7	0.6	0.8	0.7	0.4	0.2	0.4	0.6	0.3	0.8	0.6	0.4	0.2	0.3	0.5	0.8
IGHV3S63	IGHJ7	0.5	0.3	0.3	0.3	0.2	0.4	0.3	2.1	0.3	0.6	0.2	0.2	0.2	0.3	0.3
IGHV3S32	IGHJ6	0.4	0.1	0.1	0.1	0.2	0.1	0.1	0.1	0.1	0.1	0.1	0.1	0.1	0.1	0.1
IGHV3S53	IGHJ3	0.3	0.1	0.1	0.1	0.2	0.2	0.2	0.1	0.2	0.3	0.3	0.1	0.1	0.2	0.2
IGHV3S53	IGHJ2	0.3	0.3	0.3	0.2	0.5	0.3	0.7	0.3	0.4	0.4	0.4	0.2	0.2	0.4	0.4
IGHV3S37	IGHJ6	0.3	0.3	0.4	0.1	0.3	0.3	0.2	0.4	0.2	0.2	0.2	0.1	0.5	0.1	0.1
IGHV3S64	IGHJ4	0.3	0.1	0.1	0.1	0.1	0	0.2	0	0	0.1	0	0	0.1	0	0.1
IGHV3S61	IGHJ2	0.3	0.5	0.3	0.2	0.5	0.3	0.3	0.4	0.5	0.4	0.2	0.1	0.3	0.4	0.6
IGHV3S54	IGHJ4	0.3	0.1	0.3	0.2	0.1	0.2	0.1	0.3	0.3	0.1	0.1	0.2	0.1	0.2	0.2
IGHV3S39	IGHJ4	0.2	0.3	0.3	0.2	0.2	0.4	0.4	0.4	0.3	0.3	0.4	0.3	0.3	0.5	1
IGHV3S1	IGHJ7	0.2	0.3	0.6	0.2	0.1	0.1	0	0.1	0.1	0.4	0.4	0.1	0.1	0.1	0.1
IGHV3S65	IGHJ7	0.2	0.3	0.2	0.2	0.4	0.2	0.1	0.1	0.1	0.1	0.1	0.1	0.1	0.1	0.2
IGHV3-1	IGHJ4	0.2	0.5	0.3	0.2	0.3	0.2	0.2	0.2	0.2	0.2	0.2	0.3	0.4	0.6	0.5
IGHV3-2	IGHJ4	0.1	0.4	0.4	0.2	0.1	0.2	0.2	0.1	0.2	0.1	0.1	0.3	0.2	0.3	0.2
IGHV3S63	IGHJ2	0.1	0.1	0.1	0	0	0	0	0.1	0.1	0	0.1	0.2	0	0.1	0.1
IGHV3S40	IGHJ6	0.1	0.1	0.1	0.1	0.2	0.1	0.1	0.2	0.1	0.1	0.1	0.1	0.1	0.2	0.1
IGHV3-2	IGHJ3	0.1	0	0	0	0	0	0	0	0	0	0	0	0	0	0
IGHV3S1	IGHJ2	0.1	0	0	0	0	0	0	0	0	0	0	0	0	0	0
IGHV3S31	IGHJ4	0.1	0.2	0	0	0	0.1	0	0	0.1	0	0	0.1	0	0	0
IGHV3S7	IGHJ4	0.1	0	0	0	0.1	0.1	0	0.1	0	0	0	0	0	0	0
IGHV3S5	IGHJ6	0.1	0	0.2	0	0	0	0	0	0	0	0	0	0	0	0
IGHV3S31	IGHJ6	0.1	0.1	0.1	0.1	0.3	0.1	0.1	0	0.1	0.9	0.1	0.1	0.3	0.1	0
IGHV3S64	IGHJ7	0.1	0	0.1	0	0	0	0	0	0	0	0	0.1	0	0	0
IGHV3S28	IGHJ6	0.1	0.1	0.1	0	0	0	0	0.1	0	0	0	0	0	0.1	0.1
IGHV3-1	IGHJ3	0.1	0	0	0	0	0	0.1	0	0	0	0	0	0	0	0
IGHV3S28	IGHJ7	0.1	0	0	0.1	0	0	0	0	0.1	0	0	0	0.1	0	0
IGHV3S12	IGHJ6	0.1	0.1	0	0	0	0	0.1	0	0.1	0	0	0	0	0	0.1
IGHV3S41	IGHJ7	0.1	0.2	0.3	0.2	0.2	0.1	0	0.1	0.1	0.1	0	0	0.1	0.1	0
IGHV3S66	IGHJ3	0.1	0.1	0.2	0.1	0.2	0.1	0.2	0.1	0.3	0.1	0.2	0.2	0.2	0.1	0.1
IGHV3S28	IGHJ4	0.1	0.1	0.2	0.1	0.3	0.1	0.1	0.1	0.1	0.1	0.1	0.1	0.3	0.2	0.2
IGHV3S44	IGHJ4	0.1	0.1	0.1	0	0	0	0	0	0	0	0	0	0	0	0
IGHV3S39	IGHJ6	0.1	0.2	0.1	0	0.1	0.2	0.2	0.1	0.1	0.1	0.2	0.3	0.9	1.1	1
IGHV3S55	IGHJ4	0.1	0	0	0.1	0.1	0	0.1	0	0.1	0	0	0	0	0	0
IGHV3S25	IGHJ6	0.1	0	0.1	0	0	0	0	0	0	0	0	0	0	0	0
IGHV3S6	IGHJ6	0.1	0	0	0	0	0	0	0	0	0	0	0	0	0	0
IGHV3S54	IGHJ6	0.1	0.1	0.1	0.1	0.1	0.1	0.1	0.1	0.1	0	0	0.1	0	0	0.1
IGHV3S25	IGHJ4	0.1	0	0.1	0	0	0	0.2	0	0	0	0.1	0.1	0	0	0.1
IGHV3S41	NA	0.1	0	0	0	0.1	0	0	0	0	0	0	0	0	0	0
IGHV3S44	IGHJ6	0.1	0	0	0	0	0	0	0	0	0	0	0	0	0.1	0
IGHV3S54	NA	0.1	0.1	0	0	0	0	0	0	0	0	0	0	0	0	0
IGHV3S34	IGHJ6	0.1	0.1	0	0.1	0.2	0	0	0	0.1	0	0	0	0	0	0
IGHV3-1	IGHJ6	0.1	0.1	0.1	0.1	0.2	0.2	0.1	0.1	0.1	0.1	0.1	0.2	0.2	0.3	0.2
IGHV3S40	IGHJ4	0.1	0	0.1	0.1	0	0.1	0.1	0.1	0.1	0.2	0	0.1	0.1	0.2	0.1
IGHV3S66	IGHJ1	0.1	0	0	0	0	0	0	0	0	0	0	0	0	0	0
IGHV3-1	IGHJ7	0	0	0.1	0.2	0.1	0.1	0	0.1	0	0.2	0	0	0.1	0	0
IGHV3S37	IGHJ4	0	0.1	0.1	0.1	0.1	0.1	0.1	0.1	0.1	0.1	0.1	0.3	0.1	0.2	0.4
IGHV3S61	IGHJ3	0	0	0.1	0.1	0	0	0	0	0.1	0	0	0	0	0	0
IGHV3S41	IGHJ3	0	0.1	0.1	0	0.1	0	0	0	0	0	0	0	0	0	0
IGHV3S60	IGHJ6	0	0	0	0	0	0	0.1	0	0	0	0	0	0	0	0
IGHV2S2	IGHJ4	0	0	0	0	0	0	0	0	0	0	0	0	0	0	0
IGHV2S5	IGHJ4	0	0	0	0	0	0	0	0	0	0.1	0.1	0	0	0	0.1
IGHV3S62	IGHJ3	0	0	0.1	0	0	0	0	0	0	0	0	0	0.1	0.1	0.1
IGHV2S5	IGHJ6	0	0	0	0	0	0	0	0	0	0	0	0	0	0	0
IGHV3S64	IGHJ6	0	0	0	0.1	0.1	0.1	0	0	0	0	0	0	0	0	0
IGHV3S12	IGHJ4	0	0.1	0.1	0.2	0.1	0.1	0.1	0.1	0.1	0.1	0	0	0.1	0.1	0
IGHV3S14	IGHJ6	0	0	0	0	0	0	0	0	0	0	0	0	0	0	0
IGHV3S34	IGHJ4	0	0.1	0.1	0	0	0	0	0	0	0	0	0	0	0	0
IGHV3S24	IGHJ2	0	0	0	0	0	0	0	0	0	0	0	0	0	0	0
IGHV3-2	IGHJ6	0	0.1	0.1	0.1	0.1	0.1	0.1	0.1	1.1	0.1	0.1	0.2	0.1	0.1	0

IGHV3S10	IGHJ6	0	0	0	0	0	0	0	0	0	0	0	0	0	0	0
IGHV3S56	IGHJ4	0	0	0	0	0	0	0	0	0	0	0.1	0	0	0	0
IGHV3S60	IGHJ4	0	0	0	0	0	0	0	0	0	0	0	0	0	0	0
IGHV3S10	IGHJ4	0	0	0	0	0	0	0	0	0	0	0	0	0	0	0
IGHV3S53	NA	0	0	0	0	0	0	0	0	0	0	0	0	0	0	0
IGHV3S1	IGHJ3	0	0	0	0	0	0	0	0	0	0	0	0	0	0	0
IGHV3S36	IGHJ6	0	0	0	0	0	0	0	0	0	0	0	0	0	0	0
IGHV3S39	IGHJ7	0	0	0	0	0	0	0	0	0	0	0	0	0	0	0
IGHV3S57	IGHJ6	0	0.1	0	0	0	0	0	0	0	0	0.4	0.3	0.2	0.1	0.1
IGHV3S66	NA	0	0	0	0	0	0	0	0	0	0	0	0	0.1	0	0
IGHV3S65	IGHJ2	0	0.2	0.1	0	0.2	0	0	0	0	0	0	0	0	0	0
IGHV3S53	IGHJ5	0	0	0.1	0	0.1	0.1	0	0	0	0	0	0	0.1	0	0
IGHV3S51	IGHJ6	0	0	0	0	0	0	0	0	0	0	0	0	0	0	0
IGHV3S30	IGHJ4	0	0	0	0	0	0	0	0	0	0	0	0	0	0	0
IGHV3S62	IGHJ5	0	0	0	0	0	0	0	0	0	0	0	0	0	0	0
IGHV3S54	IGHJ5	0	0	0	0	0	0	0	0	0	0	0	0	0	0	0
IGHV3S5	IGHJ4	0	0	0	0	0	0	0	0	0	0	0	0	0	0	0
IGHV3S61	IGHJ1	0	0	0	0	0	0	0	0	0	0	0	0	0	0	0
IGHV2S1	IGHJ6	0	0	0	0	0	0	0	0	0	0	0	0	0	0	0
IGHV3S6	IGHJ4	0	0	0.1	0	0	0.1	0	0	0	0	0	0	0.1	0	0
IGHV3S33	IGHJ6	0	0	0	0	0.1	0.4	0	0	0	0.1	0	0	0	0	0
IGHV3S59	IGHJ6	0	0.1	0	0	0	0	0	0	0	0	0	0	0.1	0	0.1
IGHV3-1	IGHJ2	0	0	0	0	0	0	0	0	0	0	0	0	0	0	0
IGHV3S36	IGHJ4	0	0	0	0	0	0	0	0	0	0	0	0	0	0	0
IGHV2S1	IGHJ4	0	0	0	0	0	0	0	0	0	0	0	0	0	0	0
IGHV3S60	IGHJ2	0	0	0	0	0	0	0	0	0	0	0	0	0	0	0
IGHV2S2	IGHJ6	0	0	0	0	0	0	0	0	0	0	0	0	0	0	0
IGHV3S68	IGHJ6	0	0	0	0	0	0	0	0	0	0	0	0	0	0	0
IGHV2S5	IGHJ2	0	0	0	0	0	0	0	0	0	0	0	0	0	0	0
IGHV3S63	IGHJ1	0	0	0	0	0	0	0	0	0	0	0	0	0	0	0
IGHV3S20	IGHJ6	0	0	0	0	0	0	0	0	0	0	0	0	0	0	0
IGHV3S62	NA	0	0	0	0	0	0	0	0	0	0	0	0	0	0.1	0
IGHV3S54	IGHJ2	0	0	0	0	0	0	0	0	0	0	0	0	0	0.1	0.1
IGHV3S56	IGHJ6	0	0	0	0	0	0	0	0	0	0	0	0	0	0	0
IGHV3S63	IGHJ3	0	0	0.1	0	0	0	0	0	0	0	0	0	0	0	0
IGHV3S54	IGHJ7	0	0	0	0	0	0	0	0	0	0	0	0	0	0	0
IGHV3S14	IGHJ4	0	0	0	0	0	0	0	0	0	0	0	0	0	0	0
IGHV3S33	IGHJ4	0	0	0	0	0	0	0	0	0	0.1	0	0	0	0	0
IGHV3S41	IGHJ2	0	0.1	0	0	0	0	0	0	0	0	0	0	0	0	0
IGHV3S55	IGHJ6	0	0	0.3	0	0	0.2	0.2	0.1	0.6	0.4	0.2	0.3	0.1	0	0.2
IGHV3S32	NA	0	0	0	0	0	0	0	0	0	0	0	0	0	0	0
IGHV3S30	IGHJ6	0	0	0	0	0	0	0	0	0	0	0	0	0.1	0	0
IGHV3S61	NA	0	0	0	0	0	0	0	0	0	0	0	0	0	0	0
IGHV3S13	IGHJ4	0	0	0	0	0	0	0	0	0	0	0	0	0	0	0
IGHV3S8	IGHJ4	0	0	0	0	0	0	0	0	0	0	0	0	0	0	0
IGHV3S57	IGHJ4	0	0	0	0	0	0	0	0	0	0	0	0	0	0	0
IGHV3S35	IGHJ6	0	0	0	0	0.1	0	0	0	0	0	0	0	0	0	0
IGHV3S5	IGHJ7	0	0	0	0	0	0	0	0	0	0	0	0	0	0	0
IGHV3S61	IGHJ5	0	0	0	0	0	0	0.1	0	0	0	0	0	0	0	0
IGHV3S19	IGHJ6	0	0	0	0	0	0	0	0	0	0	0	0	0	0	0
IGHV3S66	IGHJ5	0	0	0	0	0	0	0	0	0	0	0	0	0	0	0
IGHV3-3	IGHJ7	0	0	0	0	0	0	0	0	0	0	0	0	0	0	0
IGHV3S40	IGHJ7	0	0	0	0	0	0	0	0	0	0	0	0	0	0	0
IGHV3S29	IGHJ6	0	0	0	0	0	0	0	0	0	0	0	0	0	0	0
IGHV3S17	IGHJ6	0	0	0	0	0	0	0	0	0	0	0	0	0	0	0
IGHV3S45	IGHJ4	0	0	0	0	0	0	0	0	0	0	0	0	0	0	0
IGHV2S5	IGHJ7	0	0	0	0	0	0	0	0	0	0	0	0	0	0	0
IGHV3S64	IGHJ5	0	0	0	0	0	0	0	0	0	0	0	0	0	0	0
IGHV3S29	IGHJ4	0	0	0	0	0	0	0	0	0	0	0	0	0	0	0
IGHV3S31	IGHJ1	0	0	0	0	0	0	0	0	0	0	0	0	0	0	0
IGHV3S37	NA	0	0	0	0	0	0	0	0	0	0	0	0	0	0	0
IGHV3S9	IGHJ4	0	0	0	0	0	0	0	0	0	0	0	0	0	0	0
IGHV3S54	IGHJ3	0	0	0	0	0	0	0	0	0	0	0	0	0	0	0
IGHV3-3	IGHJ6	0	0	0	0	0	0	0	0	0	0	0	0	0	0	0
IGHV2S2	IGHJ7	0	0	0	0	0	0	0	0	0	0	0	0	0	0	0
IGHV3S32	IGHJ7	0	0	0	0	0	0	0	0	0	0	0	0	0	0	0
IGHV3S19	IGHJ4	0	0	0	0	0	0	0	0	0	0	0	0	0	0	0
IGHV3-3	IGHJ4	0	0	0	0	0	0	0	0	0	0	0	0	0	0	0
IGHV3S42	IGHJ4	0	0	0	0	0	0	0	0	0	0	0	0	0	0	0
IGHV3S39	IGHJ2	0	0	0	0	0	0	0	0	0	0	0	0	0	0	0
IGHV3S12	IGHJ7	0	0	0	0	0	0	0	0.1	0.2	0	0.1	0	0	0	0
IGHV3S41	IGHJ5	0	0	0	0	0	0	0	0	0	0	0	0	0	0	0
IGHV3S64	IGHJ2	0	0	0	0	0	0	0	0	0	0	0	0	0	0	0
IGHV3S9	IGHJ6	0	0	0	0	0	0	0	0	0	0	0	0	0	0	0
IGHV3-2	IGHJ7	0	0	0	0.1	0	0	0	0	0	0	0	0	0	0	0
IGHV3S20	IGHJ4	0	0	0	0	0	0	0	0	0	0	0	0	0	0	0
IGHV3S63	IGHJ5	0	0	0	0	0	0	0	0	0	0	0	0	0	0	0
IGHV3S6	IGHJ7	0	0	0	0	0	0	0	0	0	0	0	0	0	0	0
IGHV3S8	IGHJ6	0	0	0	0	0	0	0	0	0	0	0	0	0	0	0
IGHV3S17	IGHJ7	0	0	0	0	0	0	0	0	0	0	0	0	0	0	0
IGHV3S35	IGHJ4	0	0	0	0	0	0	0	0	0	0	0	0	0	0	0
IGHV3S51	IGHJ4	0	0	0	0	0	0	0	0	0	0	0	0	0	0	0
IGHV3S1	NA	0	0	0	0	0	0	0	0	0	0	0	0	0	0	0

IGHV3S57	IGHJ3	0	0	0	0	0	0	0	0	0	0	0	0	0	0	0
IGHV3S62	IGHJ1	0	0	0	0	0	0	0	0	0	0	0	0	0	0	0
IGHV2S9	IGHJ6	0	0	0	0	0	0	0	0	0	0	0	0	0	0	0
IGHV3S13	IGHJ6	0	0	0	0	0	0	0	0	0	0	0	0	0	0	0
IGHV3S2	IGHJ4	0	0	0	0	0	0	0	0	0	0	0	0	0	0	0
IGHV3S33	IGHJ3	0	0	0	0	0	0	0	0	0	0	0	0	0	0	0
IGHV3S37	IGHJ7	0	0	0	0	0	0	0	0	0	0	0	0	0	0	0
IGHV3S40	IGHJ1	0	0	0	0	0	0	0	0	0	0	0	0	0	0	0
IGHV3S44	IGHJ7	0	0	0	0	0	0	0	0	0	0	0	0	0	0	0
IGHV3S56	IGHJ7	0	0	0	0	0	0	0	0	0	0	0	0	0	0	0
IGHV3S60	IGHJ7	0	0	0	0	0	0	0	0	0	0	0	0	0	0	0
IGHV3S63	NA	0	0	0	0	0	0	0	0	0	0	0	0	0	0	0
IGHV3S67	IGHJ4	0	0	0	0	0	0	0	0	0.2	0.1	0	0.1	0.1	0.2	0.1
IGHV3S6	IGHJ1	0	0	0	0	0	0	0	0	0	0	0	0	0	0	0
IGHV3S13	IGHJ7	0	0	0	0	0	0	0	0	0	0	0	0	0	0	0
IGHV3S42	IGHJ6	0	0	0	0	0	0	0	0	0	0	0	0	0	0	0
IGHV2S1	IGHJ2	0	0	0	0	0	0	0	0	0	0	0	0	0	0	0
IGHV3S12	NA	0	0	0	0	0	0	0	0	0	0	0	0	0	0	0
IGHV3S15	IGHJ4	0	0	0	0	0	0	0	0	0	0	0	0	0	0	0
IGHV3S16	IGHJ7	0	0	0	0	0	0	0	0	0	0	0	0	0	0	0
IGHV3S18	IGHJ6	0	0	0	0	0	0	0	0	0	0	0	0	0	0	0
IGHV3S1	IGHJ5	0	0	0	0	0	0	0	0	0	0	0	0	0	0	0
IGHV3S20	IGHJ7	0	0	0	0	0	0	0	0	0	0	0	0	0	0	0
IGHV3S26	IGHJ6	0	0	0	0	0	0	0	0	0	0	0	0	0	0	0
IGHV3S28	IGHJ3	0	0	0	0	0	0	0	0	0	0	0	0	0	0	0
IGHV3S29	IGHJ3	0	0	0	0	0	0	0	0	0	0	0	0	0	0	0
IGHV3S2	IGHJ6	0	0	0	0	0	0	0	0	0	0	0	0	0	0	0
IGHV3S37	IGHJ1	0	0	0	0	0	0	0	0	0	0	0	0	0	0	0
IGHV3S51	IGHJ2	0	0	0	0	0	0	0	0	0	0	0	0	0	0	0
IGHV3S65	IGHJ5	0	0	0	0	0	0	0	0	0	0	0	0	0	0	0
IGHV3S67	IGHJ6	0	0	0	0	0	0	0	0	0	0	0	0	0	0	0
IGHV3S68	IGHJ4	0	0	0	0	0	0	0	0	0	0	0	0	0	0	0
IGHV3S68	IGHJ7	0	0	0	0	0	0	0	0	0	0	0	0	0	0	0
IGHV3S34	IGHJ2	0	0	0	0	0	0	0	0	0	0	0	0	0	0	0
IGHV3S42	NA	0	0	0	0	0	0	0	0	0	0	0	0	0	0	0
IGHV3S5	IGHJ3	0	0	0	0	0	0	0	0	0	0	0	0	0	0	0
IGHV2S1	IGHJ3	0	0	0	0	0	0	0	0	0	0	0	0	0	0	0
IGHV2S1	IGHJ7	0	0	0	0	0	0	0	0	0	0	0	0	0	0	0
IGHV2S2	IGHJ2	0	0	0	0	0	0	0	0	0	0	0	0	0	0	0
IGHV2S2	IGHJ3	0	0	0	0	0	0	0	0	0	0	0	0	0	0	0
IGHV2S2	IGHJ5	0	0	0	0	0	0	0	0	0	0	0	0	0	0	0
IGHV2S5	IGHJ3	0	0	0	0	0	0	0	0	0	0	0	0	0	0	0
IGHV2S5	NA	0	0	0	0	0	0	0	0	0	0	0	0	0	0	0
IGHV2S8	IGHJ7	0	0	0	0	0	0	0	0	0	0	0	0	0	0	0
IGHV2S9	IGHJ2	0	0	0	0	0	0	0	0	0	0	0	0	0	0	0
IGHV2S9	IGHJ4	0	0	0	0	0	0	0	0	0	0	0	0	0	0	0
IGHV3-1	IGHJ5	0	0	0	0	0	0	0	0	0	0	0	0	0	0	0
IGHV3-2	IGHJ1	0	0	0	0	0	0	0	0	0	0	0	0	0	0	0
IGHV3-2	IGHJ2	0	0	0	0	0	0	0	0	0	0	0	0	0	0	0
IGHV3-2	IGHJ5	0	0	0	0	0	0	0	0	0	0	0	0	0	0	0
IGHV3-2	NA	0	0	0	0	0	0	0	0	0	0	0	0	0	0	0
IGHV3-3	IGHJ2	0	0	0	0	0	0	0	0	0	0	0	0	0	0	0
IGHV3-3	IGHJ3	0	0	0	0	0	0	0	0	0	0	0	0	0	0	0
IGHV3S10	IGHJ3	0	0	0	0	0	0	0	0	0	0	0	0	0	0	0
IGHV3S10	IGHJ7	0	0	0	0	0	0	0	0	0	0	0	0	0	0	0
IGHV3S12	IGHJ2	0	0	0	0	0	0	0	0	0	0	0	0	0	0	0
IGHV3S12	IGHJ3	0	0	0	0	0	0	0	0	0	0	0	0	0	0	0
IGHV3S13	IGHJ2	0	0.1	0	0	0	0	0	0	0	0	0	0	0	0	0
IGHV3S13	IGHJ5	0	0	0	0	0	0	0	0	0	0	0	0	0	0	0
IGHV3S14	IGHJ7	0	0	0	0	0	0	0	0	0	0	0	0	0	0	0
IGHV3S15	IGHJ2	0	0	0	0	0	0	0	0	0	0	0	0	0	0	0
IGHV3S15	IGHJ6	0	0	0	0	0	0	0	0	0	0	0	0	0	0	0
IGHV3S15	IGHJ7	0	0	0	0	0	0	0	0	0	0	0	0	0	0	0
IGHV3S15	NA	0	0	0	0	0	0	0	0	0	0	0	0	0	0	0
IGHV3S16	IGHJ6	0	0	0	0	0	0	0	0	0	0	0	0	0	0	0
IGHV3S17	IGHJ1	0	0	0	0	0	0	0	0	0	0	0	0	0	0	0
IGHV3S17	IGHJ4	0	0	0	0	0	0	0	0	0	0	0	0	0	0	0
IGHV3S19	IGHJ7	0	0	0	0	0	0	0	0	0	0	0	0	0	0	0
IGHV3S1	IGHJ1	0	0	0	0	0	0	0	0	0	0	0	0	0	0	0
IGHV3S24	IGHJ4	0	0	0	0	0	0	0	0	0	0	0	0	0	0	0
IGHV3S25	IGHJ2	0	0	0	0	0	0	0	0	0	0	0	0	0	0	0
IGHV3S25	IGHJ3	0	0	0	0	0	0	0	0	0	0	0	0	0	0	0
IGHV3S25	IGHJ5	0	0	0	0	0	0	0	0	0	0	0	0	0	0	0
IGHV3S25	IGHJ7	0	0	0	0	0	0	0	0	0	0	0	0	0	0	0
IGHV3S25	NA	0	0	0	0	0	0	0	0	0	0	0	0	0	0	0
IGHV3S26	IGHJ4	0	0	0	0	0	0	0	0	0.1	0	0	0	0	0	0
IGHV3S26	IGHJ7	0	0	0	0	0	0	0	0	0	0	0	0	0	0	0
IGHV3S26	NA	0	0	0	0	0	0	0	0	0	0	0	0	0	0	0
IGHV3S27	IGHJ2	0	0	0	0	0	0	0	0	0	0	0	0	0	0	0
IGHV3S27	IGHJ6	0	0	0	0	0	0	0	0	0	0	0	0	0	0	0
IGHV3S28	IGHJ1	0	0	0	0	0	0	0	0	0	0	0	0	0	0	0
IGHV3S28	IGHJ2	0	0	0	0	0	0	0	0	0	0	0	0	0	0	0
IGHV3S29	IGHJ2	0	0	0	0	0	0	0	0	0	0	0	0	0	0	0

IGHV3S29	IGHJ7	0	0.1	0	0	0	0	0	0	0	0	0	0	0	0	0
IGHV3S29	NA	0	0	0	0	0	0	0	0	0	0	0	0	0	0	0
IGHV3S2	IGHJ7	0	0	0	0	0	0	0	0	0	0	0	0	0	0	0
IGHV3S30	IGHJ7	0	0	0	0	0	0	0	0	0	0	0	0	0	0	0
IGHV3S31	IGHJ3	0	0	0	0	0	0	0	0	0	0	0	0	0	0	0
IGHV3S31	IGHJ7	0	0	0	0	0	0	0	0	0	0	0	0	0	0	0
IGHV3S32	IGHJ2	0	0	0	0	0	0	0	0	0	0	0	0	0	0	0
IGHV3S32	IGHJ3	0	0	0	0	0.1	0	0	0	0	0	0	0	0	0	0
IGHV3S33	IGHJ5	0	0	0	0	0	0	0	0	0	0	0	0	0	0	0
IGHV3S33	IGHJ7	0	0	0	0	0	0	0	0	0	0	0	0	0	0	0
IGHV3S34	IGHJ1	0	0	0	0	0	0	0	0	0	0	0	0	0	0	0
IGHV3S34	IGHJ3	0	0	0	0	0	0	0	0	0	0	0	0	0	0	0
IGHV3S34	IGHJ5	0	0	0	0	0	0	0	0	0	0	0	0	0	0	0
IGHV3S34	IGHJ7	0	0	0	0	0	0	0	0	0	0	0	0	0	0	0
IGHV3S34	NA	0	0	0	0	0	0	0	0	0	0	0	0	0	0	0
IGHV3S35	IGHJ3	0	0	0	0	0	0	0	0	0	0	0	0	0	0	0
IGHV3S35	IGHJ7	0	0	0	0	0	0	0	0	0	0	0	0	0	0	0
IGHV3S36	IGHJ1	0	0	0	0	0	0	0	0	0	0	0	0	0	0	0
IGHV3S36	IGHJ2	0	0	0	0	0	0	0	0	0	0	0	0	0	0	0
IGHV3S36	IGHJ3	0	0	0	0	0	0	0	0	0	0	0	0	0	0	0
IGHV3S36	IGHJ5	0	0	0	0	0	0	0	0	0	0	0	0	0	0	0
IGHV3S36	IGHJ7	0	0	0	0	0	0	0	0	0	0	0	0	0	0	0
IGHV3S36	NA	0	0	0	0	0	0	0	0	0	0	0	0	0	0	0
IGHV3S37	IGHJ2	0	0	0	0	0	0	0	0	0	0	0	0	0	0	0
IGHV3S37	IGHJ3	0	0	0	0	0	0	0	0	0	0	0	0	0	0	0
IGHV3S38	IGHJ4	0	0	0	0	0	0	0	0	0	0	0	0	0	0	0
IGHV3S38	IGHJ6	0	0	0	0	0	0	0	0	0	0	0	0	0	0	0
IGHV3S39	IGHJ1	0	0	0	0	0	0	0	0	0	0	0	0	0	0	0
IGHV3S39	IGHJ3	0	0	0	0	0	0	0	0	0	0	0	0	0	0	0
IGHV3S39	IGHJ5	0	0	0	0	0	0	0	0	0	0	0	0	0	0	0
IGHV3S39	NA	0	0	0	0	0	0	0	0	0	0	0	0	0	0	0
IGHV3S40	IGHJ3	0	0	0	0	0	0	0	0	0	0	0	0	0	0	0
IGHV3S40	IGHJ5	0	0	0	0	0	0	0	0	0	0	0	0	0	0	0
IGHV3S40	NA	0	0	0	0	0	0	0	0	0	0	0	0	0	0	0
IGHV3S41	IGHJ1	0	0	0	0	0	0	0	0	0	0	0	0	0	0	0
IGHV3S42	IGHJ7	0	0	0	0	0	0	0	0	0	0	0	0	0	0	0
IGHV3S43	IGHJ4	0	0	0	0	0	0	0	0	0	0	0	0	0	0	0
IGHV3S43	IGHJ6	0	0	0	0	0	0	0	0	0	0	0	0	0	0	0
IGHV3S44	IGHJ1	0	0	0	0	0	0	0	0	0	0	0	0	0	0	0
IGHV3S44	IGHJ3	0	0	0	0	0	0	0	0	0	0	0	0	0	0	0
IGHV3S44	NA	0	0	0	0	0	0	0	0	0	0	0	0	0	0	0
IGHV3S45	IGHJ6	0	0	0	0	0	0	0	0	0	0	0	0	0	0	0
IGHV3S47	IGHJ2	0	0	0	0	0	0	0	0	0	0	0	0	0	0	0
IGHV3S47	IGHJ3	0	0	0	0	0	0	0	0	0	0	0	0	0	0	0
IGHV3S50	IGHJ6	0	0	0	0	0	0	0	0	0	0	0	0	0	0	0
IGHV3S51	IGHJ1	0	0	0	0	0	0	0	0	0	0	0	0	0	0	0
IGHV3S51	IGHJ3	0	0	0	0	0	0	0	0	0	0	0	0	0	0	0
IGHV3S51	IGHJ5	0	0	0	0	0	0	0	0	0	0	0	0	0	0	0
IGHV3S51	IGHJ7	0	0	0	0	0	0	0	0	0	0	0	0	0	0	0
IGHV3S52	IGHJ6	0	0	0	0	0	0	0	0	0	0	0	0	0	0	0
IGHV3S53	IGHJ1	0	0	0	0	0	0	0	0	0	0	0	0	0	0	0.1
IGHV3S55	IGHJ1	0	0	0	0	0	0	0	0	0	0	0	0	0	0	0
IGHV3S55	IGHJ5	0	0	0	0	0	0	0	0	0	0	0	0	0	0	0
IGHV3S55	IGHJ7	0	0	0	0	0	0	0	0	0	0	0	0	0	0	0
IGHV3S56	IGHJ2	0	0	0	0	0	0	0	0	0	0	0	0	0	0	0
IGHV3S56	IGHJ3	0	0	0	0	0	0	0	0	0	0	0	0	0	0	0
IGHV3S56	IGHJ5	0	0	0	0	0	0	0	0	0	0	0	0	0	0	0
IGHV3S56	NA	0	0	0	0	0	0	0	0	0	0	0	0	0	0	0
IGHV3S57	IGHJ2	0	0	0	0	0	0	0	0	0	0	0	0	0	0	0
IGHV3S57	IGHJ7	0	0	0	0	0	0	0	0	0	0	0	0	0	0	0
IGHV3S58	IGHJ4	0	0	0	0	0	0	0	0	0	0	0	0	0	0	0
IGHV3S58	IGHJ6	0	0	0	0	0	0	0	0	0	0	0	0	0	0	0
IGHV3S59	IGHJ4	0	0	0	0	0	0	0	0	0	0	0	0	0	0	0
IGHV3S5	IGHJ2	0	0	0	0	0	0	0	0	0	0	0	0	0	0	0
IGHV3S60	IGHJ3	0	0	0	0	0	0	0	0	0	0	0	0	0	0	0
IGHV3S60	NA	0	0	0	0	0	0	0	0	0	0	0	0	0	0	0
IGHV3S64	NA	0	0	0	0	0	0	0	0	0	0	0	0	0	0	0
IGHV3S65	IGHJ1	0	0	0	0	0	0	0	0	0	0	0	0	0	0	0
IGHV3S65	IGHJ3	0	0	0	0	0	0	0	0	0	0	0	0	0	0	0
IGHV3S65	NA	0	0	0	0	0	0	0	0	0	0	0	0	0	0	0
IGHV3S67	IGHJ7	0	0	0	0	0	0	0	0	0	0	0	0	0	0	0
IGHV3S6	NA	0	0	0	0	0	0	0	0	0	0	0	0	0	0	0
IGHV3S7	IGHJ3	0	0	0	0	0	0	0	0	0	0	0	0	0	0	0
IGHV3S7	IGHJ6	0	0	0	0	0	0	0	0	0	0	0	0	0	0	0
IGHV3S8	IGHJ1	0	0	0	0	0	0	0	0	0	0	0	0	0	0	0
IGHV3S8	IGHJ5	0	0	0	0	0	0	0	0	0	0	0	0	0	0	0
IGHV3S8	IGHJ7	0	0	0	0	0	0	0	0	0	0	0	0	0	0	0
IGHV3S8	NA	0	0	0	0	0	0	0	0	0	0	0	0	0	0	0
IGHV3S9	IGHJ7	0	0	0	0	0	0	0	0	0	0	0	0	0	0	0

SUPPLEMENTARY TABLE S2 | Examined clones in Top16 clusters in IgG fragment experiment

Cluster[1]	V gene	J gene	Length of CDR-H3[2]	Max % of appearance	Max bit score V[3]	Min bit score V[4]	Examined clone	cDNA sequence	Amino acid sequence with CDRs undelined[2]
Ig-1	IGHV3S53	IGHJ7	17	28.864	313.000	235.000	Ig-L1	CAGGTGCAGCTCGTGGAGTCTGGGGGCGGCTTGGTGCAGCCTGGAGGGTCTCTGAGGCTCTCCTGTACAGCCTCTGGATT<br>CAATCTGGATCGTTGGCCCGTAGGATGGTTCCGCCAGGCCCCAGGGAAGCAGCACGAGGGAGTTGCATGTGTTTCTGAGA<br>GAAATGATAACACATACTATATAGACTCCGTGAAGGGCCGATTCACCATCTCCCGCGACAGTGCTAAGAATACAGTGTAT<br>CTACAGATGAACAGCCTGAAACCCGACGACACGGCCGTTTATCGCTGTTTACCAAGTTGTTCGGTCCTAGGGGATACTAT<br>GCAGGTCCCCTATGGTTCCTGGGGCCGCGGACCCAGGTCACCGTCTCCTCG	QVQLVESGGGLVQPGGSLRLSCTASGFNLDRWPVGWFRQAPGKQHEGVACVSERNDNTYYIDSVKG<br>RFTISRDSAKNTVYLQMNSLKPDDTAVYRCLPSCSVLGDTMQVPYGSWGRGTQVTVSS
Ig-2	IGHV3S66	IGHJ4	24	26.111	359	209	Ig-S11	CAGGTGCAGCTCGTGGAGTCTGGGGGAGGCTTGGTGCAGGCTGGGGGGTCTCTAAAACTCTCCTGTGCAGTCTCTGGATT<br>CGATTCCGACGATTATGCCGTAGGCTGGTTCCGCCAGGCCCCAGGGAAAGAGCGTGAGGGGGTCGCATGTATTAGGTTTA<br>CTGATGGCAACACCTTCTATGCAAACTCAGCGAAGGGCCGATTCACCATTTCCACTGACAACGCCGGCAACTCGGTACAT<br>CTGCAAATGAACAGCCTGAAACCTGAAGACACGGGCAGTTATGTCTGTGCAGCGTCTATCGGCTCCCACGTTTTTGGAGA<br>CCGGTTCCGGCCTCTATCGGCAATCAGCTATGAGTTCTGGGGCCAGGGGACTCAGGTCGCCGTCTCCTCA	QVQLVESGGGLVQAGGSLKLSCAVSGFDSDDYAVGWFRQAPGKEREGVACIRFTDGNTFYANSAKG<br>RFTISTDNAGNSVHLQMNSLKPEDTGSYVCAASIGSHVFGDRFRPLSAISYEFWGQGTQVAVSS
Ig-3	IGHV3S66	IGHJ6	17	4.644	370	337	Ig-S43	CAGGTGCAGCTCGTGGAGTCTGGGGGAGGCTTGGTGCAGCCTGGGGGGTCTCTGAGACTTTCCTGTGACGTCTCGGGATT<br>CTCTTTCGATGATTATGCCATAGCCTGGTTCCGCCAGGTCCCAGAGAAGGAGCGTGAGGGCATAGCTGTCATTAGTAGTA<br>GTGATAGTACTACAGAATACGCAGACTCCGTGAAGGGCCGATTCACCATCTCCTTTGACGACACCAAGACGACGGTGTAT<br>CTGCAGCTGAACAGCCTGAAACCCGAAGACACGGCCGTTTATTACTGTGCCGCAGACTACGCGGACTTACTACGTCTAGA<br>CCCAACTGACTATGGATTGTGGGGCCAGGGGACCCAGGTCACCGTCTCCCCG	QVQLVESGGGLVQPGGSLRLSCDVSGFSFDDYAIAWFRQVPEKEREGIAVISSSDSTTEYADSVKG<br>RFTISFDDTKTTVYLQLNSLKPEDTAVYYCAADYADLLRLDPTDYGLWGQGTQVTVSP
Ig-4	IGHV3S61	IGHJ4	17	3.599	300.000	244.000	Ig-S38	CAGGTGCAGCTCGTGGAGTCTGGGGGAGGACTGGTGCAGTCTGGGGGGTCTCTGAGACTCTCTTGTGTAGCCTCTGGATT<br>CAACACGGATTATTATTCGATAGCGTGGTTTCGCCAGGCCCCAGGGAAACAACGCGAGGGGGTCACTTGTATTCCGAGTA<br>CCCTTAGTAATTTTAATGGTAGTATTCACTATTCAGACTCCGTGAAGGGCCGCTTTACCATCTCCAGAGACGATGCCCGG<br>AAGACGGTCTATCTCCAAATGAACAACCTGAAACCTGAAGACACGGCCATCTATTACTGTGCGACAGAGGCCTGGGGTCC<br>CTTTTGTTACTGCTCAGGTTATGGTTGTGGCAACTGGGGCCAGGGGACCCAGGTCACCGTCTCCTCA	QVQLVESGGGLVQSGGSLRLSCVASGFNTDYYSIAWFRQAPGKQREGVTCIPSTLSNFNGSIHYSD<br>SVKGRFTISRDDARKTVYLQMNNLKPEDTAIYYCATEAWGPFCYCSGYGCGNWGQGTQVTVSS
Ig-5	IGHV3S53	IGHJ4	14	2.718	399.000	350.000	Ig-L38	CAGGTGCAGCTCGTGGAGTCTGGGGGAGGCTTGGTACAGGCTGGGGGGTCTCTGAGACTCTCCTGTGCAGCCTCTGGAAG<br>CAATTTCGAGATCAATGTCATGCGCTGGTACCGCCAGGCTCCAGGGAAGCAGCGCGAGTTGGTCGCAATGCTTGCTCATA<br>ATGGAGGCACAAACTATGTAGACTCCGTGAAGGGCCGATTCACCATCTCCAGAGACAGCGCCAAGAACACGGTGTATCTA<br>CAGATGAATAGCCTGAAACCTGAGGACACAGCCGTCTATTACTGTAATTTTTTCCTACAGAGTCCCCTACAAGGGAAGTA<br>TCTTTACTGGGGCCAGGGTACCCAGGTCACCGTCTCCTCA	QVQLVESGGGLVQAGGSLRLSCAASGSNFEINVMRWYRQAPGKQRELVAMLAHNGGTNYVDSVKGR<br>FTISRDSAKNTVYLQMNSLKPEDTAVYYCNFFLQSPLQGKYLYWGQGTQVTVSS
Ig-6	IGHV3S53	IGHJ4	14	1.818	372.000	322.000	Ig-L8	CAGGTGCAGCTCGTGGAGTCTGGGGGAGGCTTGGTGCAGGCTGGGGGGTCTCTGGGACTCTCCTGTGTAGCCTCTGGAAA<br>CATCTTCAGTCTCAATACCATGGGCTGGTACCGCCAGGCTCCCGGAACCCAGCGCGAGTTGGTCGCAACTATTACAAGAG<br>GTCATGCTACGATCTACGCAGACTCCGTGAAGGGCCGATTCACCATCTCCAGAGACAACGAGAAGGTCTCGGTGTATCTG<br>CATATGAACAAACTGAAACCTGAGGACACAGCCATCTATTACTGTACTGCAAGTCTTTACGGTAGTAGCCGTCCCCAGTA<br>TGACTACTGGGGCCAGGGGACCCAGGTCACCGTCTCCTCA	QVQLVESGGGLVQAGGSLGLSCVASGNIFSLNTMGWYRQAPGTQRELVATITRGHATIYADSVKGR<br>FTISRDNEKVSVYLHMNKLKPEDTAIYYCTASLYGSSRPQYDYWGQGTQVTVSS
Ig-7	IGHV3S66	IGHJ6	20	1.816	508	409	Ig-S1139	CAGGTGCAGCTCGTGGAGTCTGGGGGAGGCTTGGTGCAGGCTGGGGGGTCTCTGAGACTCTCCTGTGCAGCCTCTGGATT<br>CACTTTCGATGATTATGTCATAGGCTGGTTCCGCCAGGCCCCAGGGAAGGAGCGCGAGGGGGTCTCATGTATTAGTAGTA<br>GTGATGGTAGCACATACTATGCAGACTCCGTGAAGGGCCGATTCACCATCTCCAGTGACAACGCCAAGAACACGGTGTAT<br>CTGCAAATGAACAGCCTGAAACCTGAGGACACGGCCGTTTATTACTGTGCAGAAGGACCCACGGTCCTAGACGGATGTAT<br>AGTGGATAGTGGTAGTTACTATTTTTCCTGGGGCCAGGGGACCCAGGTCACCGTCTCCTCG	QVQLVESGGGLVQAGGSLRLSCAASGFTFDDYVIGWFRQAPGKEREGVSCISSSDGSTYYADSVKG<br>RFTISSDNAKNTVYLQMNSLKPEDTAVYYCAEGPTVLDGCIVDSGSYYFSWGQGTQVTVSS
Ig-8	IGHV3S31	IGHJ6	20	1.748	337.000	309.000	Ig-S176	CAGGTGCAGCTCGTGGAGTCTGGGGGAGGGTCGGTGCAGCCTGGGGGGTCTCTGAGACTCTCCTGCGCAGCCTCTGGATT<br>CGCCTTCAGAAATTATGCCATGGCTTGGGTCCGCCAGACTCCAGGAAAGGAACTCGAGTGGGTCTCGACAATTAACCGTG<br>CCGGTGATAGCGCATACTATGCAGACTCCGTGAACGAGCGATTCACCATTTCCAGAGACAACGACAAGAATACATTGTAT<br>CTGCAAATGAACAGCCTGAAACCTGAGGACGCGGCCGTGTATATCTGTACAAAATATGTGTTTGCAAGTTCAGAACTCCC<br>CATTTTTCGAGCTGCCGACTTCCCAAAAAGGGGCCAGGGGACCCAGGTCACCGTCTCCTCG	QVQLVESGGGSVQPGGSLRLSCAASGFAFRNYAMAWVRQTPGKELEWVSTINRAGDSAYYADSVNE<br>RFTISRDNDKNTLYLQMNSLKPEDAAVYICTKYVFASSELPIFRAADFPKRGQGTQVTVSS
Ig-9	IGHV3S61	IGHJ6	16	1.624	411.000	372.000	Ig-L16	CAGGTGCAGCTCGTGGAGTCTGGGGGAGGCTCGGTGCAGCCTGGGGGGTCTCTGAGACTCTCCTGTGCAGCCGTTGGATT<br>CAGTTTGGATGTTTATTCCATAGGCTGGTTCCGCCAGGCCCCAGGGAAGGAGCGCGAGGGGGTCTCATGTATTAGTAGTA<br>ATGATGGTACCACTTACTATTCAGACTCCGTGAAGGGCCGATTCACCATCTCCAAGGACAGGTTCAGGAACACGGCGTAT<br>CTGCAAATGAACAGCCTGAAACCTGATGACACGGCCGTTTATTACTGTGCGACAGGGGCCTGCTATTTAAGTAGTGGTTT<br>CTACAATTTTGGTTCCTGGGGCCGGGGGACCCAGGTCACCGTCGCCTCG	QVQLVESGGGSVQPGGSLRLSCAAVGFSLDVYSIGWFRQAPGKEREGVSCISSNDGTTYYSDSVKG<br>RFTISKDRFRNTAYLQMNSLKPDDTAVYYCATGACYLSSGFYNFGSWGRGTQVTVAS
Ig-10	IGHV3S53	IGHJ4	15	1.154	411.000	344.000	Ig-L19	CAGGTGCAGCTCGTGGAGTCTGGGGGAGGCTTGGTGCAGACTGGGGGGTCTCTGAGACTCTCCTGTGCAGCCTCTGGAAC<br>CATCCTCGGTGTCGATGCCATGGCCTGGTACCGCCAGGCTCCAGGGAAGGTGCGCGAGTCGGTCGCAACTATTACTAGTC<br>ATGGTATCACAAGATATATAGACTCCGTGAAGGGCCGATTCACCATCTCCAGAGACAACGCCAAGAACACCTTATATCTG<br>CAAATGAACAGCCTGAAACCTGAGGACGCGGCCGTCTACTACTGTTATGCAATGATTAGGCCACGCAATAGTCCGTCGTA<br>TTCTCCCTACTGGGGCCAGGGGACCCAGGTCACCGTCTCCTCA	QVQLVESGGGLVQTGGSLRLSCAASGTILGVDAMAWYRQAPGKVRESVATITSHGITRYIDSVKGR<br>FTISRDNAKNTLYLQMNSLKPEDAAVYYCYAMIRPRNSPSYSPYWGQGTQVTVSS
Ig-11	IGHV3S66	IGHJ2	20	1.134	342	292	Ig-L29	CAGGTGCAGCTCGTGGAGTCTGGGGGAGGCTTGGTGCAGGCTGGACAGTCTCTGAGACTCTCCTGTGCCGTCTCCGGATT<br>CGATTCTGATTACAGTTTGGAGGATTATGCCATAAGTTGGTTTCGCCAGGCCCCAGGGAAGGAGCGTGAGGGGGTCTCAT<br>GTATTAGTGTGAGTGATGATATGATCTATTATGCCGACTCCGTGAAGGGGCGATTCTCCATCTCCAGTGACAACGCCAAG<br>AGCACGGTTTATCTGCAAATGAATCACTTGTCACCTAGTGACACGGCCGTTTATTACTGTGCAGCAGAGTTCGGAAGACC<br>TGGTAGTACGTGGGCCCTATCTGAGTCGTGGTATCCTGATTGGGGCCCGGGCACCCAGGTCACTGTCTCCTCA	QVQLVESGGGLVQAGQSLRLSCAVSGFDSDYSLEDYAISWFRQAPGKEREGVSCISVSDDMIYYAD<br>SVKGRFSISSDNAKSTVYLQMNHLSPSDTAVYYCAAEFGRPGSTWALSESWYPDWGPGTQVTVSS

Ig-12	IGHV3S1	IGHJ4	17	1.065	276.000	215.000	Ig-S155	CAGGTGCAGCTCGTGGAGTCTGGAGGAGACTCGGTGGAAGCCGGGGGGTCTCTGAGACTCTCCTGCGCAGCCTCTGGATT<br>CATCTTCAATAAATATTGGATGTATTGGGTCCGTCGGGCTCCCGGAAAGGAGTTTGAATGGGTCTCGGCGATTAGTACAA<br>ATGGCGAAAACGTCCTCTATAATGACTTCGTGAAGGGTCGCTTTAGCATCTCCAGAGACAACGCCAAGGACACACTTTAT<br>CTACAGATGGACAGACTACAATCTAATGACACGGGCATCTATTACTGTGCGAATGGTTCGCCCCCCGATCCAAGCATGTC<br>CGACTATGCGTTTGACTCTTGGGGTCAGGGGACCCAGGTCACCGTCTCCTCG	QVQLVESGGDSVEAGGSLRLSCAAS<u>GFIFNKYW</u>MYWVRRAPGKEFEWVSA<u>ISTNGENV</u>LYNDFVKG<br>RFSISRDNAKDTLYLQMDRLQSNDTGIYYC<u>ANGSPPDPSMSDYAFDS</u>WGQGTQVTVSS
Ig-13	IGHV3S53	IGHJ6	12	1.043	322.000	283.000	Ig-L39	CAGGTGCAGCTCGTGGAGTCTGGGGGAGGCTTGGTGCAGGCTGGCGGGTCTCTGACCCTCTCCTGTGCAGTAGTCTCTGG<br>AAACTTCTTCGGCATCAATACCATGGGCTGGTACCGCCAGGCTCCAGGGAAGCAGCGCGAGTCGGTCGCAACTATTACAC<br>GTGGTGGTACTAAGAATTATGGGGATGCCGTGAAGGGCCGATTCATCATCTCCAGAGACAACACCAGGAAGACGGTGTCT<br>CTGCAAATGAATAACCTGAGCCCTGAGGACACAGGCGTCTATTACTGTAAAGCTGAACCTTGGGGCCCGCCAATGCCGGA<br>TTCTTGGGGCCCGGGGACCCAGGTCACCGTCTCCTCG	QVQLVESGGGLVQAGGSLTLSCAVV<u>SGNFFGINT</u>MGWYRQAPGKQRESVAT<u>ITRGGTK</u>NYGDAVKG<br>RFIISRDNTRKTVSLQMNNLSPEDTGVYYC<u>KAEPWGPPMPDS</u>WGPGTQVTVSS
Ig-14	IGHV3S63	IGHJ7	15	0.981	333.000	311.000	Ig-S126	CAGGTGCAGCTCGTGGAGTCTGGTGGAGGCTTGGTCCAGACTGGGGGTTCTCTGGGACTCTCCTGTGTAGTCTCTGGAAG<br>CGGTTCGGAATACTATTCCATAGCCTGGTTCCGCCAGGCCCCAGGGAAGGAGCGCGAGGGGGTCGCATGTATTGATAGTA<br>GTTCTGGACGCACAATATATGGAGACTCCGTGAGGGGCCGATTCACCATCTCCAGAGACAACGCCAAGAACACGGTATAC<br>CTGCAGATGGACAACCTGACACCTGAGGACACGGCCGTTTATTCCTGTGCAGCCACAATTATCCCGACTATATTAAAATC<br>CGGCATGGACTACTGGGGCAAAGGGACCCGGGTCACCGTCTCCTCA	QVQLVESGGGLVQTGGSLGLSCVVS<u>GSGSEYYS</u>IAWFRQAPGKEREGVAC<u>IDSSSGRT</u>IYGDSVRG<br>RFTISRDNAKNTVYLQMDNLTPEDTAVYSC<u>AATIIPTILKSGMDY</u>WGKGTRVTVSS
<u>Ig-15</u>	IGHV3S53	IGHJ4	12	0.946	433.000	316.000	Ig-L926	CAGGTGCAGCTCGTGGAGTCTGGGGGAGGTTTGGTGCAGGCTGGGGGATCTCTGAGACTCTCCTGTGCAGCCTCTGGAAT<br>CAGCTTGCGTGATGATAACATGGGCTGGTACCGCCAGGCTCCAGGGAAGCAGCGCGATTTGGTCGCGCTTATTGATAAGT<br>ACAATACCACAAACTATGTAGACTCCGTGAAGGGCCGATTCAGCCTCTCCATAGACAACGCCAAGAACACGGCCTATCTG<br>CAAATGAACAGCCTGAAACCTGAGGACACGGCCGTCTATTACTGTAATGCACTTGGTACCTGGATCCGGGCCGGCCCATA<br>TTGGGGCCAGGGGACCCAGGTCACCGTCTCCTCA	QVQLVESGGGLVQAGGSLRLSCAAS<u>GISLRDDN</u>MGWYRQAPGKQRDLVAL<u>IDKYNTT</u>NYVDSVKGR<br>FSLSIDNAKNTAYLQMNSLKPEDTAVYYC<u>NALGTWIRAGPY</u>WGQGTQVTVSS
<u>Ig-16</u>	IGHV3S61	IGHJ4	16	0.926	521.000	388.000	Ig-L792	CAGGTGCAGCTCGTGGAGTCTGGGGGAGGCTTGGTGCAGCCTGGGGGGTCTCTGAGACTCTCCTGTGCAGCCTCTGGATT<br>AACTTTGAGTTATTGGGCCATAGGCTGGTTCCGCCAGGGCCCAGGGAAGGAGCGCGAGCGGGTCGCATGTATTAGTACTT<br>ATGATGGTACCACGACCTATGGAGACTCCGTGAAGGGCCGATTCACCATCTCCAGAGACAATTACAAGAACACGGTGTAT<br>CTGCAAATGAACAGCCTGAAACCTGAGGACACGGCCCTTTATTACTGTGCGACAGTTGGCTCGGGGTATTACTACTGCTC<br>AGGCAATCCTGACTTGTGGGGCCAGGGGACCCAGGTCACCGTCTCCTCA	QVQLVESGGGLVQPGGSLRLSCAAS<u>GLTLSYWA</u>IGWFRQGPGKERERVAC<u>ISTYDGTT</u>TYGDSVKG<br>RFTISRDNYKNTVYLQMNSLKPEDTALYYC<u>ATVGSGYYYCSGNPDL</u>WGQGTQVTVSS

Examined empirically identified clones in IgG fragment experiment

Cluster[1]	V Gene	J Gene	Length of CDR3[2]	Max % of appearance	Max bit score V[3]	Min bit score V[4]	Examined Clone	cDNA Sequence	Amino Acid Sequence, CDRs were undelined[2]
<u>Ig-33</u>	IGHV3S53	IGHJ4	5	0.601	329	285	Ig-L54	CAGGTGCAGCTCGTGGAGTCTGGGGGAGGCTTGGTGCAGGCTGGGGGGTCTCTTGAACTCTCCTGTGCAGCCTCTGGAGC<br>CGACTTCAGTTTCGATTATATGGCCTGGCACCGCCAGACTCCAGGGAAGCAGCGCGAGTTGGTCGCGGCTATTACTCCTC<br>ATCCTCATGGTATTACAAACTATGGGGGCTCCGTGAAGGGCCGATTCACCATCTCCAGAGACAACGCCAAGAAGACGGTG<br>TATCTACAAATGAACAACCTGAAACCTGATGACACAGGCGTCTATTACTGTTATGTCAGAGGGTACTGGGGCCAGGGGAC<br>CCAGGTCACCGTCTCCTCA	QVQLVESGGGLVQAGGSLELSCAAS<u>GADFSFDY</u>MAWHRQTPGKQRELVAA<u>ITPHPHGIT</u>NYGGSVK<br>GRFTISRDNAKKTVYLQMNNLKPDDTGVYYC<u>YVRGY</u>WGQGTQVTVSS
<u>Ig-69</u>	IGHV3S66	IGHJ6	20	0.347	486	425	Ig-L2477	CAGGTGCAGCTCGTGGAGTCTGGGGGAGGCTTGGTGCAGGCTGGGGGGTCTCTGAGACTCTCCTGTGCAGCCTCTGGATT<br>CAGTTTCACTTTCGATGATTTTACCATAGGCTGGTTCCGCCAGGCCCCAGGGAAGGAGCGTGAGGGGGTCTCATGTCTTA<br>GTAGTAGTGATGGTAGCACATACTATGAAGACTCCGTGAAGGGCCGATTCACCATCTCCAGTGACAACGCCAAGAACACG<br>GTGTATCTGCAAATGAACAGCCTGAAACCTGAGGACACGGCCGTTTATTACTGTGAAGCAGCCCTCGGTAGAAACTGGTC<br>GCCTGAGGACCTGTGTAGGGCTGACTTTGGTTCACGGGGCCAGGGGACCCAGGTCACCGTCTCCTCG	QVQLVESGGGLVQAGGSLRLSCAAS<u>GFSFTFDDFT</u>IGWFRQAPGKEREGVSC<u>LSSSDGST</u>YYEDSV<br>KGRFTISSDNAKNTVYLQMNSLKPEDTAVYYC<u>EAALGRNWSPEDLCRADFGS</u>RGQGTQVTVSS
<u>Ig-99</u>	IGHV3S61	IGHJ6	20	0.211	499	449	Ig-L252126	CAGGTGCAGCTCGTGGAGTCTGGGGGAGGCTTGGTGCAGCCTGGGGGGTCTCTGAGACTCTCCTGTGCAGCCTCTGGATT<br>CACTTTGGATTATTATGCCATAGGCTGGTTCCGCCAGGCCCCAGGGAAGGAGCGCGAGGGGGTTTTATGTATTAGTAGTA<br>GTGGTGATAGCACATACTATGCAGACTCCGTCAAGGGCCGATTCACCATCTCCAGAGACGTTGCCAAGAACACGGTGTAT<br>CTGCAAATGAACAGCCTGAAACCTGAGGACACGGCCGTTTATTACTGTGGGACAGATGCCCCCTACTATAGCGACAATTC<br>TCATCGTTGTCTGGCTGACTTTGGTTCCTGGGGCCAGGGGACCCAGGTCACCGTCTCCTCG	QVQLVESGGGLVQPGGSLRLSCAAS<u>GFTLDYYA</u>IGWFRQAPGKEREGVLC<u>ISSSGDST</u>YYADSVKG<br>RFTISRDVAKNTVYLQMNSLKPEDTAVYYC<u>GTDAPYYSDNSHRCLADFGS</u>WGQGTQVTVSS
<u>Ig-210</u>	IGHV3S53	IGHJ4	7	0.070	387	353	Ig-L15235	CAGGTGCAGCTCGTGGAGTCTGGGGGAGGCTTGGTGCAGGCTGGGGGGTCTCTGAGACTCTCCTGTGCAGCCTCTGGAAG<br>CATCTCTAGGGTCAATATCGTACGCTGGTACCGCCAGGCTCCAGGGAAGCAGCGCGACGTGGTCGCCGCCATTACTGGTA<br>GTGGTAGCGCGGATTATGCAGACTTCGCGAAGGGCCGATTCACCATCTCCATTGACAACGCCAAGAACACGGTGTATCTA<br>CAAATGAGCAGCCTGCAACCTGACGATACAGCCGCCTATTACTGTAACCTATTTCCAACTAACGATTGGGGCCAGGGGAC<br>CCAGGTCACCGTCTCCTCA	QVQLVESGGGLVQAGGSLRLSCAAS<u>GSISRVNI</u>VRWYRQAPGKQRDVVAA<u>ITGSGSA</u>DYADFAKGR<br>FTISIDNAKNTVYLQMSSLQPDDTAAYYC<u>NLFPTND</u>WGQGTQVTVSS

Examined clones in the predicted clusters

Cluster[1]	V Gene	J Gene	Length of CDRH-3[2]	Max % of appearance	Max bit score V[3]	Min bit score V[4]	Examined Clone	cDNA Sequence	Amino Acid Sequence, CDRs were undelined[2]
Ig-93	IGHV3-1	IGHJ7	13	0.232	536	508	Ig-L15542	CAGGTGCAGCTCGTGGAGTCTGGGGGAGGCTTGGTGCAGCCTGGGGGGTCTCTGAGACTCTCCTGTGCAGCCTCTGGATT<br>CACTTTTGATGATTATGCCATGAGCTGGGTCCGACAGGCTCCAGGGAAGGGGCTGGAGTGGGTCTCAGCTATTAGCTGGA<br>ATGGTGGTAGCACATACTATGCAGAATCCATGAAGGGCCGATTCACCATCTCCAGAGACAACGCCAAGAACACGCTGTAT<br>CTGCAAATGAACAGTCTGAAATCTGAGGACACGGCCGTGTATTACTGTGCAAAAGATCGTAGTAGCTGGCCAGGGGGCAT<br>GGACTACTGGGGCAAAGGGACCCTGGTCACCGTCTCCTCA	QVQLVESGGGLVQPGGSLRLSCAAS<u>GFTFDDYA</u>MSWVRQAPGKGLEWVSA<u>ISWNGGST</u>YYAESMKG<br>RFTISRDNAKNTLYLQMNSLKSEDTAVYYC<u>AKDRSSWPGGMDY</u>WGKGTLVTVSS

Ig-103	IGHV3S61	IGHJ6	24	0.204	510	433	Ig-L815	CAGGTGCAGCTCGTGGAGTCTGGGGGAGGCTTGGTGCACCCTGGGGGGTCTCTGAGACTCTCCTGTGCAGCCTCTGGATT<br>CACTTTGGGTTATTATGCCATAGTCTGGTTCCGCCAGGCCCCAGGGAAGGAGCGCGAGGGGGTCTCATGTATTAGTAGTG<br>GTGATGGTAGCACATACTATGCAGACTCCGTGAAGGGCCGATTCACCATCTCCAGAGACAATGCCAAGAACACGGTGTAT<br>CTGCAAATGAACAGCCTGAAACCTGAGGACACGGCCGTTTATGGCTGTGCGACAGATGGATCCCGGAATTACTACTACTC<br>CTGCGAACGTTTAATGTTTCGGCCGGCTGACTTTGCTTCCTGGGGCCAGGGGACCCAGGTCACCGTCTCCTCG	QVQLVESGGGLVHPGGSLRLSCAASGFTLGYYAIVWFRQAPGKEREGVSCISSGDGSTYYADSVKG<br>RFTISRDNAKNTVYLQMNSLKPEDTAVYGCATDGSRNYYYSCERLMFRPADFASWGQGTQVTVSS
Ig-126	IGHV3S53	IGHJ6	6	0.158	433	383	Ig-L1643	CAGGTGCAGCTCGTGGAGTCTGGGGGAGGCCAGGTGCAGGCTGGGGGGTCTCTGAGACTCTCCTGTGCAGCCTCTGGAAG<br>CATCTCAAGTATCCATGTCATGCGCTGGTACCGCCAGACTCCAGGGAACCAGCGCGAAGTGGTCGCAATGATTCTGGATA<br>GTGGTGTGACAAACTATGCAGACTCCGTGAAGGGCCGATTCACCATCTCCAGAGACAACGCCAAGAACACGCTGTATCTG<br>CAAATGAACAGCCTGAAACCTGAGGACACAGGTGTCTATTACTGTAATACATGGAACGGAGGCTGGGGCCAGGGGACCCA<br>GGTCACCGTCTCCCCA	QVQLVESGGGQVQAGGSLRLSCAASGSISSIHVMRWYRQTPGNQREVVAMILDSGVTNYADSVKGR<br>FTISRDNAKNTLYLQMNSLKPEDTGVYYCNTWNGGWGQGTQVTVSP
Ig-139	IGHV3S63	IGHJ2	22	0.139	440	412	Ig-L9713	CAGGTGCAGCTCGTGGAGTCTGGGGGAGGCTTGGTGCAGCCTGGGGGTTCTCTGAGACTCTCCTGTACAAACTCTGGATT<br>CACTTTGGAAGATTATGCCATAGGCTGGTTCCGCCAGGCCCCAGGGAAGGAGCGCGAGGGGGTCTCGTGTATGAGTACCA<br>ATGGTAGCACATACTATGCAGACTCCATGAAGGGCCGATTCACCATCTCCAGAGACAACGCCAAGAACACGGTGTATCTG<br>CAAATGAACAGCCTGAAACCTGAGGACACGGCCGTTTATTACTGTGCAGCAGAACAAACATGCGAATGGGGTTACTACGG<br>CGACTATGACGGATTGGGGTACCTCGAACGTTGGGGCCAGGGCACCCTGGTCACTGTCTCCTCA	QVQLVESGGGLVQPGGSLRLSCTNSGFTLEDYAIGWFRQAPGKEREGVSCMSTNGSTYYADSMKGR<br>FTISRDNAKNTVYLQMNSLKPEDTAVYYCAAEQTCEWGYYGDYDGLGYLERWGQGTLVTVSS
Ig-143	IGHV3S53	IGHJ6	9	0.131	520	448	Ig-L6897	CAGGTGCAGCTCGTGGAGTCTGGGGGAGGCTTGGTGCAGGCTGGGGGGTCTCTGAGACTCTCCTGTGCAGCCTCTGGAAG<br>CATCTCTAGTATAAATGCCATGGGCTGGTACCGCCAGGCTCCAGGGAAGCAGCGCGAGTTGGTCGCAGCTATTGCTTATG<br>GTGGAAACACAAACTATGCAAACTCCGTGAAGGGCCGATTCACCATCTCCAGAGACAACGCCAAGAACATGTTGTATCTG<br>CAAATGAACAACCTGAAACCTGAGGACACAGCCGTCTATTATTGTAATGCCGGAGTGTGGGAATTTGGTTCCTGGGGCCA<br>GGGGACCCAGGTCACCGTCTCCTCG	QVQLVESGGGLVQAGGSLRLSCAASGSISSINAMGWYRQAPGKQRELVAAIAYGGNTNYANSVKGR<br>FTISRDNAKNMLYLQMNNLKPEDTAVYYCNAGVWEFGSWGQGTQVTVSS
Ig-175	IGHV3S53	IGHJ6	16	0.097	521	422	Ig-L12393	CAGGTGCAGCTCGTGGAGTCTGGGGGAGGCTTGGTGCAGGCTGGGGGGTCTCTGAGACTCTCCTGTGCAGCCTCTGGAAG<br>CAGCATCAGTATTAATGCCGTGGGCTGGTACCGCCAGGCTCCAGGGAAGCAGCGCGAGTTGGTCGCACTTTTTACTAGTA<br>CTGGTAGCACGCACTATGGCGACTCCGTGAAGGGCCGATTCACCATTTCCAGAGACAACGCCAAGAACACGGTGTATCTG<br>CAAATGAACACCCTGAAACCTGAGGACACAGGCATCTATTACTGTAATTTAGGGCCCGACTGGGACTATAGCGACTATGC<br>CGACTCTGGTTCCTGGGGCCCGGGGACCCAGGTCACCGTCTCCTCA	QVQLVESGGGLVQAGGSLRLSCAASGSSISINAVGWYRQAPGKQRELVALFTSTGSTHYGDSVKGR<br>FTISRDNAKNTVYLQMNTLKPEDTGIYYCNLGPDWDYSDYADSGSWGPGTQVTVSS
Ig-245	IGHV3S61	IGHJ6	19	0.048	433	383	Ig-L13316	CAGGTGCAGCTCGTGGAGTCTGGGGGAGGCTTGGTGCAACCTGGGGGGTCTCTGAGACTCTCCTGTGCAGGCTCTGGATT<br>CACTTTGGATCATTATGCACTTGGCTGGTTCCGCCAGGCCCCAGGGAAGGAGCGCGAAGTGGTCTCATGTATTAGTAGTC<br>GTGATGGCACTACATACTATGCGAACTCCGTGAAGGGCCGATTCACCATCTCCAGAGACAAGAACGAGAACACGGTATAT<br>CTCCAAATGAACAACCTGCAACCTGAGGACACGGGCGTTTATTCTTGCGCGACGGATCTATTCGTGGCCGTTAGTAGGTG<br>GCAGTGTTCAACTAACTTTGATTCCTGGGGCCAGGGGACCCAGGTCACCGTCTCCGCG	QVQLVESGGGLVQPGGSLRLSCAGSGFTLDHYALGWFRQAPGKEREVVSCISSRDGTTYYANSVKG<br>RFTISRDKNENTVYLQMNNLQPEDTGVYSCATDLFVAVSRWQCSTNFDSWGQGTQVTVSA
Ig-275	IGHV3S53	IGHJ4	7	0.031	418	335	Ig-L20756	CAGGTGCAGCTCGTGGAGTCTGGGGGAGGCTTGGTGCAGGCTGGGGGGTCTCTGAGACTCTCCTGTGTAACCTCTGGAAT<br>CGTCTTCGAACTCAGTGGCATGGCCTGGTACCGCCAATCTCCAGGGAAGCAGCGCGAGTTCGTCGCCTCTATCACTAGTG<br>GAGGAAGTACAAATTATGGGAACTCCGCGAAGGGCCGATTCACCATTTCCAGAGACAACGCCAAGAATACTCTGTATCTG<br>CAAATGAACAGCCTGAAACCTGACGACACAGCCGTCTATCACTGTAATGGCCTTGGAAGTCCATACTGGGGCCGGGGGAC<br>CCAGGTCACCGTCTCCTCA	QVQLVESGGGLVQAGGSLRLSCVTSGIVFELSGMAWYRQSPGKQREFVASITSGGSTNYGNSAKGR<br>FTISRDNAKNTLYLQMNSLKPDDTAVYHCNGLGSPYWGRGTQVTVSS
EGFR-4	IGHV3S53	IGHJ4	11	2.703	416	333	EGFR-L3405<br>EGFR-S3476	CAGGTGCAGCTCGTGGAGTCTGGGGGAGGCTTGGTGCAGACTGGGGGGTCTCTGAGACTCTCCTGTGCAGCCTCTGAAAG<br>CAACCTCAGTCTCTATGTCATGGGCTGGTACCGCCAGGCTCCAGGGAGCCAGCGCGAGTTGGTCGCGATTATTACACCTG<br>GTGGTGGCACGCACTATGCAGACCTCGTGAAGGGCCGATTCACCATCTCCCGAGACAACGCCAAGAACACGGCATATCTG<br>CAAATGAACAGCCTGAAACCTGAGGACACGGCCGTCTACTCCTGTAATGCCCGACATAGAATATCTGGCGCGGAGTACTG<br>GGGCCAGGGGACCCAGGTCACCGTCTCCTCA	QVQLVESGGGLVQTGGSLRLSCAASESNLSLYVMGWYRQAPGSQRELVAIITPGGGTHYADLVKGR<br>FTISRDNAKNTAYLQMNSLKPEDTAVYSCNARHRISGAEYWGQGTQVTVSS
EGFR-8	IGHV3S53	IGHJ4	13	1.783	387	342	EGFR-S640	CAGGTGCAGCTCGTGGAGTCTGGGGGAGGCTTGGTGCAGCCTGGGGGGTCTCTGAGACTCTCCTGTGTAGCCTCTGGAAT<br>TGACTTCAATCTCTATAACATGGCCTGGTACCGCCAGACTCCAGGGAAGCAGCGCGAGTTGGTCGGCGTTGCTACTCCTG<br>GTGGTGGTACAAATTATGCGGACTCCGTGAAGGGCCGATTCACCATCTCCAGAGACAACGTCAAGAATATGGTGTTCCTG<br>CAAATGAACAATTTGGAACCTGAGGACACGGCCATCTATTACTGTTATGCGGGGGGACGGATCCCGATTCAGGCACGTGA<br>CTACTGGGGCCAGGGGACCCAGGTCACCGTCTCCTCA	QVQLVESGGGLVQPGGSLRLSCVASGIDFNLYNMAWYRQTPGKQRELVGVATPGGGTNYADSVKGR<br>FTISRDNVKNMVFLQMNNLEPEDTAIYYCYAGGRIPIQARDYWGQGTQVTVSS
EGFR-9	IGHV3S53	IGHJ7	7	1.719	411	327	EGFR-S36	CAGGTGCAGCTCGTGGAGTCTGGGGGAGGCTTGGTGCAGCCGGGGGGGTCTCTGTTACTCTCCTGTGCAAGCTCTGAAAA<br>CATCTTCAGACTCCGTGCCATGGCCTGGCACCGCCAGGCTCCAGGAAAAGAGCGCGAGTTGGTCGCAAGTATTTATACTA<br>GTGGTGACACAAACTATGCAGACTCCGTGAAGGGCCGATTCACCATCTCCAGAGACAACGCCAAGAACACGGTGGCTCTG<br>CAAATGAACAGCCTGAAACCTGAGGACACGGGCGTGTATTTTTGTAATATGAGAGGTACTGATTACTGGGGCAAAGGGAC<br>CCTGGTCACCGTCTCCTCA	QVQLVESGGGLVQPGGSLLLSCASSENIFRLRAMAWHRQAPGKERELVASIYTSGDTNYADSVKGR<br>FTISRDNAKNTVALQMNSLKPEDTGVYFCNMRGTDYWGKGTLVTVSS
EGFR-11	IGHV3S53	IGHJ4	14	1.670	438	405	EGFR-L7<br>EGFR-S1361	CAGGTGCAGCTCGTGGAGTCTGGGGGGGGCTTGGTGCAGCCTGGGGGGTCTCTGAGACTCTCCTGTGCAGCCTCCGGATA<br>CATCTTCAGTGCGTATACCATGGGCTGGTACCGCCAGGCTCCAGGGAAGCAGCGCGAGTTGGTCGCATATATGACTAGCA<br>GTGGTGACGCAAATTATGTAGACTCCGTGAAGGGCCGATTCACCATCTCCAGAGACAACGCCAAGAAGACGGTGTATCTG<br>CAAATGAACAGCCTGAAACCTGAGGACACGGCCGTCTATTACTGTAATCGGGATACGGGTATGGGCCTTACTAAGGTGAA<br>TGACTCTTGGGGCCAGGGGACCCAGGTCACCGTCTCCTCA	QVQLVESGGGLVQPGGSLRLSCAASGYIFSAYTMGWYRQAPGKQRELVAYMTSSGDANYVDSVKGR<br>FTISRDNAKKTVYLQMNSLKPEDTAVYYCNRDTGMGLTKVNDSWGQGTQVTVSS
EGFR-14	IGHV3S65	IGHJ4	20	1.369	516	455	EGFR-L39	CAGGTGCAGCTCGTGGAGTCTGGGGGAGGCTTGGTGCAGCCTGGGGGGTCTCTGAGACTCTCCTGTGCAGCCTCTGGACT<br>CACTTTGGATTATTATGCCATAGGCTGGTTCCGCCAGGCCCCAGGGAAGGAGCGTGAGGGGGTCTCATGTATTAGTAGTA<br>GTGATGGTAGCACATACTATGCAGACTCCGTGAAGGGCCGATTCACCATCTCCAGAGACAACGCCAAGAACACGGTGTAT<br>CTGCAAATGAACAGCCTGAAACCTGAGGACACAGCCGTTTATTACTGTGCAGCCTCGGGTAGTGGTAGTGCCTACTACGC<br>ACTCCTTCGTCAATATGAGTATGACTACTGGGGCCAGGGGACCCAGGTCACCGTCTCCTCA	QVQLVESGGGLVQPGGSLRLSCAASGLTLDYYAIGWFRQAPGKEREGVSCISSSDGSTYYADSVKG<br>RFTISRDNAKNTVYLQMNSLKPEDTAVYYCAASGSGSAYYALLRQYEYDYWGQGTQVTVSS

EGFR-19	IGHV3S53	IGHJ4	9	0.813	446	340	EGFR-L194	CAGGTGCAGCTCGTGGAGTCTGGGGGAGGCTTGGTGCAGCCTGGGGGGTCTCTGAGACTCTCCTGTACAGCCTCTGGAAC<br>AATCACCACATTCTATGCCATGGCCTGGTACCGCCAGGGTCCAGGGAAGCAGCGCGAGCAGGTCGCACATATTAGTAGTG<br>GCGGTTTTACAAATTATCCAGAGTCCGTGAAGGGCCGATTCACCATCTCCAGAGACAGCGCCGTGAACACGCTGTATCTG<br>CAAATGAACAGCCTGAAACCTGAGGATACGGCCGTCTATTACTGTAATGTAGAGAGGTACGGCTTTATGTACTGGGGCCA<br>GGGGACCCAGGTCACCGTCTCCTCA	QVQLVESGGGLVQPGGSLRLSCTASGTITTFYAMAWYRQGPGKQREQVAHISSGGFTNYPESVKGR<br>FTISRDSAVNTLYLQMNSLKPEDTAVYYCNVERYGFMYWGQGTQVTVSS
EGFR-20	IGHV3S53	IGHJ4	15	0.745	455	388	EGFR-L4879	CAGGTGCAGCTCGTGGAGTCTGGGGGAGACTTGGTGCAGCCTGGGGGGTCTCTGAGACTCGCCTGTACAGCCCGTGGAAG<br>CATCTCCTGGATCTATACCATGGGCTGGTACCGCCAGGCTCCAGGGAAGCAGCGCGAGTTGGTCGCACGTATTACTAATG<br>GCGGGAACGAAAACTACTCAGACTCCGTGAAGGGCCGGTTCACCATCTCCAGAGACGTCGCCAAGAACACGGTGTATCTG<br>CAAATGAACAGCCTGAAACCTGAGGACACGGCCGTCTATTATTGTAATGCAGACATAAGGACCCGCACGGATTTGATACG<br>GGGAGACTACTGGGGCCAGGGGACCCAGGTCACCGTCTCCTCA	QVQLVESGGDLVQPGGSLRLACTARGSISWIYTMGWYRQAPGKQRELVARITNGGNENYSDSVKGR<br>FTISRDVAKNTVYLQMNSLKPEDTAVYYCNADIRTRTDLIRGDYWGQGTQVTVSS
EGFR-23	IGHV3S53	IGHJ4	13	0.609	416	361	EGFR-L109	CAGGTGCAGCTCGTGGAGTCTGGGGGAGGCTTGGTGCAGCCTGGGGGGTCTCAAAGACTCTCCTGTGCAGCCTCTGGACG<br>CTCAGTCAGTTTCGCGACCATGGCCTGGTACCGCCAGGCTCCAGGGAAGCAGCGCGAATTGGTCGCATTTATTACTAACA<br>GTGGTAACACAAACTATGCAGACTCCGTGAAGGGCCGATTCACCATCTCCCGAGACAACGCCAAGAACACGTGGTATCTG<br>CAAATGAACGACCTGAGACCTGAGGACACGGCCGTCTATTACTGTAATGCAAATTCCCTGGTTGGGCTACGAATTGATAC<br>GTACTGGGGCCAGGGGACCCAGGTCACCGTCTCCTCA	QVQLVESGGGLVQPGGSQRLSCAASGRSVSFATMAWYRQAPGKQRELVAFITNSGNTNYADSVKGR<br>FTISRDNAKNTWYLQMNDLRPEDTAVYYCNANSLVGLRIDTYWGQGTQVTVSS
EGFR-24	IGHV3S65	IGHJ4	21	0.596	472	411	EGFR-S3849	CAGGTGCAGCTCGTGGAGTCTGGGGGAGGCTTGGTGCAGCCTGGGGGGTCTCTGAGACTCTCCTGTGCAGCCTCTGGATT<br>CACTTTGAATTATTATGCCATAGACTGGATCCGCCAGGCCCCAGGGAAGGAGCCTGAGGGGGTCTCATGTATTAGTGGTC<br>GTGATGGTAACGCATACTATGCAGATTCCGTGAAGGGCCGATTCACCGTCTCCAGAGACAACGCCGAGAACACGGTGTAT<br>CTGCAGATGAACAGCCTGAAACTTGAGGACACAGCCGTTTATCACTGTGCAGCCCTCCTGGGCCGACCGTCGTGTCCTAC<br>GGCTTGGGCTAGCTCACGACCTTATAACATAAGGGGCCAGGGGACCCAGGTCACCGTCTCTTCA	QVQLVESGGGLVQPGGSLRLSCAASGFTLNYYAIDWIRQAPGKEPEGVSCISGRDGNAYYADSVKG<br>RFTVSRDNAENTVYLQMNSLKLEDTAVYHCAALLGRPSCPTAWASSRPYNIRGQGTQVTVSS
EGFR-25	IGHV3S65	IGHJ4	19	0.579	405	333	EGFR-L67	CAGGTGCAGCTCGTGGAGTCTGGGGGAGGCTTGGTGCAGCCTGGGGGGTCTCTAAGACTCTCCTGTGTAATCTCTGGTTT<br>CAATTTGGAATATTTAACCGTGGGCTGGTTCCGCCTGGCCCCAGGGAAGGAGCGTGAGGGGATCTCATGCATTAGTAGAA<br>GTGCCACTAACACAGTCTATGCAGACTCCGTGAAGGACCGATTCACCATCTCCAGAGACAACGCCAAGAACACGGTGTAT<br>CTGCAAATGAACGTCCTGAAACCTGAAGACGTAGCCGCTTATTACTGTGCAGCCTACCAGGACGGCTTCAATGCTTGTGC<br>CTTATCCGCTAGGGACTACGCCTATTGGGGCCAGGGGACCCGGGTCACCGTCTCCTCA	QVQLVESGGGLVQPGGSLRLSCVISGFNLEYLTVGWFRLAPGKEREGISCISRSATNTVYADSVKD<br>RFTISRDNAKNTVYLQMNVLKPEDVAAYYCAAYQDGFNACALSARDYAYWGQGTRVTVSS
EGFR-34	IGHV3S53	IGHJ4	11	0.391	411	366	EGFR-S838	CAGGTGCAGCTCGTGGAGTCTGGGGGAGGCTTGGTGCAGCCTGGGGGGTCTCTGAGACTCTCCTGTGCAGCCTTTGGAAG<br>CATAGGCGATCTCTATACCATGGGCTGGTACCGCCAGGCTCCAGGGAAGCAGCGCGAGTTGGTCGCAGATATTACTAGAG<br>ATGGTACCACAAACTATGGAGAATACGTGAAGGACCGATTCACCATCTCCAGAGACAACGCCAAGAACACGGTGGATCTG<br>CAAATGAGCAGCTTGAAATTTGAGGACACGGCCGTCTATATCTGTAATGCAAGAGCATGGACTGGATTGAGGTCGCACTG<br>GGGCCAGGGGACCCAGGTCACCGTCTCCTCA	QVQLVESGGGLVQPGGSLRLSCAAFGSIGDLYTMGWYRQAPGKQRELVADITRDGTTNYGEYVKDR<br>FTISRDNAKNTVDLQMSSLKFEDTAVYICNARAWTGLRSHWGQGTQVTVSS
EGFR-46	IGHV3S53	IGHJ4	11	0.265	449	394	EGFR-S1620	CAGGTGCAGCTCGTGGAGTCTGGGGGAGGCTTGGTACAACCTGGGGGGTCTCTGAGACTCTCCTGTGCAGCCTCTGGAAT<br>TAGCTTCAATCTCTATGTCATGGGCTGGTACCGCCAGGCTCCAGGGAAGCAGCGCGAGTTGGTCGCACTTATTACTCCTG<br>GTGGAGGCACAAACTATGCAGACTCCGTGAAGGGCCGATTCACCATCTCCCTAGACAACGCCAAGAACACGGTGTCTCTG<br>CAAATGAACAGCCTGGAACCTGAGGACACGGCCGTCTATTACTGTAATGCACGGCACCGGATTACCTCAAATAACTTGTG<br>GGGCCAGGGGACCCAGGTCACCGTCTCCTCA	QVQLVESGGGLVQPGGSLRLSCAASGISFNLYVMGWYRQAPGKQRELVALITPGGGTNYADSVKGR<br>FTISLDNAKNTVSLQMNSLEPEDTAVYYCNARHRITSNNLWGQGTQVTVSS

1 Hit- or empirically identified clusters were underlined.
2 CDRs were defined by IMGT numbering scheme.
3 Highest bit score for the V gene of the clones in the cluster.
4 Lowest bit score for the V gene of the clones in the cluster.

SUPPLEMENTARY TABLE S3 | Sequences and kinetic parameters in SPR analysis of examined clones in cluster Ig-7

Clone	cDNA sequence	Amino acid Sequence with CDRs underlined[1]	k_a(1/Ms)	SE (k_a)	k_d(1/s)	SE (k_d)	K_D(M)	χ^2(RU^2)
Ig-S506	CAGGTGCAGCTCGTGGAGTCTGGGGGAGGCTTGGTGCAGGCTGGGGGGTCTCTGAGACTCTCCTGTGCAGCCTCTGGATTCACTTTCGATGATTATGT<br>CATAGGCTGGTTCCGCCAGGCCCCAGGGAAGGAGCGCGAGGGGGTCTCATGTATTAGTAGTAGTGATGGTAGCACAAACTATGCAGACTCCGTGAAGG<br>GCCGATTCACCATCTCCAGTGACAACGCCAAGAACACGGTGTATCTGCAAATGAACAGCCTGAAACCTGAGGACACGGCCGTTTATTACTGTGCAGAG<br>GGCCCCACGGTCCTAGACGGATGTATATACGATAGTGGTAGTTACTATTTTTCCTGGGGCCAGGGGACCCAGGTCACCGTCTCCTCG	QVQLVESGGGLVQAGGSLRLSCAASGFTFDDYVIGWFRQAPGKEREGVSCISSSDGSTNYADSVK<br>GRFTISSDNAKNTVYLQMNSLKPEDTAVYYCAEGPTVLDGCIYDSGSYYFSWGQGTQVTVSS	2.97×10^{4}	1.90×10^{2}	7.30×10^{-3}	4.90×10^{-5}	2.46×10^{-7}	1.73
Ig-S537	CAGGTGCAGCTCGTGGAGTCTGGGGGAGGCTTGGTGCAGGCTGGGGGGTCTCTGAGACTCTCCTGTGCAGCCTCTGGATTCACTTTCGATGATTATGT<br>CGTAGCCTGGTTCCGCCAGGCCCCAGGGAAGGAGCGTGAGGGGGTCTCATGTATTAGTAGTAGTGATGGTAGCACATACTCTGCAAGCTCCGTGAAGG<br>GCCGATTCACCATCTCCAGTGACAACGCCAAGAACACGGTGTATCTGCAAATGAACGACCTGAAACCTGAGGACACGGCCGTTTATTACTGTGCAGAA<br>GGTCCCGTGGTCCTAGACGGATGTATATTGGATAGTGAAAGTTACTATTTTTCCTGGGGCCAGGGGACCCAGGTCACCGTCTCCTCG	QVQLVESGGGLVQAGGSLRLSCAASGFTFDDYVVAWFRQAPGKEREGVSCISSSDGSTYSASSVK<br>GRFTISSDNAKNTVYLQMNDLKPEDTAVYYCAEGPVVLDGCILDSESYYFSWGQGTQVTVSS	1.80×10^{5}	1.80×10^{2}	2.54×10^{-4}	9.70×10^{-7}	1.42×10^{-9}	2.52
Ig-S675<br>Ig-L2848	CAGGTGCAGCTCGTGGAGTCTGGGGGAGGCTTGGTGCAGGCTGGGGGGTCTCTGAGACTCTCCTGTGCAGCCACTGGATTCACTTTCGATGATTATGT<br>CATAGGCTGGTTCCGCCAGGCCTCAGGGAAGGAGCGCGAGGGGGTCTCATGTATTCGTAGTAGTGATGGTGACACATACTATGCAGACTCCGTGAAGG<br>GCCGATTCACCGTCTCCAGTGACAACGCCAAGAACACGGTGTATCTGCAAATGAACAGCCTGAAGCCTGAGGACACGGCCGTTTATTACTGTGCAGAA<br>GGACCCACGGTCCTAGACGGATGTATCGTCGATAGTGAAAGTTACTATTTTTCTTGGGGCCAGGGGACCCAGGTCACCGTCTCCTCG	QVQLVESGGGLVQAGGSLRLSCAATGFTFDDYVIGWFRQASGKEREGVSCIRSSDGDTYYADSVK<br>GRFTVSSDNAKNTVYLQMNSLKPEDTAVYYCAEGPTVLDGCIVDSESYYFSWGQGTQVTVSS	3.03×10^{5}	2.40×10^{2}	4.71×10^{-5}	7.50×10^{-7}	1.55×10^{-10}	1.68
Ig-S709<br>Ig-L25474	CAGGTGCAGCTCGTGGAGTCTGGGGGAGGCTTGGTGCAGGCTGGGGGGTCTCTGAGACTCTCCTGTGCAGCCTCTGGATTCACTCCCGATGATGATGT<br>CATAGGCTGGTTCCGCCAGGCCCCAGGGAAGGAGCGCGAGGGGGTCTCATGTATTAGTAGTAGTGATGGTCGCACAAACTATGCGGAGTCCGTGAAGG<br>GCCGATTCACCATCTCCAGCGACAACGCCAAGAACACGGTGTATCTGCAAATGAACAGCCTGCAACCTGAGGACACGGCCGTTTATTACTGTGCAGCA<br>GGACCGACGGTCATAGACGGATGTATATACGATAGTGAGAGTTACTATTTTTCCTGGGGCCAGGGGACCCAGGTCACCGTCTCCTCG	QVQLVESGGGLVQAGGSLRLSCAASGFTPDDDVIGWFRQAPGKEREGVSCISSSDGRTNYAESVK<br>GRFTISSDNAKNTVYLQMNSLQPEDTAVYYCAAGPTVIDGCIYDSESYYFSWGQGTQVTVSS	7.43×10^{3}	6.6	2.83×10^{-4}	7.40×10^{-7}	3.81×10^{-8}	0.981
Ig-S768	CAGGTGCAGCTCGTGGAGTCTGGGGGAGGCTTGGTGCAGGCTGGGGGGTCTCTGAGACTCTCCTGTGCAGCCTCTGGATTCACTTTCGATGATTGGAA<br>CATAGGCTGGTTCCGCCAGGCCCCAGGGAAGGAGCGTGAGGGGGTCTCCTGTTTTAGTAGTACTGATGGTCGCACCGACTATGCAGACTCCGTGAAGG<br>GCCGATTCACCATCTCCAGTGACAACGCCAAGAACACGGTGTATCTGCAAATGAACAGCCTGAAACCTGAGGACACGGCCGTTTATTACTGTGCAGAA<br>GGACCCACGGTCATAGACGGATGTATATACGATAGTGAAAGTTACTATTTTTCCTGGGGCCAGGGGACCCAGGTCACCGTCTCCTCG	QVQLVESGGGLVQAGGSLRLSCAASGFTFDDWNIGWFRQAPGKEREGVSCFSSTDGRTDYADSVK<br>GRFTISSDNAKNTVYLQMNSLKPEDTAVYYCAEGPTVIDGCIYDSESYYFSWGQGTQVTVSS	1.94×10^{4}	21.0	1.13×10^{-4}	1.20×10^{-6}	5.84×10^{-9}	7.98
Ig-S1139<br>Ig-L27626	CAGGTGCAGCTCGTGGAGTCTGGGGGAGGCTTGGTGCAGGCTGGGGGGTCTCTGAGACTCTCCTGTGCAGCCTCTGGATTCACTTTCGATGATTATGT<br>CATAGGCTGGTTCCGCCAGGCCCCAGGGAAGGAGCGCGAGGGGGTCTCATGTATTAGTAGTAGTGATGGTAGCACATACTATGCAGACTCCGTGAAGG<br>GCCGATTCACCATCTCCAGTGACAACGCCAAGAACACGGTGTATCTGCAAATGAACAGCCTGAAACCTGAGGACACGGCCGTTTATTACTGTGCAGAA<br>GGACCCACGGTCCTAGACGGATGTATAGTGGATAGTGGTAGTTACTATTTTTCCTGGGGCCAGGGGACCCAGGTCACCGTCTCCTCG	QVQLVESGGGLVQAGGSLRLSCAASGFTFDDYVIGWFRQAPGKEREGVSCISSSDGSTYYADSVK<br>GRFTISSDNAKNTVYLQMNSLKPEDTAVYYCAEGPTVLDGCIVDSGSYYFSWGQGTQVTVSS	2.37×10^{4}	2.40×10^{2}	5.54×10^{-3}	5.90×10^{-5}	2.33×10^{-7}	3.78
Ig-L1511<br>Ig-S6937	CAGGTGCAGCTCGTGGAGTCTGGGGGAGGCTTGGTGCAGGCTGGGGGGTCTCTGAGACTCTCCTGTGCAGCCTCTGGATTCACTTTCGATGATTATGT<br>CATAGGCTGGTTCCGCCAGGCCCCAGGGAAGGAGCGCGAGGGGGTCTCATGTATTAGTAGTAGTGATGGTAGCACATACTATGCAGACTCCGTGAAGG<br>GCCGATTCACCATCTCCAGTGACAACGCCAAGAACACGGTGTATCTGCAAATGAACAGCCTGAAACCTGAGGACACGGCCGTTTATTACTGTGCAGAA<br>GGACCCACGGTCCTAGACGGATGTATATTGGATAGTGGTAGTTACTATTTTTCCTGGGGCCAGGGGACCCAGGTCACCGTCTCCTCG	QVQLVESGGGLVQAGGSLRLSCAASGFTFDDYVIGWFRQAPGKEREGVSCISSSDGSTYYADSVK<br>GRFTISSDNAKNTVYLQMNSLKPEDTAVYYCAEGPTVLDGCILDSGSYYFSWGQGTQVTVSS	1.63×10^{5}	8.90×10^{2}	1.33×10^{-3}	5.80×10^{-6}	8.15×10^{-9}	3.83
Ig-L2487<br>Ig-S2363	CAGGTGCAGCTCGTGGAGTCTGGGGGAGGCTTGGTGCCGGCTGGGGGGTCTCTGAGACTCTCCTGTGTAGCCTCTGGATTCACTTTCGATGATGGCAC<br>CATAGGCTGGTTCCGCCAGGCCCCAGGGAAGGAGCGTGAGGGGGTCTCATGTATTAGTCGTAGTGATGGTGGCACCAACTTTGCAGACTCCGTGAAGG<br>GCCGATTCACCATCTCCAGTGACAACGCCAAGAACACGGTGTATCTGCAAATGAACAGCCTGAAACCTGAGGACACGGCCGTTTATTACTGTGCAGAA<br>GGACCCACGGTCCTAGACGGATGTATATACGATAGTGAGAGTTACTATTTTTCCTGGGGCCAGGGGACCCAGGTCACCGTCTCCTCG	QVQLVESGGGLVPAGGSLRLSCVASGFTFDDGTIGWFRQAPGKEREGVSCISRSDGGTNFADSVK<br>GRFTISSDNAKNTVYLQMNSLKPEDTAVYYCAEGPTVLDGCIYDSESYYFSWGQGTQVTVSS	2.55×10^{5}	3.00×10^{2}	3.74×10^{-4}	1.30×10^{-6}	1.47×10^{-9}	4.76
Ig-L2754	CAGGTGCAGCTCGTGGAGTCTGGGGGAGGCTTGGTGCAGGCTGGGGGGTCTCTGAGACTCTCCTGTACAGCCTCTGGATTCACTTTCGATGATCCTGT<br>CATAGGCTGGTTCCGTCAGGCCCCAGGGAAGGAGCGCGAGGGGGTGTCATGTATTAGTAGTAGTGATGGTAGGACATACAACGCAGACAACGTGAAGG<br>GCCGATTCACCATCTCCAGTGACAACGCCAAGAACACGGTGTATCTGCAAATGGACAGCCTGAAACCTGAGGACACGGCCGTTTATTACTGTGCAGAA<br>GGACCCACGGTAGTTGACGGATGTATCCTCGATAGTGAAAGTTACTATTTTTCCTGGGGCCAGGGGACCCAGGTCACCGTCTCCTCG	QVQLVESGGGLVQAGGSLRLSCTASGFTFDDPVIGWFRQAPGKEREGVSCISSSDGRTYNADNVK<br>GRFTISSDNAKNTVYLQMDSLKPEDTAVYYCAEGPTVVDGCILDSESYYFSWGQGTQVTVSS	4.16×10^{5}	3.80×10^{2}	7.52×10^{-5}	9.30×10^{-7}	1.81×10^{-10}	3.55
Ig-L2796<br>Ig-S10289	CAGGTGCAGCTCGTGGAGTCTGGGGGAGGCTTGGTGCAGACTGGGGGGTCTCTGAGACTCTCCTGTGCAGCCTCTGGATTCACGGAAAATGAAGATGT<br>CATAGGCTGGTTCCGCCAGGCCCCAGGGAAGGAGCGTGAGGGGGTCTCATGTATTAGTAATAGTGATGGTAGGGCAGACTATGCAGACTCCGTGAAGG<br>GCCGATTCACCATCTCCAAAGACAACGCCAAGAAAACGGTGTATCTGCACATGAACAGCCTGAAACCTGAGGACACGGCCGTTTATTACTGTGCAGCA<br>GGACCCACGGTCCTAGACGGATGTATATACTATAGTGAAAGTTACTATTTTTTCTGGGGCCAGGGGACCCAGGTCACCGTCTCCTCG	QVQLVESGGGLVQTGGSLRLSCAASGFTENEDVIGWFRQAPGKEREGVSCISNSDGRADYADSVK<br>GRFTISKDNAKKTVYLHMNSLKPEDTAVYYCAAGPTVLDGCIYYSESYYFFWGQGTQVTVSS	1.64×10^{5}	2.40×10^{2}	8.27×10^{-4}	1.40×10^{-6}	5.03×10^{-9}	2.60
Ig-S3320<br>Ig-L9619	CAGGTGCAGCTCGTGGAGTCTGGGGGAGGCTTGGTGCAGGCTGGGGGGTCTCTGAGACTCTCCTGTGCAGCCTCTGCATTCACTTTCGATGATTATAT<br>TGTTGGCTGGTTCCGCCAGGCCCCAGGGAAGGAGCGTGAGGGAGTCTCATGTATCCGTAGTAGTGATGGTAGCACCAACTATGCAGACTCCGTGAAGG<br>GCCGTTTCGCCATCTCCAGTGACAACGCCAAGAACACGGTGTATCTGCAAATGAACAGCCTGAAGCCTGAGGACACGGCCGTTTATTACTGTGCAGAC<br>GGACCCACGGTCCTAGATGGATGCATATTGGATAGTGAAAGTTACTATTTTTCCTGGGGCCAGGGGACCCAGGTCACCGTCTCCTCG	QVQLVESGGGLVQAGGSLRLSCAASAFTFDDYIVGWFRQAPGKEREGVSCIRSSDGSTNYADSVK<br>GRFAISSDNAKNTVYLQMNSLKPEDTAVYYCADGPTVLDGCILDSESYYFSWGQGTQVTVSS	3.15×10^{4}	32.0	9.47×10^{-5}	1.10×10^{-6}	3.00×10^{-9}	4.75
Ig-S6584	CAGGTGCAGCTCGTGGAGTCTGGGGGAGGCTTGGTGCAGGCTGGGGGGTCTCTGAGACTCTCCTGTGCAGCCTCTGGATTCACTTTCGATGATTATAT<br>CGTAGGCTGGTTCCGCCAGGCCCCAGGGAAGGAGCGTGAGGGGGTCTCATGTATTAGGAGTAGTGATGGTAGTACCAACTATGCAGACTCCGTGAAGG<br>GCCGATTCACCGTCTCCAGTGACAACGCCAAGAACACGGTGTATCTGCAAATGAACAGCCTGAAACCTGAGGACACGGCCGTTTATTACTGTGCAGAA<br>GGACCCACGGTCCTAGACGGATGTATATACGATAGTGAAAGTTACTATTTTTCCTGGGGCCAGGGGACCCAGGTCACCGTCTCCTCG	QVQLVESGGGLVQAGGSLRLSCAASGFTFDDYIVGWFRQAPGKEREGVSCIRSSDGSTNYADSVK<br>GRFTVSSDNAKNTVYLQMNSLKPEDTAVYYCAEGPTVLDGCIYDSESYYFSWGQGTQVTVSS	3.69×10^{4}	52.0	2.21×10^{-4}	1.50×10^{-6}	5.99×10^{-9}	9.31
Ig-S8492	CAGGTGCAGCTCGTGGAGTCTGGGGGAGGCTTGGTGCAGGCTGGGGGGTCTCTGAGACTCTCCTGTGCAGCCTCTGGATTCACTTTCGATGATTATGT<br>TATAGGCTGGTTCCGCCAGGCCCCAGGGAAGGAGCGTGAGGGGGTCTCATGTATTAGTAGTAGTGATGGTAGGACAGACTATGCAGACTCCGTGAAGG<br>GCCGATTCACCATCTCCAGTGACAACGCCAAGAACACGGTGTATCTGCAAATGAACAGCCTGAAACCTGAGGACACGGCCGTTTATTACTGTGCAGCA<br>GGACCCACGGTCCTAGACGGATGTATATACTATAGTGGTAGTTACTATTTTTCCTGGGGCCAGGGGACCCAGGTCACCGTCTCCTCG	QVQLVESGGGLVQAGGSLRLSCAASGFTFDDYVIGWFRQAPGKEREGVSCISSSDGRTDYADSVK<br>GRFTISSDNAKNTVYLQMNSLKPEDTAVYYCAAGPTVLDGCIYYSGSYYFSWGQGTQVTVSS	1.99×10^{3}	73.0	2.45×10^{-4}	1.10×10^{-6}	1.23×10^{-7}	0.302
Ig-L15236	CAGGTGCAGCTCGTGGAGTCTGGGGGAGGCTTGGTGCAGGCTGGGGGGTCTCTGAGACTCTCCTGTGCAGCCTCTGGATTCACTGTCGGTGATGATGC<br>CATAGGCTGGTTCCGCCAGGCCCCAGGGAAGGAGCGTGAGGGGGTCTCATGTATTCTTAGTAGTGATGGTCGGACAGACTATGCAGACTCCGTGAAGG<br>GCCGATTCACCATCTCCAGTGACAACGCCAAGAACACGGTGTATCTGCAAATGGACAGCCTGAAACCTGAGGACACGGCCGTTTATTACTGTGCAGCA<br>GGACCCACGGTCCTAGACGGATGTATATACTATAGTGAGAGTTACTATTTTTCCTGGGGCCAGGGGACCCAGGTCACCGTCTCCTCG	QVQLVESGGGLVQAGGSLRLSCAASGFTVGDDAIGWFRQAPGKEREGVSCILSSDGRTDYADSVK<br>GRFTISSDNAKNTVYLQMDSLKPEDTAVYYCAAGPTVLDGCIYYSESYYFSWGQGTQVTVSS	2.16×10^{5}	8.70×10^{2}	2.25×10^{-3}	8.60×10^{-6}	1.04×10^{-8}	1.74

1 CDRs were defined by IMGT numbering scheme.
